# Widening educational inequalities in type 2 diabetes risk and mediators across successive generations: a prospective cohort study

**DOI:** 10.3389/fpubh.2026.1893269

**Published:** 2026-10-06

**Authors:** Zehao Zhao, Mengge Zhou, Changsheng Ma, Tianyi Wang, Libo Zhao, Xiliang Zhao, Quan Li, Yaodong Ding, Yang Zhang, Li Lin, Wenjie Wang, Jiayi Han, Yingxun Bai, Yong Zeng

**Affiliations:** 1 Department of Cardiology, Beijing Anzhen Hospital, Capital Medical University, Tongzhou, Beijing, China; 2 National Clinical Research Center for Cardiovascular Disease, Tongzhou, Beijing, China; 3 Beijing Institute of Heart, Lung and Blood Vessel Diseases, Tongzhou, Beijing, China; 4 Department of Epidemiology and Biostatistics, School of Basic Medicine, Institute of Basic Medical Sciences, Chinese Academy of Medical Sciences, Peking Union Medical College, Beijing, China

**Keywords:** educational inequalities, generational differences, mediation analysis, Relative Index of Inequality, type 2 diabetes mellitus

## Abstract

**Background:**

Lower educational attainment has been associated with incident T2DM and adverse outcomes among people with established diabetes, but these stages have largely been studied separately. Whether education-related inequalities span the trajectory from T2DM onset to subsequent cardiovascular disease (CVD) and death, and whether these inequalities differ across birth cohorts, remains uncertain.

**Methods:**

This study included participants from the UK Biobank who were free of diabetes at baseline. Participants were stratified into three birth cohorts (pre-1949, 1950–1959, and post-1960) to investigate intergenerational differences in educational inequalities. We employed Fine-Gray proportional subdistribution hazards models and multi-state competing risks models to evaluate the impact of education on the risk of incident T2DM and its subsequent progression to cardiovascular disease (CVD) and mortality. The Relative Index of Inequality (RII) was calculated to quantify the magnitude of disparities. Pathway analysis was conducted to estimate the extent to which socioeconomic, behavioral, and metabolic factors explained the association between educational attainment and T2DM risk.

**Results:**

During a median follow-up of 16.5 years, 19,931 incident cases of T2DM were documented among the 460,797 included participants. Primary education or less was associated with increased risks of incident T2DM (HR 1.92, 1.85–1.99), progression to CVD (HR 1.33, 1.30–1.37), and mortality (HR 1.53, 1.49–1.57) compared with college education, and these disparities widened across successive birth cohorts. Overall, the adjusted RII was 2.37 (2.25–2.50), increasing from 2.31 (2.16–2.47) in the pre-1949 cohort to 2.53 (2.29–2.79) in the 1950–1959 cohort and 2.53 (2.18–2.93) in the post-1960 cohort. Socioeconomic, metabolic, and behavioral factors mediated a substantial proportion of the educational inequality, accounting for 42.2% (39.2%−45.2%), 42.6% (38.2%−47.3%), and 20.5% (18.9%−22.2%), respectively.

**Conclusion:**

Education-related inequalities extend from T2DM incidence to subsequent progression and have widened across successive birth cohorts. Socioeconomic, behavioral, and metabolic factors mediated a substantial proportion of these disparities.

## Graphical Abstract

**Figure:**
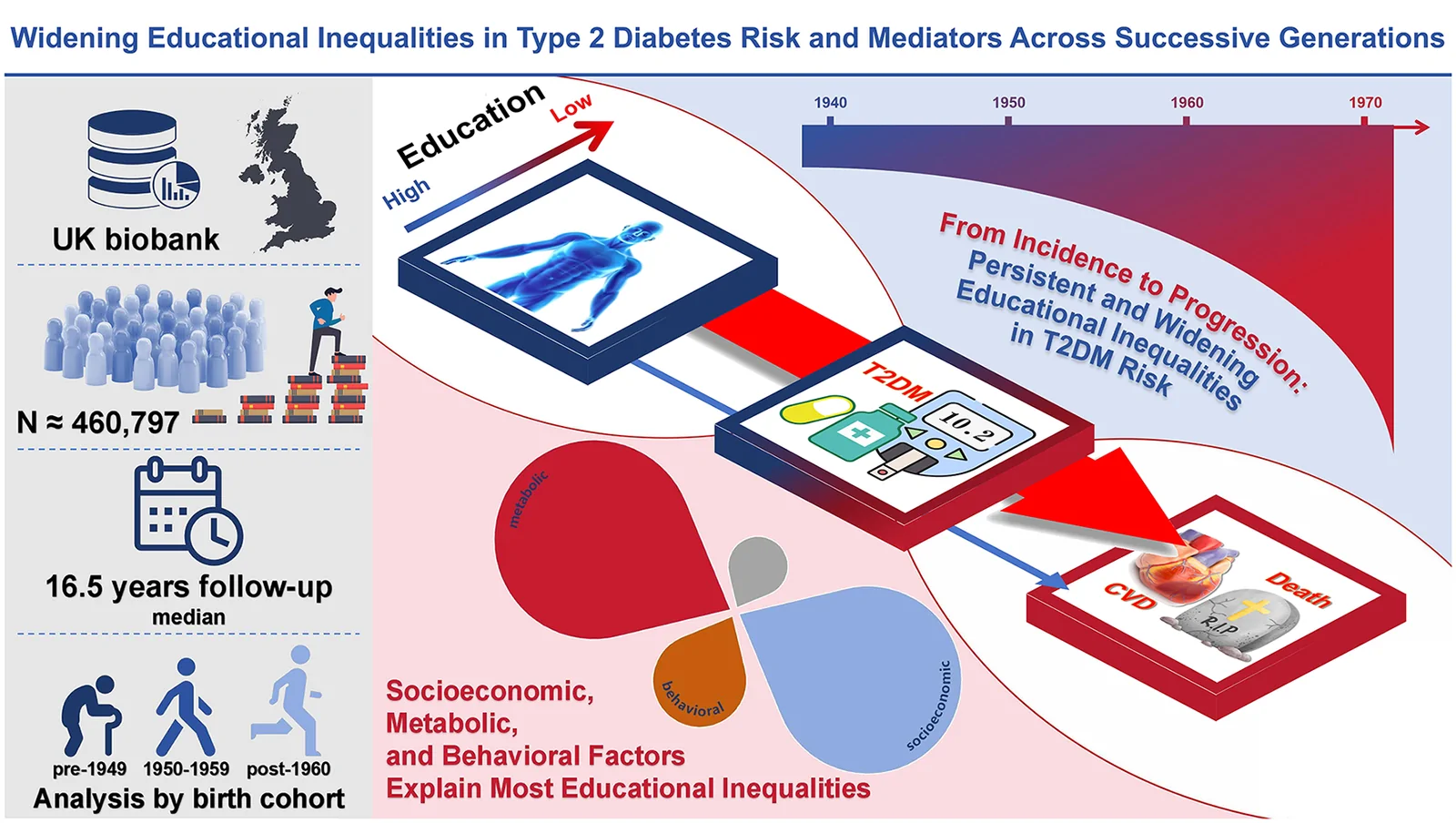
Widening educational inequalities in type 2 diabetes risk and mediators across successive generations. Using UK Biobank data of 460,797 participants (median follow-up of 16.5 years, grouped by birth cohort), this study shows educational inequalities persist and widen across generations. Such disparities influence progression from type 2 diabetes onset to cardiovascular disease and death, and are mainly mediated by socioeconomic, behavioral, and metabolic factors. Targeted prevention and clinical management for people with lower education may mitigate the increasing T2DM burden.

## 1 Introduction

Type 2 diabetes mellitus (T2DM) imposes a substantial and growing burden on global health. Over the past two decades, its global prevalence has increased consistently, although trends in age-standardized disease burden have differed across levels of the Socio-demographic Index (1, 2). Beyond these population-level differences, socioeconomic disadvantage is an important determinant of non-communicable disease and premature mortality (3). Educational attainment is a particularly relevant indicator because it is established relatively early in life and is consistently associated with T2DM risk. A systematic review and meta-analysis, together with subsequent population-based studies, showed that lower educational attainment and socioeconomic position were associated with a higher incidence of T2DM (4–7).

From a life-course perspective, education may influence health through the accumulation of social, behavioral, and biological advantages and disadvantages (8–10). Educational attainment can shape health literacy, employment opportunities, income, residential circumstances, health behaviors, and metabolic risk throughout adulthood. Previous UK Biobank studies have linked educational disadvantage to biological aging, cardiovascular morbidity, and mortality (11), and have reported increasing educational inequalities in cardiovascular disease across successive generations (12). Among participants with established T2DM, broader unfavorable social determinants and lower educational attainment have also been associated with higher risks of complications and mortality (13, 14).

Nevertheless, these studies addressed different components of the disease process separately. Studies of T2DM incidence generally enrolled participants without diabetes and followed them only until disease onset, whereas studies of prognosis began with populations who already had T2DM. Similarly, previous generational studies focused primarily on educational inequalities in mortality or cardiovascular disease (12, 15). Consequently, they did not determine whether the same educational gradient extends from diabetes-free status to incident T2DM and then to subsequent CVD or death within a single prospective framework. This question is particularly relevant in the United Kingdom, where educational opportunities expanded substantially after World War II (9), potentially changing the social composition and health risks of lower educational groups across birth cohorts.

We therefore used the UK Biobank to integrate three questions that had previously been examined separately: educational inequalities in incident T2DM, inequalities in subsequent progression to CVD and death, and differences in these associations across birth cohorts. We further quantified relative and absolute inequalities and assessed the contributions of socioeconomic, behavioral, and metabolic pathway factors. Distinguishing inequalities in disease onset from those in post-diagnosis progression could inform population-level prevention and screening, as well as targeted clinical education and disease management, particularly for less-educated individuals in younger birth cohorts.

## 2 Methods

### 2.1 Study design

This study utilized a prospective cohort design and is reported in accordance with the Strengthening the Reporting of Observational Studies in Epidemiology (STROBE) statement.

### 2.2 Study population

This study utilized data from the UK Biobank, a large-scale prospective cohort of approximately 500,000 participants aged 40–69 years recruited between 2006 and 2010 (Application Number 103736). The UK Biobank study received ethical approval from the North West Multi-center Research Ethics Committee (MREC), and all participants provided informed electronic consent. We included individuals who completed the baseline assessment. Participants were excluded if they had withdrawn consent, had incomplete data on educational attainment, or had any type of diabetes at baseline (see Supplementary Material for diagnostic algorithms). Following these exclusions, 460,797 participants were included in the final analytical sample. The detailed inclusion and exclusion process is presented in Supplementary Figure 1. To account for potential cohort effects, participants were categorized into three birth cohorts based on their year of birth: those born in or before 1949, between 1950 and 1959, and in or after 1960. These cut-offs were selected to represent broad early, transitional, and later phases of post-war educational expansion in the United Kingdom while retaining sufficient participants and outcome events in each cohort, rather than as exact thresholds for any single educational reform. Given the nature of the UK Biobank as a large-scale population-based cohort, no *a priori* sample size calculation was performed. We included all participants meeting the eligibility criteria to maximize statistical power for stratified analyses.

### 2.3 Assessment of educational attainment and other covariates

Educational attainment was derived from the UK Biobank qualifications field, which allowed participants to select more than one qualification. Participants reporting multiple qualifications were classified according to their highest reported qualification. Educational attainment was categorized into four hierarchical groups: college or university degree, high school, middle school, and no listed formal qualification/primary school or less. Participants selecting “None of the above” were included in the lowest education group because this response indicated that none of the listed formal qualifications had been obtained. This operational classification is consistent with previous studies of educational inequalities across birth cohorts (12).

Baseline disease diagnoses were ascertained based on self-reported medical history and ICD-9 and ICD-10 codes (detailed below and Supplementary materials). Covariates were selected *a priori* based on previous epidemiological evidence and a life-course conceptual framework linking educational attainment to T2DM risk (4, 8, 12). Age, sex, ethnicity, and the polygenic risk score (PRS) for T2DM constituted the primary adjustment set because they capture demographic structure and inherited susceptibility to T2DM and were considered unlikely to be consequences of adult educational attainment. Adult socioeconomic factors (Townsend Deprivation Index [TDI], marital status, employment status, and household income), behavioral factors (smoking, alcohol consumption, diet, sleep, and physical activity) (16, 17), and metabolic factors (body mass index [BMI], high-density lipoprotein cholesterol [HDL-C], triglycerides, HbA1c, and systolic blood pressure [SBP]) were considered potential pathway factors that may partly lie downstream of education. These factors were therefore not included in the primary adjustment model but were added separately and sequentially in subsequent models to assess the attenuation of the education–T2DM association. This modeling strategy distinguished the association after basic adjustment from pathway-adjusted associations and reduced the risk of over-adjustment. Detailed definitions and data sources are provided in Supplementary Methods.

### 2.4 Assessment of endpoints

The primary outcome of interest was incident T2DM. Incident cases were identified through longitudinal linkage to national electronic health records, including the Hospital Episode Statistics (HES) for England and Wales and the Scottish Morbidity Record (SMR) for Scotland. This linkage was facilitated by NHS England (formerly NHS Digital) and the NHS Central Register using participants' unique National Health Service (NHS) identification numbers, date of birth, and sex. T2DM was defined according to the International Classification of Diseases codes: E11 for ICD-10 and 250 for ICD-9. All-cause mortality data were obtained from national death registries, as a competing risk event. Cardiovascular disease (CVD) was defined as a composite outcome consisting of ischemic heart disease (IHD), stroke, and heart failure. IHD was identified using ICD-9 codes 410–414 and ICD-10 codes I20–I25, combined with algorithmically defined myocardial infarction events. Stroke was ascertained using ICD-9 codes 430–435 and ICD-10 codes I60–I64, supplemented by algorithmically defined stroke events. Heart failure was identified using ICD-9 code 428 and ICD-10 code I50 from hospital records.

Follow-up duration was calculated from the date of baseline assessment to the date of first diagnosis of T2DM, death, or the administrative censoring date (October 1, 2025), whichever occurred first. Participants lost to follow-up for reasons other than withdrawal of consent were censored at the date of loss.

### 2.5 Statistical analysis

Missing values for covariates were imputed using random forest-based imputation with predictive mean matching, implemented with the missRanger package in R. This approach can accommodate mixed data types and non-linear associations among covariates. To assess the plausibility of the imputation, we compared the distributions of covariates before and after imputation, which showed no substantial distributional changes (Supplementary Figures 2, 3) (18). Baseline characteristics of the study population were summarized according to educational attainment categories. Categorical variables are expressed as counts and percentages, whereas continuous variables are reported as mean ± SD or median [interquartile range (IQR)], with normality assessed via visual inspection of histograms and Q–Q plots. Differences in baseline characteristics across levels of educational attainments were evaluated using one-way ANOVA, the Kruskal–Wallis test, or the Chi-square test, as appropriate.

All analyses described below were conducted in the overall study population and stratified by birth cohort. Given that death acts as a competing risk, we employed Fine-Gray proportional subdistribution hazards models to estimate subdistribution hazard ratios (sHRs) and 95% confidence intervals (CIs) for incident T2DM. The sHRs quantify the association between educational attainment and the cumulative incidence of T2DM in the presence of the competing risk of death. As supplementary analyses, we fitted cause-specific Cox models, in which death before T2DM was treated as censoring. Proportional hazards assumptions for the Cox models were assessed using Schoenfeld residuals. Numbers at risk were additionally provided at prespecified follow-up times. The basic covariates included age, sex, ethnicity, and a polygenic risk score (PRS) for T2DM. To formally test whether the association between educational attainment and incident T2DM differed across birth cohorts, we fitted pooled Fine-Gray models with and without education-by-birth-cohort interaction terms and compared them using likelihood-ratio tests. The interaction test jointly evaluated all education-by-birth-cohort interaction terms. To quantify the magnitude of inequality, the Kunst–Mackenbach Relative Index of Inequality (RII) was calculated (19, 20). Educational categories were ranked from the highest to the lowest socioeconomic position. A continuous metric (range 0–1) was assigned to each category based on the midpoint of its cumulative share of the total population. This derived variable was entered into the regression model as a continuous covariate. The resulting HR represents the RII, which compares the risk of the hypothetically lowest educational group (value = 1) vs. the highest (value = 0). In addition to RII, we quantified absolute educational inequalities in incident T2DM. The slope index of inequality (SII) was estimated to represent the absolute difference in risk across the full educational hierarchy. We also calculated directly standardized 10-year risks by educational attainment using the distribution of age group, sex, and ethnicity in the corresponding analytical population. Standardized risk differences were calculated by comparing participants with primary school education or less with those with college education. Excess event numbers were estimated by multiplying the standardized risk difference by the number of participants in each analytical group.

We applied separate and cumulative adjustments for socioeconomic, behavioral, and metabolic factors to quantify the extent to which these potential pathway factors explained the association between educational attainment and incident T2DM. The proportion mediated by each domain (individually and jointly) was calculated based on the change in the log-transformed HRs between the base and adjusted models, using the formula (21):


$$\begin{array}{l} \text{Mediation proportion }=\;\frac{\ln\left(HR_{base}\right)-\ln\left(HR_{adjusted}\right)}{\ln\left(HR_{base}\right)}\;\times \;100\% \end{array}$$

where *HR*_*base*_ represents the hazard ratio for the association between educational attainment and incident T2DM in the primary model, and *HR*_*adjusted*_ is the hazard ratio after further adjustment for the specific mediators. To ensure the statistical robustness of these estimates, we employed a non-parametric bootstrap procedure with 1,000 resamples. The 95% CIs for the proportion mediated were derived using the percentile method (based on the 2.5th and 97.5th percentiles of the bootstrap distribution).

To comprehensively characterize the impact of education level on the entire disease trajectory, spanning from the onset of T2DM to subsequent adverse sequelae including CVD and mortality, we employed a multi-state competing risks model. Time since baseline was used as the time scale. Separate models were constructed for the CVD and mortality pathways. For the CVD pathway, transitions included baseline to T2DM, baseline to CVD, and T2DM to CVD. Participants who developed CVD before T2DM contributed to the baseline-to-CVD transition and were not included in the T2DM-to-CVD risk set. For the mortality pathway, transitions included baseline to T2DM, baseline to death, and T2DM to death. Same-day T2DM/CVD and same-day T2DM/death events were retained by assigning T2DM as occurring first and shifting the downstream event time to avoid zero-length intervals. Transition-specific risk-set sizes and event counts were calculated from the counting-process data. Analyses were implemented using the mstate package in R (22).

Additionally, a series of sensitivity analyses were performed to assess the robustness of the findings. First, to address potential confounding arising from the markedly different baseline ages across birth cohorts, we performed an attained-age time-scale analysis with left truncation. Age at baseline was specified as the entry time, and age at the end of follow-up was specified as the exit time, thereby accounting for delayed entry. For incident T2DM, cause-specific Cox models were fitted, with death treated as a censoring event, and the same covariates were included as in the primary models.

Second, to further harmonize the age range and survival experience across birth cohorts, we retained the reconstructed cohort analysis in which follow-up was initiated at age 30. Participants who died before age 69, corresponding to the upper age limit of UK Biobank recruitment, were excluded. This analysis was intended to reduce bias arising from differential survival before recruitment across birth cohorts. Third, given the differences in educational distribution and T2DM risk by sex, all relevant analyses were stratified by sex.

All statistical tests were two-sided, and a *P* value < 0.05 was considered statistically significant. All analyses were performed using R statistical software (version 4.4.3; The R Foundation for Statistical Computing, Vienna, Austria).

## 3 Results

### 3.1 Baseline characteristics

Among the 501,950 participants initially recruited, 158 withdrew consent, 10,104 had missing data on educational attainment, and 30,891 had prevalent diabetes at baseline (Supplementary Figure 1). The final analytical cohort included 460,797 participants free of diabetes at baseline, with a mean age of 56.34 (SD, 8.10) years; 205,485 (44.6%) were male. During 7,264,116 person-years of follow-up, 19,931 participants developed incident T2DM. The proportion excluded because of prevalent diabetes was higher among participants with primary school education or less (9.96%) than among those with college education (5.20%), high school education (5.94%), or middle school education (5.94%). Baseline characteristics of the final analytical cohort are presented in Table 1 and Supplementary Tables 1–3.

**Table 1:** Baseline characteristics of the final analytical cohort according to educational attainment.

Variables	Level	Overall	College education	High school	Middle school	Primary school or less	*P*	SMD
		460,797	224,686	81,170	78,225	76,716		
Age, years, mean (SD)		56.34 (8.10)	54.81 (8.02)	56.84 (7.91)	55.74 (8.21)	60.89 (6.53)	< 0.001	0.432
Sex, *n* (%)	Female	255,312 (55.4)	113,403 (50.5)	49,321 (60.8)	50,109 (64.1)	42,479 (55.4)	< 0.001	0.157
	Male	205,485 (44.6)	111,283 (49.5)	31,849 (39.2)	28,116 (35.9)	34,237 (44.6)		
Ethnicity, *n* (%)	Non-white	18,055 (4.0)	9,831 (4.4)	2,857 (3.6)	2,870 (3.7)	2,497 (3.3)	< 0.001	0.031
	White	437,419 (96.0)	211,668 (95.6)	77,455 (96.4)	74,794 (96.3)	73,502 (96.7)		
PRS of T2DM, mean (SD)		−0.05 (0.99)	−0.07 (0.99)	−0.06 (0.98)	−0.02 (0.98)	−0.01 (0.98)	< 0.001	0.048
Socioeconomic factors								
Townsend deprivation index, median [IQR]		−2.20 [−3.68, 0.41]	−2.30 [−3.75, 0.20]	−2.53 [−3.85, −0.33]	−2.27 [−3.67, 0.21]	−1.27 [−3.12, 1.96]	< 0.001	0.219
Household income, *n* (%)	< 18,000	86,715 (21.8)	26,750 (13.1)	12,506 (17.8)	17,102 (25.9)	30,357 (53.4)	< 0.001	0.664
	18,000–30,999	100,407 (25.3)	44,224 (21.7)	19,631 (27.9)	20,443 (31.0)	16,109 (28.3)		
	31,000–51,999	105,185 (26.5)	58,997 (28.9)	20,551 (29.2)	18,071 (27.4)	7,566 (13.3)		
	52,000–100,000	82,844 (20.8)	56,861 (27.8)	14,541 (20.7)	8,970 (13.6)	2,472 (4.3)		
	>100,000	22,212 (5.6)	17,410 (8.5)	3,069 (4.4)	1,339 (2.0)	394 (0.7)		
Marriage, *n* (%)	Married/co-habiting	335,990 (89.4)	166,249 (89.6)	59,778 (89.8)	56,975 (88.2)	52,988 (89.6)	< 0.001	0.024
	Not married/co-habiting	39,839 (10.6)	19,270 (10.4)	6,826 (10.2)	7,603 (11.8)	6,140 (10.4)		
Employment status, *n* (%)	Employed	272,194 (59.6)	154,331 (69.2)	46,166 (57.3)	45,301 (58.3)	26,396 (34.8)	< 0.001	0.382
	Unemployed	35,678 (7.8)	13,302 (6.0)	5,455 (6.8)	7,409 (9.5)	9,512 (12.6)		
	Retired	149,168 (32.6)	55,476 (24.9)	28,918 (35.9)	24,934 (32.1)	39,840 (52.6)		
Behavioral factors								
Alcohol intake, *n* (%)	Never	18,769 (4.9)	7,270 (3.8)	3,295 (4.9)	3,202 (5.0)	5,002 (8.3)	< 0.001	0.142
	Previous	15,437 (4.0)	6,365 (3.3)	2,472 (3.6)	2,574 (4.0)	4,026 (6.7)		
	Safe	184,339 (47.8)	92,541 (47.8)	33,847 (49.9)	31,487 (49.3)	26,464 (44.0)		
	Dangerous	166,906 (43.3)	87,395 (45.1)	28,255 (41.6)	26,637 (41.7)	24,619 (41.0)		
Dietary habits, *n* (%)	Healthy	214,632 (59.7)	108,450 (61.1)	39,266 (61.1)	34,875 (56.8)	32,041 (56.7)	< 0.001	0.059
	Unhealthy	145,141 (40.3)	69,157 (38.9)	25,007 (38.9)	26,495 (43.2)	24,482 (43.3)		
Sleep habits, *n* (%)	Unhealthy	32,000 (7.0)	12,086 (5.4)	5,113 (6.3)	5,801 (7.5)	9,000 (11.9)	< 0.001	0.152
	Medium	113,590 (24.8)	52,500 (23.4)	19,621 (24.3)	20,058 (25.8)	21,411 (28.3)		
	Healthy	312,628 (68.2)	159,406 (71.2)	56,103 (69.4)	51,957 (66.8)	45,162 (59.8)		
Physical activity, *n* (%)	Low	64,820 (18.0)	33,674 (18.0)	11,640 (18.1)	11,059 (18.6)	8,447 (17.5)	< 0.001	0.074
	Moderate	146,534 (40.7)	78,878 (42.1)	26,567 (41.2)	23,564 (39.7)	17,525 (36.2)		
	High	148,338 (41.2)	75,012 (40.0)	26,241 (40.7)	24,711 (41.6)	22,374 (46.3)		
Smoking status, *n* (%)	Never	254,201 (55.4)	131,076 (58.5)	46,063 (56.9)	42,671 (54.7)	34,391 (45.2)	< 0.001	0.159
	Previous	156,882 (34.2)	72,974 (32.6)	27,505 (34.0)	26,224 (33.6)	30,179 (39.7)		
	Current	48,117 (10.5)	20,101 (9.0)	7,404 (9.1)	9,110 (11.7)	11,502 (15.1)		
Metabolic factors								
SBP, mmHg, [mean (SD)]		137.56 (18.69)	135.79 (18.26)	137.61 (18.66)	137.62 (18.59)	142.68 (19.15)	< 0.001	0.184
BMI, kg/m^2^, [mean (SD)]		27.16 (4.60)	26.82 (4.50)	27.11 (4.58)	27.33 (4.68)	28.06 (4.70)	< 0.001	0.142
Waist, cm, [mean (SD)]		89.46 (13.00)	89.03 (12.85)	88.84 (13.03)	88.83 (13.08)	92.01 (13.06)	< 0.001	0.125
HbA1c, %, [mean (SD)]		5.35 (0.34)	5.32 (0.34)	5.36 (0.34)	5.35 (0.34)	5.44 (0.34)	< 0.001	0.178
FPG, mmol/L, [mean (SD)]		5.00 (0.52)	4.99 (0.52)	5.02 (0.52)	4.99 (0.51)	5.01 (0.54)	0.03	0.034
TC, mmol/L, [mean (SD)]		5.76 (1.11)	5.73 (1.09)	5.80 (1.11)	5.81 (1.10)	5.76 (1.18)	< 0.001	0.043
HDL-C, mmol/L, [mean (SD)]		1.46 (0.38)	1.46 (0.38)	1.49 (0.39)	1.48 (0.38)	1.43 (0.37)	< 0.001	0.091
LDL, mmol/L, [mean (SD)]		3.60 (0.85)	3.59 (0.83)	3.62 (0.85)	3.63 (0.85)	3.61 (0.90)	< 0.001	0.03
TG, mmol/L, [median (IQR)]		1.46 [1.03, 2.11]	1.42 [1.00, 2.06]	1.45 [1.03, 2.09]	1.46 [1.04, 2.10]	1.60 [1.15, 2.26]	< 0.001	0.086
Comorbidities								
CVD, *n* (%)		28,901 (6.3)	10,799 (4.8)	4,759 (5.9)	4,266 (5.5)	9,077 (11.8)	< 0.001	0.132
CAD, *n* (%)		21,681 (4.7)	7,908 (3.5)	3,520 (4.3)	3,102 (4.0)	7,151 (9.3)	< 0.001	0.123
Stroke, *n* (%)		8,095 (1.8)	3,078 (1.4)	1,378 (1.7)	1,293 (1.7)	2,346 (3.1)	< 0.001	0.058
HF, *n* (%)		1,933 (0.4)	707 (0.3)	317 (0.4)	256 (0.3)	653 (0.9)	< 0.001	0.037

This presents the data distribution prior to missing data imputation. SD, standard deviation; SMD, standardized mean difference; PRS, polygenic risk score; T2DM, Type 2 diabetes mellitus; IQR, interquartile range; BMI, body mass index; HbA1c, hemoglobin A1c (Glycated hemoglobin); SBP, systolic blood pressure; HDL-C, high-density lipoprotein cholesterol; LDL-C, low-density lipoprotein cholesterol; TG, triglycerides; CVD, cardiovascular disease; CAD, coronary artery disease; HF, heart failure.

In the final analytical cohort, 224,686 participants (48.8%) had a college education, 81,170 (17.6%) had completed high school, 78,225 (17.0%) had completed middle school, and 76,716 (16.6%) had primary school education or less. The validity of this educational stratification was supported by cognitive test scores (Supplementary Figure 4). Participants with higher educational attainment had a more favorable socioeconomic profile, including lower Townsend deprivation index, higher household income, and lower unemployment. They also had a lower burden of cardiometabolic risk factors, with lower BMI, waist circumference, HbA1c, fasting plasma glucose, triglycerides, and systolic blood pressure, and higher HDL-C. Smoking, poor sleep, and unhealthy dietary habits were more common among participants with lower educational attainment, whereas excessive alcohol consumption and insufficient physical activity were more frequent among those with higher educational attainment.

Educational distributions differed substantially across birth cohorts. The proportion of participants with primary school education or less declined from 26.0% in the pre-1949 cohort to 11.3% in the 1950–1959 cohort and 4.8% in the post-1960 cohort (Supplementary Table 4 and Figure 1). The corresponding numbers of incident T2DM events and person-years were 11,599 events over 3,244,834 person-years in the pre-1949 cohort, 5,775 events over 2,402,989 person-years in the 1950–1959 cohort, and 2,557 events over 1,616,293 person-years in the post-1960 cohort. Socioeconomic, behavioral, and metabolic differences by educational attainment were observed across all birth cohorts.

**Figure 1:**
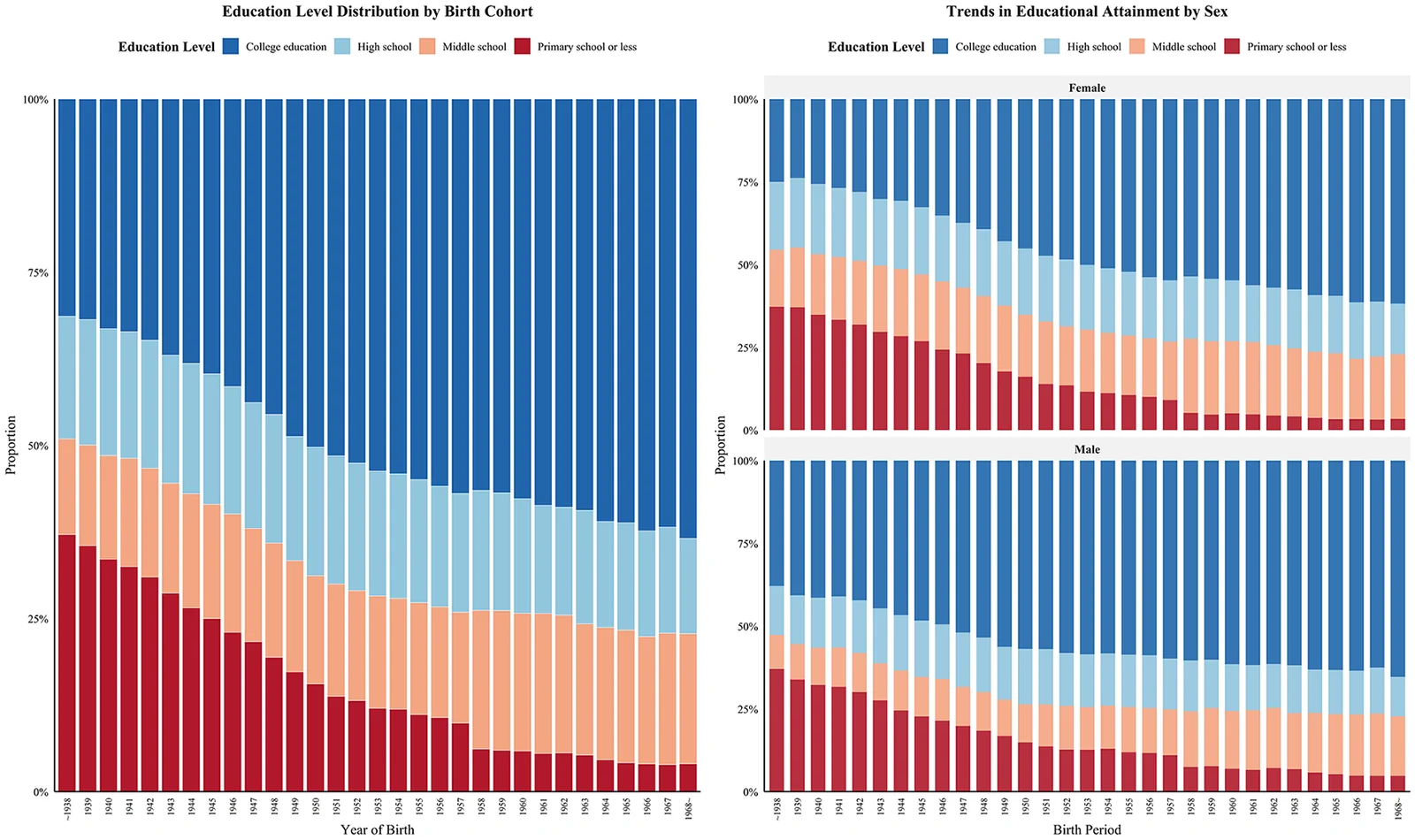
Distribution of educational attainment across birth years among study participants.

### 3.2 Education level and T2DM risk

During a median follow-up of 16.5 years, we documented 19,931 incident T2DM cases after excluding participants with diabetes at baseline. An inverse association between educational attainment and the risk of incident T2DM was observed in the total population and across all birth cohorts (*P* < 0.001 for Gray's test) (Figure 2 and Supplementary Tables 5, 6). The association persisted before and after adjustment for confounders (HR and 95% CI: 2.32 [2.24–2.40] and 1.92 [1.85–1.99], after adjusting for age, sex, ethnicity, and T2DM PRS) but was substantially attenuated upon further accounting for pathway mediators (HR and 95% CI: 1.22 [1.17–1.27], after adjusting for socioeconomic, behavioral, and metabolic factors). Despite this attenuation, the risk estimates remained statistically significant for all groups except the post-1960 birth cohort (Figure 3 and Supplementary Tables 7, 8). Furthermore, the significance of the effect persisted when adjusting for each category of covariates individually. When stratified by birth cohort, the risk of T2DM associated with lower education appeared to strengthen over time. Compared with participants with a college education, the multivariable-adjusted HRs for those with primary school education or less increased from 1.78 (1.70–1.86) in the pre-1949 birth cohort to 2.22 (2.07–2.37) in the 1950–1959 cohort, and further to 2.26 (1.98–2.58) in the post-1960 cohort. Results stratified by sex were consistent with the findings in the overall population. In pooled Fine-Gray models, the education-by-birth-cohort interaction was statistically significant in the overall population in the basic adjusted model (*P* for interaction < 0.001), indicating that the association between educational attainment and incident T2DM differed across birth cohorts (Supplementary Tables 9, 10). The interaction was attenuated after further adjustment for socioeconomic, behavioral, and metabolic factors, particularly in the fully adjusted model. Full interaction *P* values are presented in Supplementary Tables 9, 10. Supplementary cause-specific Cox analyses yielded generally consistent results (Supplementary Tables 11, 12). In the basic adjustment model, compared with participants with a college education, the cause-specific HR for incident T2DM among those with primary school education or less was 1.96 (95% CI, 1.89–2.03) in the overall population. The corresponding HRs were 1.82 (1.74–1.90), 2.26 (2.11–2.41), and 2.33 (2.05–2.65) in the pre-1949, 1950–1959, and post-1960 cohorts, respectively. No evidence of violation of the proportional hazards assumption was observed for educational attainment in Model 1.

**Figure 2:**
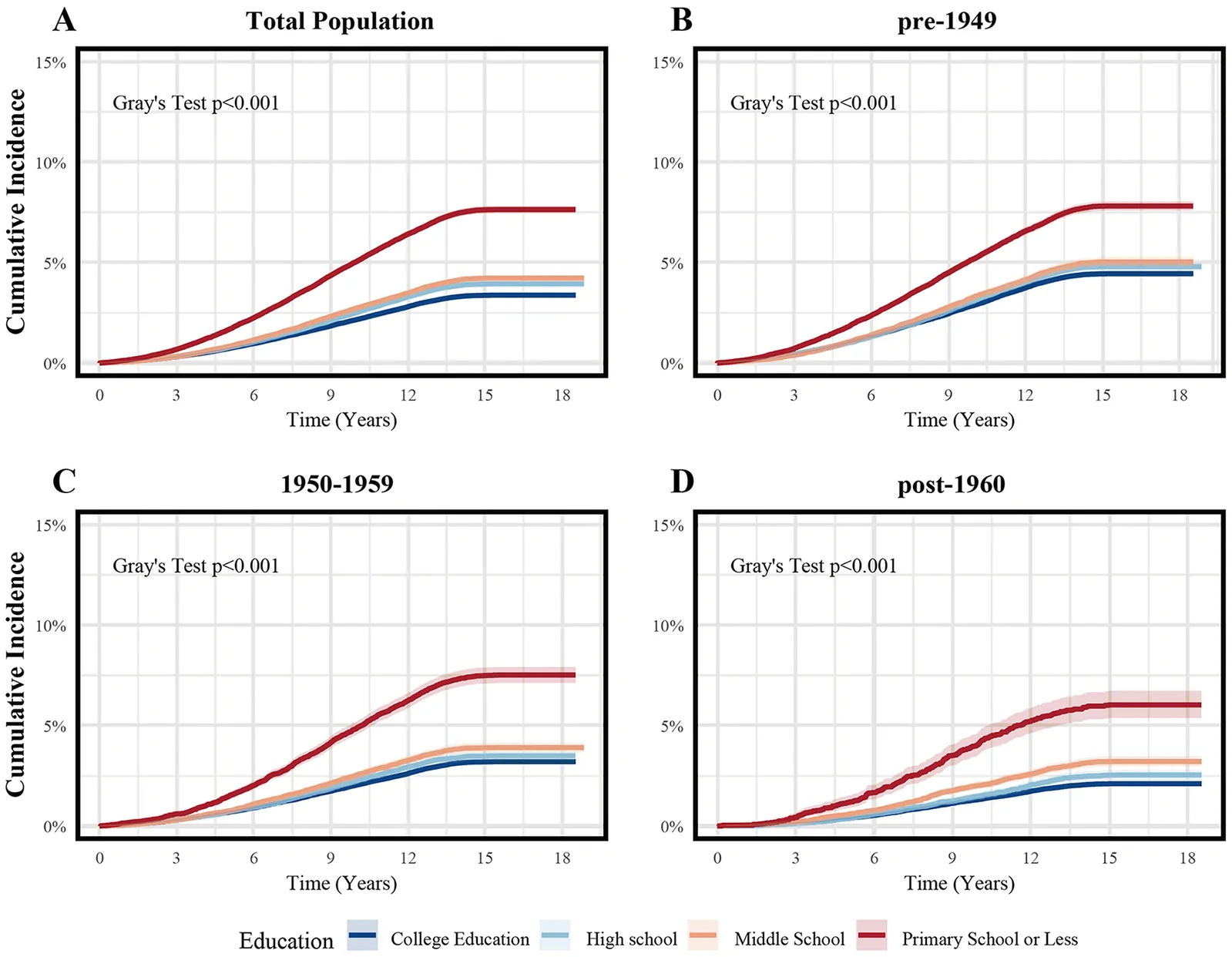
Cumulative incidence functions (CIF) for type 2 diabetes by educational attainment in the overall population and stratified by birth cohorts. **(A)** Cumulative incidence of type 2 diabetes in the total study population. **(B–D)** Cumulative incidence stratified by birth cohorts: pre-1949 **(B)**, 1950–1959 **(C)**, and post-1960 **(D)**.

**Figure 3:**
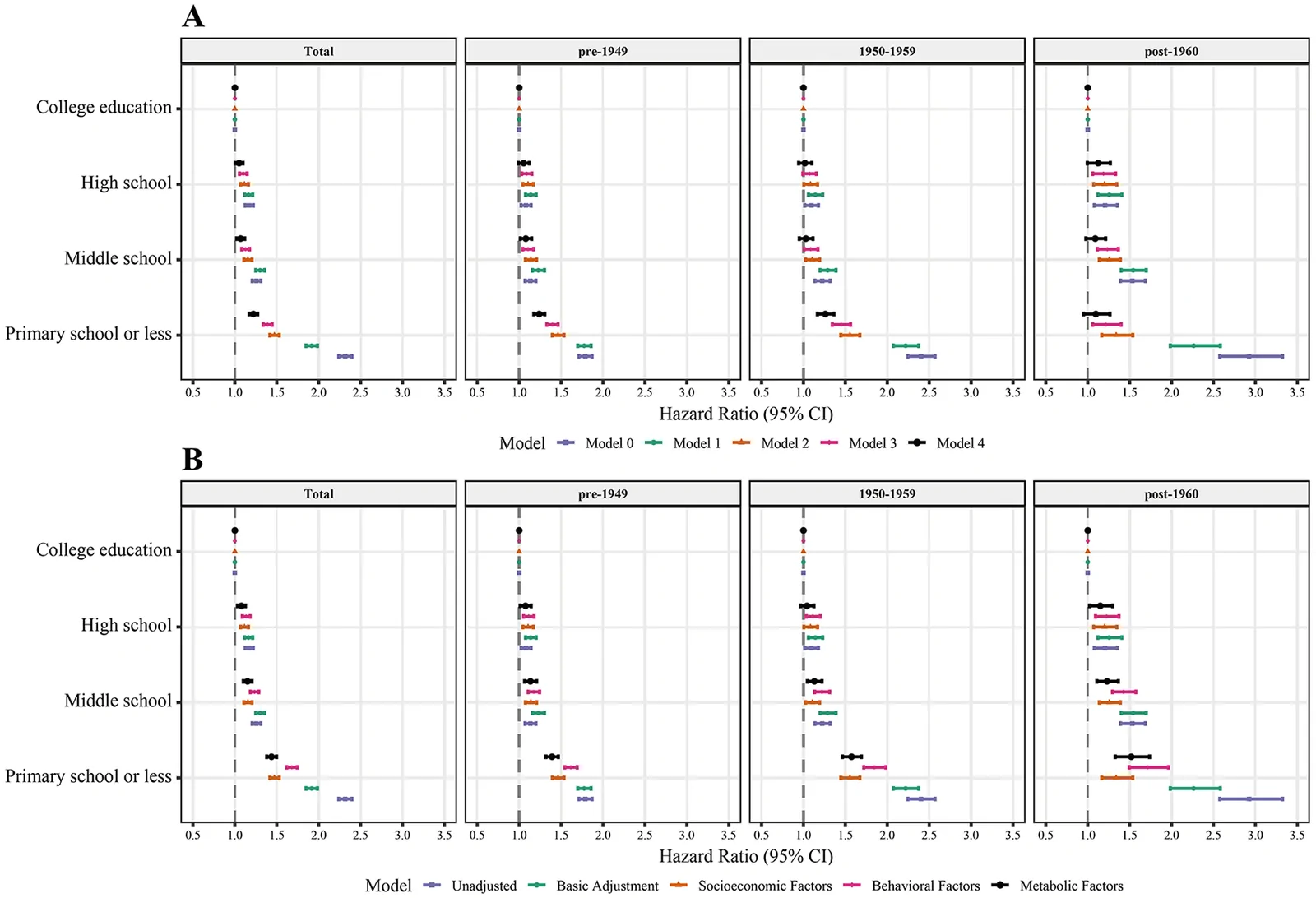
Associations between educational attainment and risk of incident type 2 diabetes. Covariates were categorized into three groups: Socioeconomic (Townsend deprivation index, household income, marital and employment status); Behavioral (alcohol, diet, sleep, physical activity, smoking); and Metabolic (BMI, HbA1c, HDL-C, SBP, triglycerides). **(A)** Sequential adjustment: Model 0: unadjusted. Model 1: adjusted for age, sex, ethnicity, and T2DM PRS. Model 2: Model 1 + Socioeconomic. Model 3: Model 2 + Behavioral. Model 4: Model 3 + Metabolic. **(B)** Separate adjustment: Each covariate group was added individually to Model 1. Sex was excluded from models in sex-stratified analyses.

In an additional sensitivity analysis using attained age as the underlying time scale with delayed entry at baseline age (Supplementary Table 15), the educational gradient in incident T2DM remained consistent. Compared with participants with a college education, the adjusted hazard ratios for primary education or less were 2.06 (95% CI, 1.99–2.13) in the overall population, 1.86 (1.78–1.94) in the pre-1949 cohort, 2.28 (2.13–2.44) in the 1950–1959 cohort, and 2.35 (2.07–2.68) in the post-1960 cohort. These findings were consistent with the primary analysis. In the reconstructed cohort with follow-up starting at age 30, despite a marked increase in the risk of T2DM before age 60 from the pre-1949 to the post-1960 birth cohorts, the trend of increasing risk with decreasing educational attainment persisted in the overall population and across all birth cohorts (Figure 4A and Supplementary Table 13). Compared with individuals with a college education, the adjusted HRs for those with primary school education or less increased from 1.30 (1.21–1.40) in the pre-1949 birth cohort to 1.83 (1.71–1.97) in the 1950–1959 cohort, and further to 2.32 (2.10–2.56) in the post-1960 cohort (Figure 4B and Supplementary Table 14). Likewise, the results were consistent across sex subgroups.

**Figure 4:**
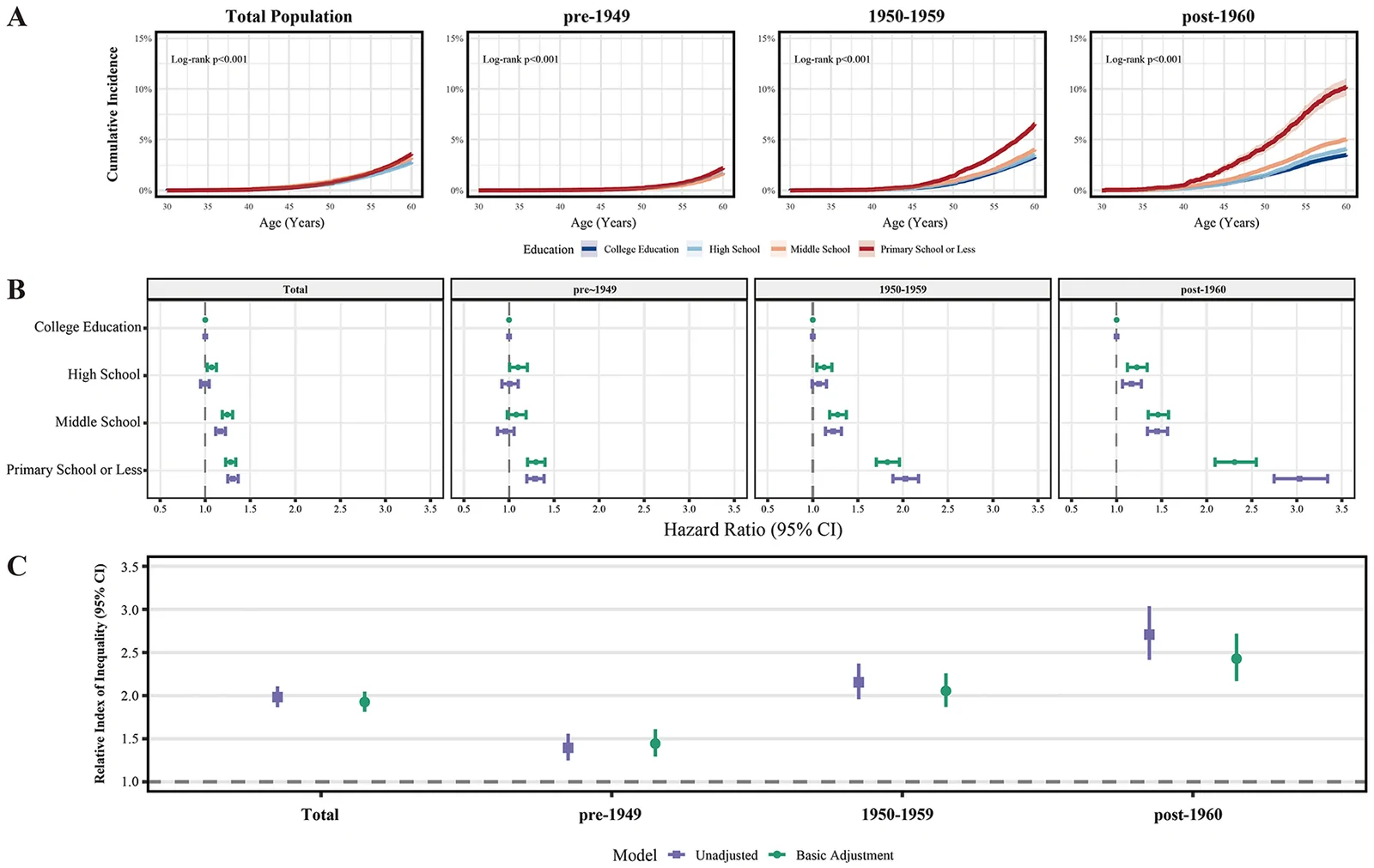
Sensitivity analyses of educational inequalities in type 2 diabetes risk using a reconstructed cohort with follow-up commencing at age 30. **(A)** Cumulative incidence of type 2 diabetes starting from age 30 in the overall population and stratified by birth cohorts. **(B)** Associations between educational attainment and type 2 diabetes risk in the reconstructed cohort. Hazard ratios (HRs) compare lower educational levels. **(C)** Relative Index of Inequality (RII) quantifying the magnitude of education-related disparities in the overall population and across successive birth cohorts. The basic adjustment model was adjusted for age, sex, ethnicity, and polygenic risk score (PRS) for type 2 diabetes.

### 3.3 Educational inequalities in T2DM risk

The RII for education in relation to T2DM risk was progressively attenuated after sequential adjustment for confounders and mediators. However, it remained statistically significant in the overall population and across all birth cohorts (Figure 5 and Supplementary Tables 16, 17). In the model adjusted for confounders alone, the RII increased from 2.31 (2.16–2.47) in the pre-1949 cohort to 2.53 (2.29–2.79) in the 1950–1959 cohort, and 2.53 (2.18–2.93) in the post-1960 cohort, indicating an upward trend. We further quantified absolute educational inequalities in 10-year risk of incident T2DM. After direct standardization by age group, sex, and ethnicity, the 10-year risk was 2.20% among participants with college education and 4.78% among those with primary school education or less in the overall population, corresponding to a standardized risk difference of 2.57 percentage points and 11,862 excess T2DM events. Across birth cohorts, the standardized risk differences were 2.43, 2.77, and 2.49 percentage points in the pre-1949, 1950–1959, and post-1960 cohorts, respectively. The corresponding SII values were 3.03, 3.34, 2.59, and 1.83 percentage points in the overall population and the three birth cohorts, respectively (Supplementary Table 18).

**Figure 5:**
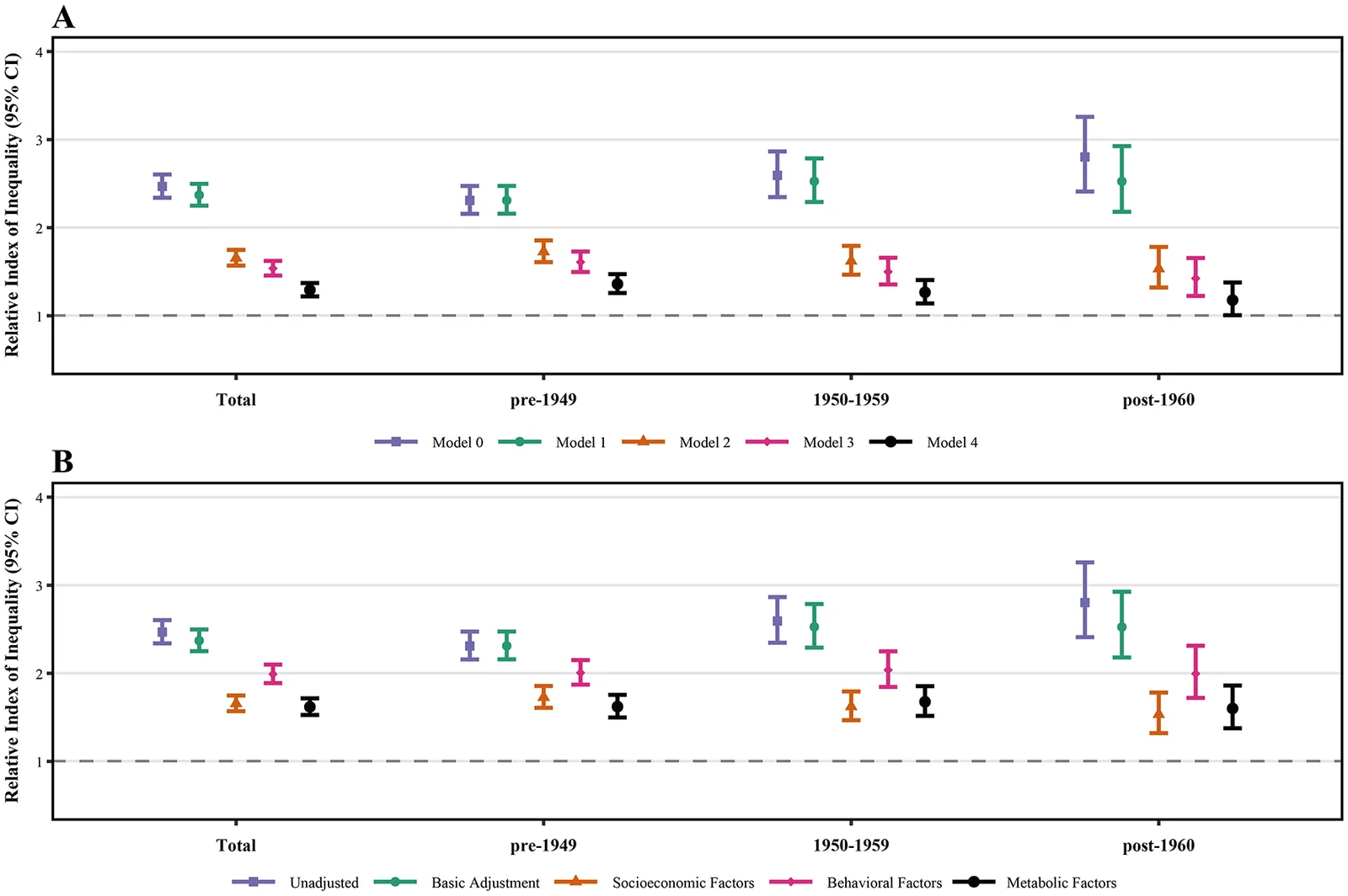
Education-related Relative Index of Inequality (RII) for the risk of incident type 2 diabetes. Covariates were categorized into three groups: Socioeconomic (Townsend deprivation index, household income, marital and employment status); Behavioral (alcohol, diet, sleep, physical activity, smoking); and Metabolic (BMI, HbA1c, HDL-C, SBP, triglycerides). **(A)** Sequential adjustment: Model 0: unadjusted. Model 1: adjusted for age, sex, ethnicity, and T2DM PRS. Model 2: Model 1 + Socioeconomic. Model 3: Model 2 + Behavioral. Model 4: Model 3 + Metabolic. **(B)** Separate adjustment: Each covariate group was added individually to Model 1. Sex was excluded from models in sex-stratified analyses.

In the reconstructed cohort with follow-up starting at age 30, the magnitude of educational inequality in T2DM risk by age 60 increased significantly across successive birth cohorts. The relative inequality estimate rose from 1.45 (1.30–1.61) in the pre-1949 cohort to 2.06 (1.87–2.26) in the 1950–1959 cohort, and further to 2.43 (2.17–2.72) in the post-1960 cohort (Figure 4C and Supplementary Table 19). Significant education-related inequalities and the increasing magnitude of these disparities were consistently observed across sex subgroups.

### 3.4 Educational attainment and progression to CVD and mortality

Results from multi-state competing risk models indicated that lower educational attainment was significantly associated with an increased risk of progression from T2DM to CVD and from T2DM to mortality in the overall population and across all birth cohorts. Compared with the college education group, the risk with lower education increased across successive generations. For the transition from T2DM to CVD, the adjusted HRs for individuals with primary education or less rose from 1.25 (1.21–1.28) in the pre-1949 cohort to 1.52 (1.44–1.60) in the 1950–1959 cohort and 2.14 (1.91–2.39) in the post-1960 cohort (Table 2). Similarly, for the transition from T2DM to mortality, the adjusted HRs increased from 1.45 (1.41–1.50) to 1.86 (1.74–1.98) and 1.99 (1.69–2.34) across the respective cohorts (Supplementary Table 20). The transition-specific risk-set sizes and event counts are provided in Supplementary Table 22.

**Table 2:** Hazard ratios for transitions between health, T2DM, and CVD according to education level in a multi-state model.

Transition	Period	Unadjusted			Basic adjusted		
		Education	HR (95% CI)	*P* value	Education	HR (95% CI)	*P* value
Baseline to T2DM	Total	College education	Ref	–	College education	Ref	–
		High school	1.18 (1.13–1.24)	< 0.001	High school	1.17 (1.12–1.23)	< 0.001
		Middle school	1.24 (1.19–1.29)	< 0.001	Middle school	1.28 (1.23–1.34)	< 0.001
		Primary school or less	2.40 (2.32–2.49)	< 0.001	Primary school or less	1.96 (1.88–2.03)	< 0.001
	Pre-1949	College education	Ref	–	College education	Ref	–
		High school	1.09 (1.03–1.16)	0.003	High school	1.15 (1.09–1.23)	< 0.001
		Middle school	1.11 (1.04–1.18)	0.001	Middle school	1.21 (1.14–1.29)	< 0.001
		Primary school or less	1.83 (1.75–1.92)	< 0.001	Primary school or less	1.81 (1.73–1.90)	< 0.001
	1950–1959	College education	Ref	–	College education	Ref	–
		High school	1.09 (1.01–1.18)	0.029	High school	1.14 (1.05–1.23)	0.001
		Middle school	1.21 (1.12–1.31)	< 0.001	Middle school	1.27 (1.18–1.38)	< 0.001
		Primary school or less	2.44 (2.27–2.62)	< 0.001	Primary school or less	2.25 (2.09–2.41)	< 0.001
	Post-1960	College education	Ref	–	College education	Ref	–
		High school	1.22 (1.08–1.37)	< 0.001	High school	1.27 (1.13–1.42)	< 0.001
		Middle school	1.50 (1.35–1.65)	< 0.001	Middle school	1.50 (1.35–1.65)	< 0.001
		Primary school or less	2.96 (2.59–3.38)	< 0.001	Primary school or less	2.33 (2.04–2.66)	< 0.001
T2DM to CVD	Total	College education	ref	–	College education	Ref	–
		High school	1.11 (1.08–1.14)	< 0.001	High school	1.05 (1.02–1.08)	< 0.001
		Middle school	1.07 (1.04–1.10)	< 0.001	Middle school	1.11 (1.08–1.14)	< 0.001
		Primary school or less	1.83 (1.79–1.88)	< 0.001	Primary school or less	1.33 (1.30–1.37)	< 0.001
	Pre-1949	College education	Ref	–	College education	Ref	–
		High school	0.98 (0.95–1.01)	0.223	High school	1.03 (1.00–1.06)	0.096
		Middle school	0.95 (0.92–0.98)	0.005	Middle school	1.04 (1.01–1.08)	0.02
		Primary school or less	1.26 (1.22–1.29)	< 0.001	Primary school or less	1.25 (1.21–1.28)	< 0.001
	1950–1959	College education	Ref	–	College education	Ref	–
		High school	0.98 (0.93–1.04)	0.502	High school	1.04 (0.98–1.09)	0.17
		Middle school	1.05 (1.00–1.11)	0.049	Middle school	1.16 (1.10–1.23)	< 0.001
		Primary school or less	1.56 (1.48–1.65)	< 0.001	Primary school or less	1.52 (1.44–1.60)	< 0.001
	Post-1960	College education	Ref	–	College education	ref	–
		High school	1.03 (0.94–1.14)	0.511	High school	1.09 (0.99–1.20)	0.085
		Middle school	1.27 (1.17–1.38)	< 0.001	Middle school	1.30 (1.20–1.41)	< 0.001
		Primary school or less	2.39 (2.14–2.67)	< 0.001	Primary school or less	2.14 (1.91–2.39)	< 0.001
Baseline to CVD	Total	College education	Ref	–	College education	Ref	–
		High school	0.91 (0.81–1.01)	0.086	High school	0.88 (0.79–0.99)	0.032
		Middle school	0.98 (0.87–1.09)	0.672	Middle school	0.99 (0.89–1.11)	0.869
		Primary school or less	1.15 (1.05–1.26)	0.002	Primary school or less	1.07 (0.97–1.17)	0.171
	Pre-1949	College education	Ref	–	College education	Ref	–
		High school	0.90 (0.78–1.04)	0.15	High school	0.89 (0.77–1.03)	0.121
		Middle school	0.96 (0.82–1.11)	0.547	Middle school	0.95 (0.82–1.11)	0.535
		Primary school or less	1.03 (0.92–1.15)	0.634	Primary school or less	1.02 (0.91–1.14)	0.789
	1950–1959	College education	Ref	–	College Education	Ref	–
		High school	0.79 (0.63–0.99)	0.041	High school	0.82 (0.65–1.02)	0.077
		Middle school	0.94 (0.76–1.16)	0.566	Middle school	0.97 (0.79–1.20)	0.797
		Primary school or less	1.18 (0.99–1.41)	0.07	Primary school or less	1.20 (1.00–1.43)	0.051
	Post-1960	College education	Ref	–	College education	Ref	–
		High school	0.94 (0.66–1.34)	0.742	High school	0.93 (0.66–1.33)	0.706
		Middle school	1.11 (0.83–1.47)	0.486	Middle school	1.15 (0.87–1.53)	0.337
		Primary school or less	1.09 (0.75–1.58)	0.645	Primary school or less	1.12 (0.77–1.62)	0.557

The Basic Adjustment model was adjusted for age, sex, ethnicity, and polygenic risk score for type 2 diabetes. T2DM, Type 2 diabetes mellitus; HR, hazard ratio; CI, confidence interval.

In the reconstructed cohort, the adjusted HRs for progression from T2DM to CVD before age 60 for individuals with primary education or less, compared with those with a college education, rose from 1.72 (1.66–1.79) in the pre-1949 cohort to 2.08 (1.96–2.20) in the 1950–1959 cohort and 2.52 (2.28–2.78) in the post-1960 cohort (Supplementary Table 21).

### 3.5 Potential pathway factors linking educational attainment to T2DM risk

In the overall population, socioeconomic, behavioral, and metabolic factors collectively explained 70.5% (65.0%−76.0%) of the educational inequalities in T2DM risk, with proportions of 76.9% (70.2%-84.2%) in women and 62.3% (55.7%-70.7%) in men. Stratification by birth cohort revealed an increasing trend in this proportion: rising from 64.1% (57.5%−72.4%) in the pre-1949 cohort to 75.5% (66.6%−85.0%) in the 1950–1959 cohort, and reaching 81.8% (68.2%−98.7%) in the post-1960 cohort (Figure 6A and Supplementary Table 23). Specifically, in the overall population, the proportions of educational inequalities mediated by socioeconomic, behavioral, and metabolic factors were 42.2% (39.2%−45.2%), 20.5% (18.9%−22.2%), and 42.6% (38.2%−47.3%), respectively. The mediated proportions for all three categories exhibited an upward trend across successive birth cohorts, which was most pronounced for socioeconomic status.

**Figure 6:**
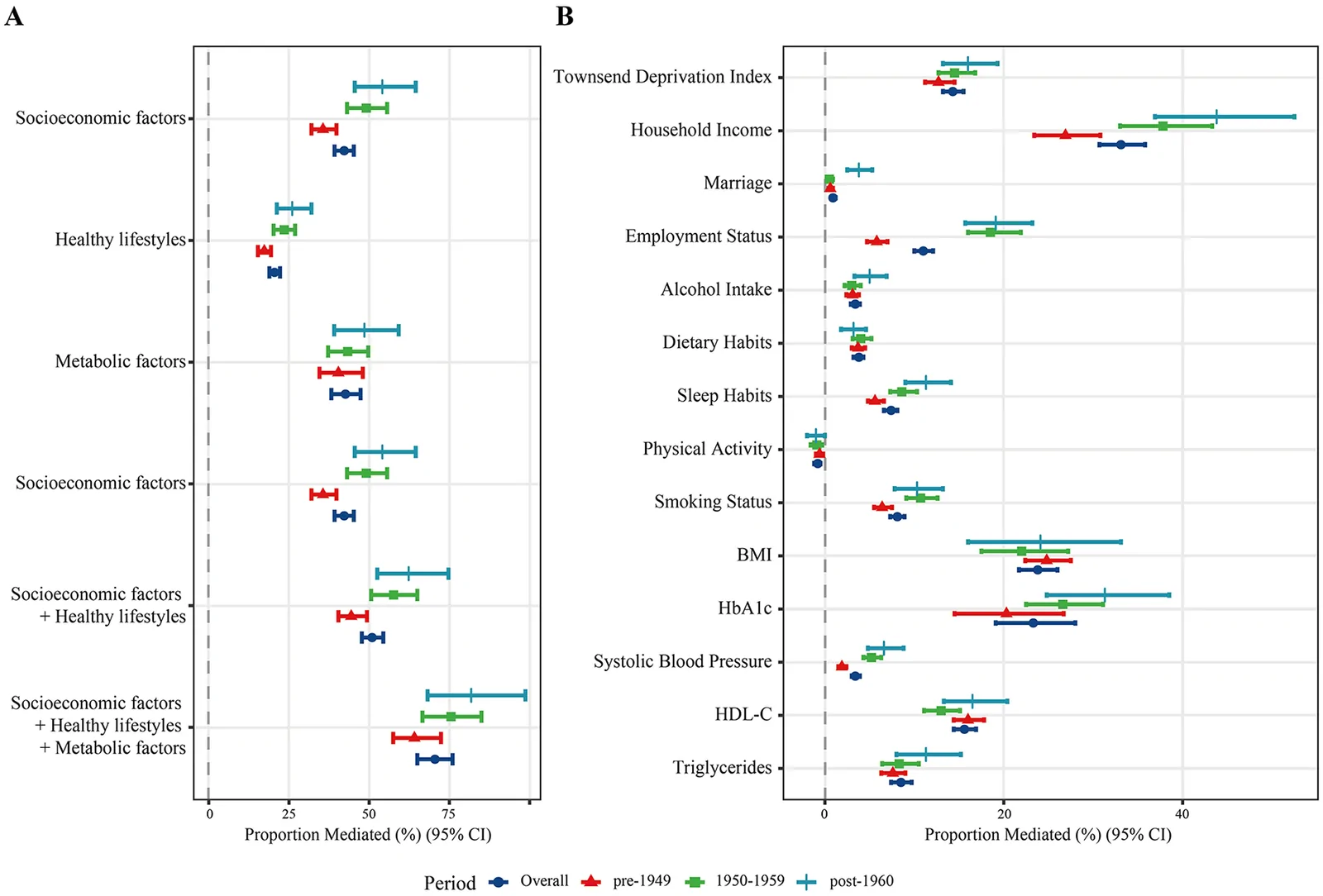
Mediation analysis of the association between educational attainment and incident type 2 diabetes. **(A)** The proportion of the effect mediated by broad categories of risk factors: socioeconomic, behavioral, and metabolic. **(B)** The proportion mediated by specific individual factors.

Further analysis of socioeconomic factors revealed that household income mediated the largest proportion [33.1% (30.7%−35.8%)], with a progressive increase from 26.9% (23.4%−30.8%) in the pre-1949 cohort to 37.8% (33.0%−43.3%) in the 1950–1959 cohort and 43.8% (36.9%−52.5%) in the post-1960 cohort. Regarding modifiable behavioral and metabolic risk factors, BMI [23.8% (21.7%−26.0%)] and HbA1c [23.3% (19.1%−28.0%)] contributed the most substantial mediation proportions (Figure 6B and Supplementary Table 24).

## 4 Discussion

### 4.1 Principal findings

This prospective cohort study leveraged data from the UK Biobank to investigate Education-related inequality in the risk of T2DM. Education-related inequalities in the risk of T2DM persist across all birth cohorts. Strikingly, despite policy initiatives driving the expansion of mass education (9), educational health inequities have paradoxically intensified. The rising prevalence of T2DM exacerbates the adverse impact associated with educational health inequities (1). Notably, among individuals with similarly low educational attainment, the absolute risk of incident T2DM is significantly higher in younger generations. Additionally, Education-related inequality is also observed in the subsequent risk of progression to CVD or mortality. Socioeconomic, behavioral, and metabolic factors collectively accounted for approximately 70% of the impact of education disparity on T2DM risk. Among these, socioeconomic factors were the most prominent mediator, and the proportion of risk mediated by these factors increased over time.

### 4.2 Widening education-related inequalities in the risk of T2DM

Educational attainment in early life profoundly influences health outcomes by shaping cognitive ability and health literacy, as well as by determining future socioeconomic status (11, 23, 24). Substantial evidence supports the association between higher educational attainment and reduced risks of mortality and a wide range of diseases (25–27). Consistent with existing evidence, we observed distinct Education-related inequality in T2DM risk in the overall population. However, it is particularly noteworthy that this inequality has widened across successive birth cohorts.

We postulate several explanations for this observed trend. First, following the widespread increase in education spurred by historic policies like the 1944 Education Reform (9), retaining a low education level in later cohorts may represent a marker of more profound early-life socioeconomic deprivation, a known critical determinant of long-term metabolic health (28, 29). Furthermore, in the post-industrial context, elevated requirements for skills and training translate into compounded disadvantages for the less-educated, leading to a deepening structural disparity in socioeconomic outcomes (30). Indeed, our data corroborated this by showing a concurrent widening of socioeconomic disparities across education levels over time.

Educational attainment is known to be partially heritable, a finding well-supported by prior UK Biobank studies (31). Furthermore, genetic variants associated with education demonstrate pleiotropic effects on health–related behaviors and metabolic traits, including smoking, BMI, and blood pressure (32). Consistent with this shared genetic architecture, our study indicates that individuals with lower educational attainment carry a significantly higher polygenic risk for T2DM. Notably, this genetic gradient has intensified in later birth cohorts. We postulate that following widespread educational expansion, the remaining population in lower educational strata represents a genetically stratified group, characterized by a higher concentration of susceptibility alleles and compounding disadvantages.

In our sensitivity analyses, we observed a significantly lower risk of T2DM in earlier birth cohorts compared to later ones, a trend that parallels previously reported data on disease burden (1). While this disparity may be partially attributable to temporal improvements in disease detection and selection bias related to intergenerational differences in health status within the UK Biobank population, it also implies that earlier cohorts were exposed to a risk factor profile characterized by a narrower range and lower prevalence. Specifically, over the past half-century, there has been a dramatic surge in the consumption of ultra-processed foods and high-sugar, high-fat diets, concomitant with a decline in physical activity and a rise in sedentary behaviors. The relatively lower burden of these risk factors in earlier birth cohorts may have attenuated the association between educational attainment and T2DM risk (33, 34).

This study investigated the impact of educational attainment on the entire disease trajectory of T2DM. We identified significant Education-related inequalities in the progression from diagnosis to CVDs and mortality, and observed that these disparities have widened across successive birth cohorts. Educational attainment serves as a pivotal determinant of the effectiveness of secondary prevention strategies. Diabetes self-management necessitates a multifaceted regimen that includes rigorous blood glucose monitoring, medication compliance, dietary compliance, and regular physical activity. Consequently, achieving optimal glycemic control imposes a high demand on health literacy, an attribute where individuals with higher educational backgrounds typically demonstrate superior performance (35–37).

### 4.3 Mediators linking educational attainment to T2DM risk

Previous studies in the UK have revealed that income inequality has intensified, concomitant with the widespread expansion of education (38). Educational attainment shapes socioeconomic status, especially household income, thereby determining access to healthcare, nutritious diets, and physical activity facilities (39–41). Our analysis identifies this socioeconomic pathway as the predominant mediator linking education to T2DM risk, with its relative contribution progressively increasing across successive birth cohorts. Education-related disparities in T2DM risk are significantly mediated by behavioral factors like smoking and sleep hygiene. Beyond the impact of limited health literacy on lifestyle adherence, it is crucial to recognize the occupational hazards associated with lower education. These individuals are more frequently compelled by economic necessity to accept roles involving shift work, leading to involuntary circadian disruption and adverse health behaviors (42). Intriguingly, we found that individuals with lower educational attainment exhibited higher levels of total physical activity, yielding a negative mediation effect. However, this elevation was likely driven by high levels of occupational physical activity (OPA) (43). Although existing evidence suggests that OPA may be detrimental to certain disease risks (44, 45), our findings indicate that the overall high physical load associated with OPA exerted an ameliorative effect on T2DM risk. In terms of metabolic pathway factors, BMI and HbA1c accounted for the largest explanatory proportions. However, HbA1c should be interpreted cautiously because it may partly reflect early glycaemic deterioration before clinical diagnosis rather than acting solely as an intermediate factor.

### 4.4 Implications for public health policy and clinical practice

In terms of public health policy, our findings advocate for targeted metabolic screening among individuals with lower educational attainment. Furthermore, interventions to bolster health literacy are warranted to offset the deficits resulting from early-life educational deprivation. Such strategies aim to optimize modifiable behavioral factors, thereby shifting the window for correcting metabolic abnormalities upstream. In clinical practice, following a diagnosis of T2DM, clinicians should exercise heightened vigilance when managing individuals with lower educational attainment, particularly those from younger birth cohorts. There is a critical need for more aggressive diabetes health education targeted at these vulnerable groups. Furthermore, it is necessary to integrate educational attainment into T2DM prognostic risk assessment frameworks to improve risk stratification.

### 4.5 Strengths and limitations

Our study has several distinctive strengths. First, we leveraged the UK Biobank, a large-scale, prospective cohort with high-quality data and long-term follow-up, which provided the statistical power necessary to robustly examine the association between education and T2DM risk. Second, to address potential biases arising from differences in baseline age across birth cohorts, we performed a rigorous sensitivity analysis by standardizing the start of follow-up to age 30. In addition, we performed an attained-age time-scale analysis with delayed entry at the age of recruitment, which directly accounted for differences in baseline age and left truncation across birth cohorts. This approach ensured a more valid intergenerational comparison. Third, capitalization on the fact that educational attainment is a fixed characteristic determined in early life, we employed a multi-state competing risk model. This allowed us to quantify the impact of education not just on disease incidence, but also on the subsequent trajectory of post-diagnostic progression.

Several limitations of this study merit consideration. First, data on educational attainment and lifestyle factors were derived from self-reported questionnaires, which may have introduced recall bias and measurement error. Second, the UK Biobank is subject to a well-documented healthy volunteer bias, as participants are generally healthier and wealthier than the general UK population; this may have led to an underestimation of the true magnitude of education-related inequalities. Third, selection bias may have arisen from the recruitment strategy and exclusion criteria. Because participants were recruited during the same calendar period, those from earlier birth cohorts may represent a survivor population with relatively better health. In addition, excluding participants with prevalent diabetes at baseline may have disproportionately removed individuals with early-onset disease from earlier cohorts, further selecting for a healthier subset. Fourth, as is common in UK Biobank studies, incident T2DM was identified using ICD-coded hospital inpatient records. Primary care and medication records were not available under our current UK Biobank data access; therefore, some outpatient-managed T2DM cases may have been missed, leading to potential underestimation of incidence. However, ICD-coded linked records provide prospectively collected, date-stamped diagnoses for time-to-event analyses and have been widely used in UK Biobank studies. Fifth, although we used the RII to depict trends in relative inequality, formal statistical comparisons of RII estimates across birth cohorts were not performed; therefore, specific inter-cohort differences in RII should be interpreted cautiously. Finally, the pathway analysis should be interpreted as explanatory rather than definitive causal mediation evidence. Although educational attainment was determined early in life and is unlikely to be affected by subsequent T2DM, some pathway factors may not represent definitive causal mediators. HbA1c may partly reflect preclinical glycaemic deterioration, and employment or retirement status is closely related to age. Moreover, although random forest-based imputation with predictive mean matching preserved the observed covariate distributions well, this approach does not formally propagate imputation uncertainty as multiple imputation would; therefore, residual uncertainty related to missing covariate data cannot be excluded.

## 5 Conclusion

As Graphical abstract shown In conclusion, this study demonstrates that educational inequalities extend beyond the onset of T2DM to significantly influence the risk of progression to CVD and mortality. Crucially, these disparities have not only persisted across successive birth cohorts but show a concerning widening trend. Given that these effects are largely mediated by modifiable socioeconomic, behavioral, and metabolic factors, individuals with lower educational attainment should be prioritized in both primary prevention strategies and clinical management protocols to mitigate the growing burden of T2DM.

## Data Availability

Publicly available datasets were analyzed in this study. This data can be found here: https://www.ukbiobank.ac.uk/.

## Author's note

This specific research was conducted using the UK Biobank Resource under Application Number 103736 UK Biobank. This project was duly registered and approved via the UK Biobank Access Management System (AMS), with authorised access granted for Tier 3 data. The principal applicant for this UK Biobank project is Professor Changsheng Ma, who is also a co-author of the present manuscript. All researchers involved in data analysis for this study are registered collaborators within this AMS project.
