# Supplementary material for: Widening educational inequalities in type 2 diabetes risk and mediators across successive generations: a prospective cohort study
Source: Front Public Health. 2026 Oct 6;14:1893269. doi: 10.3389/fpubh.2026.1893269 (PMC13680178; doi:10.3389/fpubh.2026.1893269)
Supplement: Supplementary file 1 [file Data_Sheet_1.docx]

Supplementary Methods

1. Definitions of Participant Characteristics
   (1) Diabetes status

| Category | Definition Criteria | Data Fields |
| --- | --- | --- |
| Hospital Inpatient Records | Diagnosis of diabetes based on ICD-10 codes (E10–E14) or ICD-9 codes (250.0–250.9) in hospital inpatient records, with a diagnosis date prior to the date of attending the assessment centre. | p53, p130706, p130708, p130710, p130712, p130714, p[41271](https://biobank.ndph.ox.ac.uk/showcase/field.cgi?id=41271), p[41281](https://biobank.ndph.ox.ac.uk/showcase/field.cgi?id=41281) |
| Self-reported History | Self-reported diagnosis of diabetes by a doctor during the touchscreen questionnaire or verbal interview at baseline. | p2976, p20002, p2443 |
| Medication Use | Self-reported use of glucose-lowering medications (e.g., insulin, metformin) at baseline. | p20003, p6177, p6153 |
| Fasting Plasma Glucose | Fasting plasma glucose level ≥ 7.0 mmol/L. | p74, p30740 |
| Random Plasma Glucose | Random plasma glucose level ≥ 11.1 mmol/L. | p74, p30740 |
| HbA1c | Glycated hemoglobin (HbA1c) level ≥ 6.5% (48 mmol/mol). | p30750 |

(2) Cardiovascular Disease

| Disease | Category | Definition Criteria | Data Fields |
| --- | --- | --- | --- |
| IHD | Hospital Records & Algorithmic Definitions & self-reported medical history | CAD was ascertained through hospital admission records using ICD-9 codes 410–414 and ICD-10 codes I20–I25, combined with algorithmically defined myocardial infarction events provided by UK Biobank. | p53, p[41271](https://biobank.ndph.ox.ac.uk/showcase/field.cgi?id=41271), p[41281](https://biobank.ndph.ox.ac.uk/showcase/field.cgi?id=41281), p42000, p42002, p42004, p131296, p131298, p131300, p131302, p131304, p131306 |
| Stroke | Hospital Records & Algorithmic Definitions & self-reported medical history | Stroke was ascertained through hospital admission records using ICD-9 codes 430–435 and ICD-10 codes I60–I64, combined with algorithmically defined stroke events provided by UK Biobank. | p53, p[41271](https://biobank.ndph.ox.ac.uk/showcase/field.cgi?id=41271), p[41281](https://biobank.ndph.ox.ac.uk/showcase/field.cgi?id=41281), p42006, p42008, p42010, p42012, p131360, p131362, p131364, p131366, p131368 |
| Heart failure | Hospital Records & self-reported medical history | Stroke was ascertained through hospital admission records using ICD-9 codes 428 and ICD-10 codes I50 | p53, p[41271](https://biobank.ndph.ox.ac.uk/showcase/field.cgi?id=41271), p[41281](https://biobank.ndph.ox.ac.uk/showcase/field.cgi?id=41281), p131354 |

(3) Socioeconomic factors
    1. **Townsend Deprivation Index (Field 22189):** Socioeconomic deprivation was assessed using the Townsend Deprivation Index (TDI). This index was calculated based on the preceding national census output areas, incorporating indicators of unemployment, non-car ownership, non-home ownership, and household overcrowding. Each participant was assigned a continuous score corresponding to the output area in which their postcode was located at the time of recruitment into the UK Biobank.
    2. **Employment status (Field 6142):** Employment status was reclassified into three categories: employed (including those in paid employment, self-employed, or students), retired, and unemployed (including unemployed, looking after home/family, or unable to work). Participants who responded "prefer not to answer," "none of the above," or had missing values were treated as missing data (NA).
    3. **Marital status:** Marital status was determined based on the relationship between the participant and their household members (Field 6141). Participants were defined as "married/co-habiting" if they reported a "husband, wife, or partner" living in the same household. Otherwise, they were classified as "not married/co-habiting".
    4. **Annual household income:** Average total household income before tax was self-reported by participants (Field 738) and categorized into five levels: less than £18,000, £18,000 to £30,999, £31,000 to £51,999, £52,000 to £100,000, and greater than £100,000. Participants who responded with "do not know" or "prefer not to answer" were treated as missing data.
(4) behavioral factors
    1. **Smoking status:** Smoking status was self-reported by participants (Field 20116) and categorized as never, previous, or current smokers. Participants who responded "prefer not to answer" or had missing data were treated as missing values.
    2. **Drinking Status and Alcohol Intake:** Alcohol consumption was assessed by integrating self-reported drinking history and a detailed quantification of weekly intake. For current drinkers, total weekly alcohol units were calculated by summing the consumption across various beverage types (Field 1568–5364 for weekly and 4407–4462 for monthly intake). Monthly frequencies were converted to weekly intake by dividing by 4.3.

To ensure standardized quantification, one alcohol unit was defined as 10 mL (8 g) of pure ethanol. Based on UK Biobank’s standard conversion factors, specific unit values were assigned to standard measures of different beverages:

- 1.5 units per glass of red wine, white wine, or champagne;
- 2.8 units per pint of beer or cider;
- 1.0 unit per standard measure of spirits or fortified wine (e.g., sherry or port);
- 1.5 units per measure of other alcoholic beverages.

Participants were subsequently categorized into four distinct groups according to their drinking history and the UK Chief Medical Officers' low-risk drinking guidelines:

- Never drinkers: Participants with no history of alcohol consumption;
- Previous drinkers: Those who had consumed alcohol in the past but not currently;
- Safe drinkers: Current drinkers consuming 0–14 units per week;
- Dangerous drinkers: Current drinkers consuming more than 14 units per week.

Participants who responded "Do not know," "Prefer not to answer," or had missing data for these fields were treated as missing values (NA).

1. **Dietary Habits:** Dietary quality was evaluated using a predefined healthy diet score based on ten food groups. Participants were assigned 1 point for each component if their reported intake met the specific health-oriented goal, and 0 points otherwise, utilizing data from the UK Biobank dietary questionnaires (Field IDs 1289–6144). A cumulative dietary score ranging from 0 to 10 was calculated by summing the points from all ten categories, and for subsequent analysis, diet quality was categorized as a binary variable where a total score of 4 or higher was defined as a Healthy Diet.

| Diet component | Intake goal | Field IDs | Amount per serving |
| --- | --- | --- | --- |
| Fruit | ≥3 servings/day | 1309 (pieces fresh fruit/day)  1319 (pieces dried fruit/day) | 1309 – 1 piece  1319 – 5 pieces |
| Vegetable | ≥3 servings/day | 1289 (tablespoons cooked vegetables/day)  1299 (salad/raw vegetables/day) | 3 heaped tablespoons |
| Whole grains | ≥3 servings/day | 1438, 1448 (whole meal/wholegrain bread slices/week)  1458, 1468 (bran/oat/muesli cereal bowls/week) | 1438/1448 – 1 slice/day  1458/1468 – 1 bowl/day |
| (Shell)fish | ≥2 servings/week | 1329 (oily fish/week)  1339 (non-oily fish/week) | Once/week |
| Dairy | ≥2 servings/day | 1408 (cheese/week)  1418 (milk type) | 1408 – 1 piece/day  1418 – 1 glass/day if consumption of any type of milk |
| Vegetable oils | ≥2 servings/day | 1428 (Flora Pro-Active/Benecol spread)  2654 (Flora Pro-Active/Benecol, soft margarine -, olive oil based -, polyunsaturated/sunflower oil based -, other low/reduced fat spread)  1438 (bread slices/week) | 1 serving/day if in combination with eating at least 2 slices of bread (ID 1438) |
| Refined grains | ≤2 servings/day | 1438, 1448 (white, brown, other bread slices/week)  1458, 1468 (biscuit, other cereals/week) | 1438/1448 – 1 slice/day  1458/1468 – 1 bowl/day |
| Processed meats | ≤1 serving/week | 1349 (processed meat/week or daily)  3680 (age when last ate meat) | 1349 – 1 piece/day  3680 – 0 pieces/day if indicated having never eaten meat |
| Unprocessed meats | ≤2 servings/week | 1359 (poultry/week or day)  1369 (beef/week or day)  1379 (lamb or mutton/week or day)  1389 (pork/week or day)  3680 (age when last ate meat) | 1359-1389 – once/week  3680 – 0 pieces/day if indicated having never eaten meat |
| Sugar-sweetened beverages | Don’t drink | 6144 (never consumes drinks containing sugar) | Only 0 servings were possible here. |

1. **Physical Activity Level:** Physical Activity Level: Physical activity was classified according to the UK Biobank-derived MET-based physical activity category (Field ID 22032). Participants were categorized as having low, moderate, or high physical activity levels. This three-level classification was used in the main analyses.
2. **Sleep Status:** Sleep duration was derived from the self-reported average number of hours spent sleeping in a 24-hour period (Field ID 1160). A sleep score was assigned according to sleep duration: 100 points for 7 to <9 hours, 90 points for 9 to <10 hours, 70 points for 6 to <7 hours, 40 points for 5 to <6 hours or >=10 hours, 20 points for 4 to <5 hours, and 0 points for <4 hours. Participants were then categorized as having healthy sleep (score = 100), medium sleep (score = 70 or 90), or unhealthy sleep (score <=40).
(5) Metabolic factors

| Metabolic factors | Field |
| --- | --- |
| Body Mass Index | p21001 |
| HDL-C | p30760 |
| Triglycerides | p30870 |
| Glycated haemoglobin | p30750 |
| Systolic Blood Pressure | p4080 |
