# Supplementary material for: Widening educational inequalities in type 2 diabetes risk and mediators across successive generations: a prospective cohort study
Source: Front Public Health. 2026 Oct 6;14:1893269. doi: 10.3389/fpubh.2026.1893269 (PMC13680178; doi:10.3389/fpubh.2026.1893269)
Supplement: Supplementary file 2 [file Data_Sheet_2.docx]

**Electronic Supplementary Material Results**

[Supplementary Figure 1. Flow diagram illustrating the inclusion and exclusion process.	3](#_Toc9321)

[Supplementary Figure 2. Distribution of Categorical Variables: Before vs After Imputation.	4](#_Toc15347)

[Supplementary Figure 3. Distribution of Continuous Variables: Before vs After Imputation.	5](#_Toc20741)

[Supplementary Figure 4. Distribution of cognitive test performance levels stratified by educational attainment.	6](#_Toc25451)

[Supplementary Table 1. Baseline characteristics of the final analytical cohort according to educational attainment (Participants born before 1949).	7](#_Toc18774)

[Supplementary Table 2. Baseline characteristics of the final analytical cohort according to educational attainment (Participants born in 1950 - 1959).	10](#_Toc19056)

[Supplementary Table 3. Baseline characteristics of the final analytical cohort according to educational attainment (Participants born after 1960).	13](#_Toc20483)

[Supplementary Table 4. Distribution of Education Level by Birth Year and Sex.	16](#_Toc80)

[Supplementary Table 5. 5-Year and 10-Year Risk of T2DM Stratified by Birth Year.	19](#_Toc22854)

[Supplementary Table 6. Numbers at risk for incident T2DM by educational attainment and birth cohort.	21](#_Toc18943)

[Supplementary Table 7. Association between education level and risk of T2DM stratified by birth year and sex across progressively adjusted models.	23](#_Toc6519)

[Supplementary Table 8. Association between education level and T2DM risk stratified by birth year and sex, adjusted for distinct categories of covariates.	26](#_Toc18651)

[Supplementary Table 9. P values for education-by-birth-cohort interaction corresponding to Supplementary Table 6.	29](#_Toc6454)

[Supplementary Table 10. P values for education-by-birth-cohort interaction corresponding to Supplementary Table 7.	30](#_Toc9405)

[Supplementary Table 11. Cause-specific Cox analysis of the association between educational attainment and incident T2DM.	31](#_Toc978)

[Supplementary Table 12. Proportional hazards assumption tests for the cause-specific Cox models.	36](#_Toc15171)

[Supplementary Table 13. 10-year, 20-year, and 30-year risk of T2DM starting at age 30.	49](#_Toc18897)

[Supplementary Table 14. Association between education level and risk of cardiovascular disease starting at age 30.	51](#_Toc9911)

[Supplementary Table 15. Association between educational attainment and incident T2DM using attained age as the time scale with delayed entry	53](#_Toc16580)

[Supplementary Table 16. Education-related Relative Index of Inequality (RII) in risk of T2DM stratified by birth year and sex across progressively adjusted models.	57](#_Toc16652)

[Supplementary Table 17. Education-related Relative Index of Inequality (RII) in risk of T2DM stratified by birth year and sex, adjusting separately for distinct categories of covariates.	58](#_Toc29400)

[Supplementary Table 18. Absolute educational inequalities in 10-year risk of incident T2DM.	59](#_Toc11192)

[Supplementary Table 19. Education-related Relative Index of Inequality (RII) in risk of T2DM starting at age 30.	60](#_Toc9146)

[Supplementary Table 20. Hazard ratios for transitions between health, T2DM, and death according to education level in a multi-state model.	61](#_Toc27518)

[Supplementary Table 21. Hazard ratios for transitions between health, T2DM, and CVD according to education level in a multi-state model starting at age 30.	64](#_Toc32199)

[Supplementary Table 22. Transition-specific risk-set sizes and event counts in the multi-state models.	67](#_Toc24416)

[Supplementary Table 23. Proportion of the association between education level and T2DM risk mediated by socioeconomic, behavioral, and metabolic factors, individually and jointly, stratified by birth year and sex.	68](#_Toc12710)

[Supplementary Table 24. Proportion of the association between education level and T2DM risk mediated by specific socioeconomic, behavioral, and metabolic risk factors, stratified by birth year and sex.	72](#_Toc21589)

Supplementary Figure 1. Flow diagram illustrating the inclusion and exclusion process.

Supplementary Figure 2. Distribution of Categorical Variables: Before vs After Imputation.

Supplementary Figure 3. Distribution of Continuous Variables: Before vs After Imputation.

Supplementary Figure 4. Distribution of cognitive test performance levels stratified by educational attainment.

The stacked bar charts illustrate the proportion of participants categorized into Low, Medium, and High cognitive performance groups across four educational levels (College Education, High School, Middle School, and Primary School or Less). The panels display results for four distinct cognitive domains: (A) Pairs Matching; (B) Fluid Intelligence; (C) Reaction Time; and (D) Numeric Memory. Cognitive performance levels were defined based on the tertiles of the respective test scores. The colors represent the performance categories: Low (blue), Medium (yellow), and High (grey).

Supplementary Table 1. Baseline characteristics of the final analytical cohort according to educational attainment (Participants born before 1949).

| Variables | level | Overall | College Education | High School | Middle School | Primary School or Less | p | SMD |
| --- | --- | --- | --- | --- | --- | --- | --- | --- |
|  |  | 211734 | 83742 | 38958 | 33990 | 55044 |  |  |
| age, years, mean (SD) |  | 63.65 (3.12) | 63.27 (3.08) | 63.67 (3.09) | 63.58 (3.08) | 64.26 (3.11) | <0.001 | 0.166 |
| sex n (%) | Female | 115023 ( 54.3) | 37960 ( 45.3) | 23322 ( 59.9) | 22708 ( 66.8) | 31033 ( 56.4) | <0.001 | 0.232 |
|  | Male | 96711 ( 45.7) | 45782 ( 54.7) | 15636 ( 40.1) | 11282 ( 33.2) | 24011 ( 43.6) |  |  |
| ethnicity n (%) | non-White | 3984 ( 1.9) | 1759 ( 2.1) | 791 ( 2.0) | 552 ( 1.6) | 882 ( 1.6) | <0.001 | 0.024 |
|  | White | 206193 ( 98.1) | 81147 ( 97.9) | 37863 ( 98.0) | 33264 ( 98.4) | 53919 ( 98.4) |  |  |
| PRS of T2DM, mean (SD) |  | -0.07 (0.98) | -0.11 (0.98) | -0.09 (0.98) | -0.06 (0.97) | -0.01 (0.97) | <0.001 | 0.054 |
| **Socioeconomic factors** |  |  |  |  |  |  |  |  |
| Townsend Deprivation Index, median [IQR] |  | -2.42 [-3.78, -0.08] | -2.60 [-3.90, -0.43] | -2.73 [-3.96, -0.81] | -2.63 [-3.86, -0.67] | -1.66 [-3.29, 1.34] | <0.001 | 0.218 |
| Household income, n (%) | Less than 18,000 | 55718 ( 32.0) | 14001 ( 18.9) | 8181 ( 25.3) | 9784 ( 35.6) | 23752 ( 59.0) | <0.001 | 0.573 |
|  | 18,000 to 30,999 | 56359 ( 32.4) | 23253 ( 31.4) | 11790 ( 36.4) | 10108 ( 36.8) | 11208 ( 27.8) |  |  |
|  | 31,000 to 51,999 | 38078 ( 21.9) | 20771 ( 28.0) | 7964 ( 24.6) | 5327 ( 19.4) | 4016 ( 10.0) |  |  |
|  | 52,000 to 100,000 | 19374 ( 11.1) | 12724 ( 17.2) | 3669 ( 11.3) | 1915 ( 7.0) | 1066 ( 2.6) |  |  |
|  | Greater than 100,000 | 4652 ( 2.7) | 3369 ( 4.5) | 744 ( 2.3) | 321 ( 1.2) | 218 ( 0.5) |  |  |
| marriage, n (%) | married/co-habiting | 156537 ( 93.9) | 63335 ( 94.4) | 28880 ( 94.0) | 25365 ( 94.1) | 38957 ( 92.9) | <0.001 | 0.03 |
|  | not married/co-habiting | 10180 ( 6.1) | 3789 ( 5.6) | 1847 ( 6.0) | 1585 ( 5.9) | 2959 ( 7.1) |  |  |
| employment status, n (%) | Employed | 64346 ( 30.6) | 31150 ( 37.4) | 11247 ( 29.1) | 9542 ( 28.2) | 12407 ( 22.7) | <0.001 | 0.187 |
|  | Unemployed | 8658 ( 4.1) | 2535 ( 3.0) | 1243 ( 3.2) | 1430 ( 4.2) | 3450 ( 6.3) |  |  |
|  | Retired | 137259 ( 65.3) | 49515 ( 59.5) | 26186 ( 67.7) | 22828 ( 67.5) | 38730 ( 71.0) |  |  |
| **Behavioral factors** |  |  |  |  |  |  |  |  |
| alcohol intake, n (%) | Never | 9273 ( 5.2) | 2596 ( 3.5) | 1772 ( 5.4) | 1505 ( 5.4) | 3400 ( 7.8) | <0.001 | 0.159 |
|  | Previous | 7455 ( 4.2) | 2404 ( 3.3) | 1185 ( 3.6) | 1064 ( 3.8) | 2802 ( 6.5) |  |  |
|  | Safe | 86040 ( 48.5) | 34610 ( 47.3) | 16578 ( 50.7) | 14820 ( 52.7) | 20032 ( 46.2) |  |  |
|  | Dangerous | 74577 ( 42.1) | 33561 ( 45.9) | 13143 ( 40.2) | 10716 ( 38.1) | 17157 ( 39.5) |  |  |
| dietary habits, n (%) | Healthy | 108285 ( 64.6) | 44958 ( 66.9) | 20839 ( 66.5) | 17570 ( 64.1) | 24918 ( 59.7) | <0.001 | 0.083 |
|  | Unhealthy | 59369 ( 35.4) | 22241 ( 33.1) | 10483 ( 33.5) | 9830 ( 35.9) | 16815 ( 40.3) |  |  |
| sleep habits, n (%) | Unhealthy | 15328 ( 7.3) | 4470 ( 5.4) | 2513 ( 6.5) | 2403 ( 7.1) | 5942 ( 10.9) | <0.001 | 0.133 |
|  | Medium | 53398 ( 25.4) | 19803 ( 23.7) | 9482 ( 24.5) | 8810 ( 26.0) | 15303 ( 28.2) |  |  |
|  | Healthy | 141724 ( 67.3) | 59224 ( 70.9) | 26784 ( 69.1) | 22611 ( 66.8) | 33105 ( 60.9) |  |  |
| physical activity, n (%) | low | 25979 ( 16.3) | 11098 ( 16.1) | 4850 ( 16.1) | 4327 ( 17.1) | 5704 ( 16.2) | <0.001 | 0.063 |
|  | moderate | 66405 ( 41.6) | 29621 ( 43.1) | 12789 ( 42.4) | 10672 ( 42.1) | 13323 ( 37.9) |  |  |
|  | high | 67061 ( 42.1) | 28066 ( 40.8) | 12551 ( 41.6) | 10361 ( 40.9) | 16083 ( 45.8) |  |  |
| smoking status, n (%) | Never | 107418 ( 51.0) | 44460 ( 53.2) | 20582 ( 53.0) | 17493 ( 51.6) | 24883 ( 45.6) | <0.001 | 0.105 |
|  | Previous | 85856 ( 40.7) | 33341 ( 39.9) | 15489 ( 39.9) | 13709 ( 40.5) | 23317 ( 42.7) |  |  |
|  | Current | 17524 ( 8.3) | 5697 ( 6.8) | 2782 ( 7.2) | 2686 ( 7.9) | 6359 ( 11.7) |  |  |
| **Metabolic factors** |  |  |  |  |  |  |  |  |
| SBP, mmHg, (mean (SD)) |  | 143.27 (18.81) | 142.25 (18.65) | 142.91 (18.72) | 143.26 (18.74) | 145.11 (19.03) | <0.001 | 0.079 |
| BMI, kg/m2, (mean (SD)) |  | 27.28 (4.36) | 26.92 (4.20) | 27.13 (4.35) | 27.21 (4.36) | 28.00 (4.50) | <0.001 | 0.127 |
| Waist, cm, mean (SD) |  | 90.61 (12.63) | 90.67 (12.39) | 89.70 (12.73) | 89.14 (12.57) | 92.05 (12.80) | <0.001 | 0.128 |
| HbA1c, %, (mean (SD)) |  | 5.44 (0.33) | 5.41 (0.33) | 5.44 (0.33) | 5.44 (0.32) | 5.47 (0.34) | <0.001 | 0.083 |
| FPG, mmol/L, (mean (SD)) |  | 5.08 (0.53) | 5.11 (0.53) | 5.09 (0.53) | 5.07 (0.51) | 5.07 (0.54) | 0.1 | 0.045 |
| TC, mmol/L, (mean (SD)) |  | 5.82 (1.17) | 5.80 (1.15) | 5.88 (1.16) | 5.92 (1.16) | 5.75 (1.21) | <0.001 | 0.084 |
| HDL-C, mmol/L, (mean (SD)) |  | 1.48 (0.39) | 1.48 (0.39) | 1.51 (0.39) | 1.52 (0.39) | 1.44 (0.37) | <0.001 | 0.121 |
| LDL, mmol/L, (mean (SD)) |  | 3.64 (0.88) | 3.63 (0.86) | 3.67 (0.88) | 3.69 (0.88) | 3.60 (0.91) | <0.001 | 0.062 |
| TG, mmol/L, (median [IQR]) |  | 1.54 [1.12, 2.16] | 1.51 [1.09, 2.13] | 1.52 [1.11, 2.14] | 1.53 [1.12, 2.13] | 1.61 [1.17, 2.25] | <0.001 | 0.053 |
| **Comorbidities** |  |  |  |  |  |  |  |  |
| CVD, n (%) |  | 21201 ( 10.0) | 7253 ( 8.7) | 3511 ( 9.0) | 2901 ( 8.5) | 7536 ( 13.7) | <0.001 | 0.084 |
| CAD, n (%) |  | 16408 ( 7.7) | 5519 ( 6.6) | 2717 ( 7.0) | 2198 ( 6.5) | 5974 ( 10.9) | <0.001 | 0.081 |
| Stroke, n (%) |  | 5532 ( 2.6) | 1888 ( 2.3) | 940 ( 2.4) | 801 ( 2.4) | 1903 ( 3.5) | <0.001 | 0.037 |
| HF n (%) |  | 1444 ( 0.7) | 480 ( 0.6) | 233 ( 0.6) | 165 ( 0.5) | 566 ( 1.0) | <0.001 | 0.032 |

*Supplementary Table 1 presents the data distribution of participants born before 1949. SD, Standard deviation; SMD, Standardized mean difference; PRS, Polygenic risk score; T2DM, Type 2 diabetes mellitus; IQR, Interquartile range; BMI, Body mass index; HbA1c, Hemoglobin A1c (Glycated hemoglobin); SBP, Systolic blood pressure; TC, Total cholesterol; HDL-C,* *High-density lipoprotein* *cholesterol; LDL-C, Low-density lipoprotein cholesterol; TG, Triglycerides; CVD, Cardiovascular disease; CAD, Coronary artery disease; HF, Heart failure.*

Supplementary Table 2. Baseline characteristics of the final analytical cohort according to educational attainment (Participants born in 1950 - 1959).

| Variables | level | Overall | College Education | High School | Middle School | Primary School or Less | p | SMD |
| --- | --- | --- | --- | --- | --- | --- | --- | --- |
|  |  | 149849 | 81146 | 26773 | 25068 | 16862 |  |  |
| age, years, mean (SD) |  | 53.83 (3.02) | 53.72 (3.02) | 53.95 (3.02) | 53.65 (3.08) | 54.47 (2.87) | <0.001 | 0.151 |
| sex n (%) | Female | 85641 ( 57.2) | 43538 ( 53.7) | 16577 ( 61.9) | 16250 ( 64.8) | 9276 ( 55.0) | <0.001 | 0.138 |
|  | Male | 64208 ( 42.8) | 37608 ( 46.3) | 10196 ( 38.1) | 8818 ( 35.2) | 7586 ( 45.0) |  |  |
| ethnicity n (%) | non-White | 6284 ( 4.2) | 3370 ( 4.2) | 1017 ( 3.8) | 1046 ( 4.2) | 851 ( 5.1) | <0.001 | 0.031 |
|  | White | 141652 ( 95.8) | 76605 ( 95.8) | 25460 ( 96.2) | 23815 ( 95.8) | 15772 ( 94.9) |  |  |
| PRS of T2DM, mean (SD) |  | -0.04 (0.99) | -0.06 (0.99) | -0.04 (0.98) | -0.01 (0.98) | 0.06 (0.98) | <0.001 | 0.066 |
| **Socioeconomic factors** |  |  |  |  |  |  |  |  |
| Townsend Deprivation Index, median [IQR] |  | -2.16 [-3.67, 0.48] | -2.30 [-3.76, 0.18] | -2.45 [-3.81, -0.15] | -2.16 [-3.59, 0.39] | -0.48 [-2.76, 2.80] | <0.001 | 0.294 |
| Household income, n (%) | Less than 18,000 | 19546 ( 14.7) | 7565 ( 10.1) | 2869 ( 12.0) | 4140 ( 19.2) | 4972 ( 38.0) | <0.001 | 0.593 |
|  | 18,000 to 30,999 | 27742 ( 20.8) | 12606 ( 16.9) | 5197 ( 21.8) | 5982 ( 27.8) | 3957 ( 30.3) |  |  |
|  | 31,000 to 51,999 | 39521 ( 29.7) | 21883 ( 29.3) | 7786 ( 32.6) | 6979 ( 32.4) | 2873 ( 22.0) |  |  |
|  | 52,000 to 100,000 | 36476 ( 27.4) | 24818 ( 33.2) | 6621 ( 27.7) | 3905 ( 18.1) | 1132 ( 8.7) |  |  |
|  | Greater than 100,000 | 9855 ( 7.4) | 7805 ( 10.5) | 1389 ( 5.8) | 523 ( 2.4) | 138 ( 1.1) |  |  |
| marriage, n (%) | married/co-habiting | 108637 ( 87.3) | 59563 ( 88.0) | 19749 ( 87.6) | 18098 ( 86.2) | 11227 ( 84.4) | <0.001 | 0.059 |
|  | not married/co-habiting | 15874 ( 12.7) | 8107 ( 12.0) | 2804 ( 12.4) | 2891 ( 13.8) | 2072 ( 15.6) |  |  |
| employment status, n (%) | Employed | 119897 ( 80.8) | 68552 ( 85.2) | 21113 ( 79.6) | 19328 ( 77.8) | 10904 ( 66.0) | <0.001 | 0.303 |
|  | Unemployed | 16899 ( 11.4) | 6140 ( 7.6) | 2739 ( 10.3) | 3481 ( 14.0) | 4539 ( 27.5) |  |  |
|  | Retired | 11601 ( 7.8) | 5813 ( 7.2) | 2677 ( 10.1) | 2023 ( 8.1) | 1088 ( 6.6) |  |  |
| **Behavioral factors** |  |  |  |  |  |  |  |  |
| alcohol intake, n (%) | Never | 5154 ( 4.1) | 2357 ( 3.4) | 903 ( 4.0) | 915 ( 4.5) | 979 ( 7.5) | <0.001 | 0.151 |
|  | Previous | 5023 ( 4.0) | 2365 ( 3.4) | 852 ( 3.8) | 872 ( 4.3) | 934 ( 7.1) |  |  |
|  | Safe | 59141 ( 47.0) | 33092 ( 47.4) | 11024 ( 49.1) | 9781 ( 47.7) | 5244 ( 40.1) |  |  |
|  | Dangerous | 56540 ( 44.9) | 32013 ( 45.8) | 9692 ( 43.1) | 8916 ( 43.5) | 5919 ( 45.3) |  |  |
| dietary habits, n (%) | Healthy | 68207 ( 58.7) | 38954 ( 61.1) | 12520 ( 59.8) | 10813 ( 55.3) | 5920 ( 49.9) | <0.001 | 0.128 |
|  | Unhealthy | 47946 ( 41.3) | 24845 ( 38.9) | 8429 ( 40.2) | 8727 ( 44.7) | 5945 ( 50.1) |  |  |
| sleep habits, n (%) | Unhealthy | 10513 ( 7.1) | 4595 ( 5.7) | 1689 ( 6.3) | 1942 ( 7.8) | 2287 ( 13.8) | <0.001 | 0.173 |
|  | Medium | 37627 ( 25.2) | 19581 ( 24.2) | 6691 ( 25.1) | 6584 ( 26.4) | 4771 ( 28.8) |  |  |
|  | Healthy | 100916 ( 67.7) | 56719 ( 70.1) | 18284 ( 68.6) | 16416 ( 65.8) | 9497 ( 57.4) |  |  |
| physical activity, n (%) | low | 23842 ( 20.1) | 13408 ( 19.8) | 4392 ( 20.6) | 3933 ( 20.5) | 2109 ( 20.3) | <0.001 | 0.107 |
|  | moderate | 48139 ( 40.6) | 28549 ( 42.2) | 8621 ( 40.3) | 7548 ( 39.4) | 3421 ( 32.9) |  |  |
|  | high | 46642 ( 39.3) | 25715 ( 38.0) | 8358 ( 39.1) | 7690 ( 40.1) | 4879 ( 46.9) |  |  |
| smoking status, n (%) | Never | 85946 ( 57.5) | 48651 ( 60.1) | 15688 ( 58.7) | 14161 ( 56.6) | 7446 ( 44.4) | <0.001 | 0.209 |
|  | Previous | 46745 ( 31.3) | 24906 ( 30.8) | 8352 ( 31.3) | 7767 ( 31.1) | 5720 ( 34.1) |  |  |
|  | Current | 16757 ( 11.2) | 7426 ( 9.2) | 2667 ( 10.0) | 3072 ( 12.3) | 3592 ( 21.4) |  |  |
| **Metabolic factors** |  |  |  |  |  |  |  |  |
| SBP, mmHg, (mean (SD)) |  | 135.39 (17.61) | 134.73 (17.37) | 135.26 (17.67) | 135.96 (17.77) | 137.98 (18.14) | <0.001 | 0.098 |
| BMI, kg/m2, (mean (SD)) |  | 27.21 (4.77) | 26.92 (4.63) | 27.18 (4.77) | 27.47 (4.85) | 28.27 (5.12) | <0.001 | 0.149 |
| Waist, cm, mean (SD) |  | 89.12 (13.25) | 88.80 (13.03) | 88.55 (13.28) | 88.83 (13.39) | 92.00 (13.69) | <0.001 | 0.128 |
| HbA1c, %, (mean (SD)) |  | 5.33 (0.33) | 5.32 (0.33) | 5.33 (0.33) | 5.34 (0.33) | 5.40 (0.34) | <0.001 | 0.125 |
| FPG, mmol/L, (mean (SD)) |  | 5.00 (0.52) | 4.99 (0.53) | 5.03 (0.51) | 5.00 (0.51) | 4.99 (0.53) | 0.156 | 0.045 |
| TC, mmol/L, (mean (SD)) |  | 5.86 (1.07) | 5.84 (1.06) | 5.88 (1.07) | 5.88 (1.06) | 5.85 (1.12) | <0.001 | 0.023 |
| HDL-C, mmol/L, (mean (SD)) |  | 1.48 (0.39) | 1.48 (0.39) | 1.50 (0.39) | 1.49 (0.38) | 1.42 (0.37) | <0.001 | 0.105 |
| LDL, mmol/L, (mean (SD)) |  | 3.67 (0.82) | 3.66 (0.82) | 3.68 (0.82) | 3.68 (0.81) | 3.69 (0.86) | <0.001 | 0.017 |
| TG, mmol/L, (median [IQR]) |  | 1.44 [1.02, 2.11] | 1.42 [1.00, 2.07] | 1.43 [1.01, 2.10] | 1.45 [1.03, 2.11] | 1.57 [1.11, 2.29] | <0.001 | 0.083 |
| **Comorbidities** |  |  |  |  |  |  |  |  |
| CVD, n (%) |  | 6048 ( 4.0) | 2740 ( 3.4) | 1003 ( 3.7) | 994 ( 4.0) | 1311 ( 7.8) | <0.001 | 0.099 |
| CAD, n (%) |  | 4243 ( 2.8) | 1894 ( 2.3) | 665 ( 2.5) | 683 ( 2.7) | 1001 ( 5.9) | <0.001 | 0.094 |
| Stroke, n (%) |  | 1922 ( 1.3) | 877 ( 1.1) | 333 ( 1.2) | 333 ( 1.3) | 379 ( 2.2) | <0.001 | 0.047 |
| HF n (%) |  | 387 ( 0.3) | 176 ( 0.2) | 68 ( 0.3) | 67 ( 0.3) | 76 ( 0.5) | <0.001 | 0.021 |

*Supplementary Table 1 presents the data distribution of participants born in 1950 - 1959. SD, Standard deviation; SMD, Standardized mean difference; PRS, Polygenic risk score; T2DM, Type 2 diabetes mellitus; IQR, Interquartile range; BMI, Body mass index; HbA1c, Hemoglobin A1c (Glycated hemoglobin); SBP, Systolic blood pressure; TC, Total cholesterol; HDL-C, High-density lipoprotein cholesterol; LDL-C, Low-density lipoprotein cholesterol;TG, Triglycerides; CVD, Cardiovascular disease; CAD, Coronary artery disease; HF, Heart failure.*

Supplementary Table 3. Baseline characteristics of the final analytical cohort according to educational attainment (Participants born after 1960).

| Variables | level | Overall | College Education | High School | Middle School | Primary School or Less | p | SMD |
| --- | --- | --- | --- | --- | --- | --- | --- | --- |
|  |  | 99214 | 59798 | 15439 | 19167 | 4810 |  |  |
| age, years, mean (SD) |  | 44.50 (2.57) | 44.43 (2.57) | 44.59 (2.55) | 44.58 (2.57) | 44.79 (2.52) | <0.001 | 0.072 |
| sex n (%) | Female | 54648 ( 55.1) | 31905 ( 53.4) | 9422 ( 61.0) | 11151 ( 58.2) | 2170 ( 45.1) | <0.001 | 0.177 |
|  | Male | 44566 ( 44.9) | 27893 ( 46.6) | 6017 ( 39.0) | 8016 ( 41.8) | 2640 ( 54.9) |  |  |
| ethnicity n (%) | non-White | 7787 ( 8.0) | 4702 ( 8.0) | 1049 ( 6.9) | 1272 ( 6.7) | 764 ( 16.7) | <0.001 | 0.165 |
|  | White | 89574 ( 92.0) | 53916 ( 92.0) | 14132 ( 93.1) | 17715 ( 93.3) | 3811 ( 83.3) |  |  |
| PRS of T2DM, mean (SD) |  | 0.00 (1.00) | -0.02 (1.00) | -0.01 (1.00) | 0.05 (0.99) | 0.13 (1.01) | <0.001 | 0.089 |
| **Socioeconomic factors** |  |  |  |  |  |  |  |  |
| Townsend Deprivation Index, median [IQR] |  | -1.64 [-3.41, 1.26] | -1.73 [-3.48, 1.04] | -2.10 [-3.60, 0.53] | -1.52 [-3.27, 1.42] | 1.74 [-1.32, 4.37] | <0.001 | 0.445 |
| Household income, n (%) | Less than 18,000 | 11451 ( 12.7) | 5184 ( 9.3) | 1456 ( 10.3) | 3178 ( 18.8) | 1633 ( 45.8) | <0.001 | 0.702 |
|  | 18,000 to 30,999 | 16306 ( 18.1) | 8365 ( 15.1) | 2644 ( 18.8) | 4353 ( 25.7) | 944 ( 26.5) |  |  |
|  | 31,000 to 51,999 | 27586 ( 30.6) | 16343 ( 29.5) | 4801 ( 34.1) | 5765 ( 34.0) | 677 ( 19.0) |  |  |
|  | 52,000 to 100,000 | 26994 ( 30.0) | 19319 ( 34.8) | 4251 ( 30.2) | 3150 ( 18.6) | 274 ( 7.7) |  |  |
|  | Greater than 100,000 | 7705 ( 8.6) | 6236 ( 11.2) | 936 ( 6.6) | 495 ( 2.9) | 38 ( 1.1) |  |  |
| marriage, n (%) | married/co-habiting | 70816 ( 83.7) | 43351 ( 85.5) | 11149 ( 83.7) | 13512 ( 81.2) | 2804 ( 71.7) | <0.001 | 0.181 |
|  | not married/co-habiting | 13785 ( 16.3) | 7374 ( 14.5) | 2175 ( 16.3) | 3127 ( 18.8) | 1109 ( 28.3) |  |  |
| employment status, n (%) | Employed | 87951 ( 89.4) | 54629 ( 92.0) | 13806 ( 90.0) | 16431 ( 86.4) | 3085 ( 66.6) | <0.001 | 0.35 |
|  | Unemployed | 10121 ( 10.3) | 4627 ( 7.8) | 1473 ( 9.6) | 2498 ( 13.1) | 1523 ( 32.9) |  |  |
|  | Retired | 308 ( 0.3) | 148 ( 0.2) | 55 ( 0.4) | 83 ( 0.4) | 22 ( 0.5) |  |  |
| **Behavioral factors** |  |  |  |  |  |  |  |  |
| alcohol intake, n (%) | Never | 4342 ( 5.3) | 2317 ( 4.6) | 620 ( 4.9) | 782 ( 5.1) | 623 ( 17.1) | <0.001 | 0.279 |
|  | Previous | 2959 ( 3.6) | 1596 ( 3.2) | 435 ( 3.4) | 638 ( 4.2) | 290 ( 8.0) |  |  |
|  | Safe | 39158 ( 47.6) | 24839 ( 49.1) | 6245 ( 49.1) | 6886 ( 45.0) | 1188 ( 32.6) |  |  |
|  | Dangerous | 35789 ( 43.5) | 21821 ( 43.1) | 5420 ( 42.6) | 7005 ( 45.8) | 1543 ( 42.3) |  |  |
| dietary habits, n (%) | Healthy | 38140 ( 50.2) | 24538 ( 52.6) | 5907 ( 49.2) | 6492 ( 45.0) | 1203 ( 41.1) | <0.001 | 0.13 |
|  | Unhealthy | 37826 ( 49.8) | 22071 ( 47.4) | 6095 ( 50.8) | 7938 ( 55.0) | 1722 ( 58.9) |  |  |
| sleep habits, n (%) | Unhealthy | 6159 ( 6.2) | 3021 ( 5.1) | 911 ( 5.9) | 1456 ( 7.6) | 771 ( 16.5) | <0.001 | 0.239 |
|  | Medium | 22565 ( 22.9) | 13116 ( 22.0) | 3448 ( 22.4) | 4664 ( 24.5) | 1337 ( 28.6) |  |  |
|  | Healthy | 69988 ( 70.9) | 43463 ( 72.9) | 11035 ( 71.7) | 12930 ( 67.9) | 2560 ( 54.8) |  |  |
| physical activity, n (%) | low | 14999 ( 18.4) | 9168 ( 17.9) | 2398 ( 18.6) | 2799 ( 18.9) | 634 ( 22.4) | <0.001 | 0.153 |
|  | moderate | 31990 ( 39.2) | 20708 ( 40.5) | 5157 ( 40.0) | 5344 ( 36.1) | 781 ( 27.6) |  |  |
|  | high | 34635 ( 42.4) | 21231 ( 41.5) | 5332 ( 41.4) | 6660 ( 45.0) | 1412 ( 49.9) |  |  |
| smoking status, n (%) | Never | 60837 ( 61.5) | 37965 ( 63.6) | 9793 ( 63.5) | 11017 ( 57.6) | 2062 ( 43.4) | <0.001 | 0.297 |
|  | Previous | 24281 ( 24.5) | 14727 ( 24.7) | 3664 ( 23.8) | 4748 ( 24.8) | 1142 ( 24.0) |  |  |
|  | Current | 13836 ( 14.0) | 6978 ( 11.7) | 1955 ( 12.7) | 3352 ( 17.5) | 1551 ( 32.6) |  |  |
| **Metabolic factors** |  |  |  |  |  |  |  |  |
| SBP, mmHg, (mean (SD)) |  | 128.72 (15.64) | 128.25 (15.48) | 128.39 (15.52) | 129.81 (15.92) | 131.20 (16.45) | <0.001 | 0.107 |
| BMI, kg/m2, (mean (SD)) |  | 26.83 (4.82) | 26.55 (4.70) | 26.93 (4.80) | 27.35 (5.00) | 28.03 (5.33) | <0.001 | 0.162 |
| Waist, cm, mean (SD) |  | 87.53 (13.17) | 87.05 (12.93) | 87.15 (13.17) | 88.29 (13.53) | 91.63 (13.75) | <0.001 | 0.185 |
| HbA1c, %, (mean (SD)) |  | 5.21 (0.33) | 5.19 (0.32) | 5.20 (0.33) | 5.22 (0.33) | 5.32 (0.36) | <0.001 | 0.194 |
| FPG, mmol/L, (mean (SD)) |  | 4.91 (0.50) | 4.91 (0.50) | 4.92 (0.51) | 4.93 (0.49) | 4.86 (0.49) | 0.116 | 0.069 |
| TC, mmol/L, (mean (SD)) |  | 5.49 (1.00) | 5.47 (1.00) | 5.49 (1.00) | 5.53 (1.00) | 5.58 (1.07) | <0.001 | 0.057 |
| HDL-C, mmol/L, (mean (SD)) |  | 1.41 (0.35) | 1.42 (0.35) | 1.42 (0.36) | 1.40 (0.36) | 1.32 (0.35) | <0.001 | 0.147 |
| LDL, mmol/L, (mean (SD)) |  | 3.43 (0.79) | 3.42 (0.79) | 3.42 (0.79) | 3.46 (0.79) | 3.53 (0.83) | <0.001 | 0.078 |
| TG, mmol/L, (median [IQR]) |  | 1.29 [0.90, 1.96] | 1.27 [0.89, 1.92] | 1.26 [0.89, 1.91] | 1.32 [0.93, 2.02] | 1.50 [1.02, 2.28] | <0.001 | 0.131 |
| **Comorbidities** |  |  |  |  |  |  |  |  |
| CVD, n (%) |  | 1652 ( 1.7) | 806 ( 1.3) | 245 ( 1.6) | 371 ( 1.9) | 230 ( 4.8) | <0.001 | 0.106 |
| CAD, n (%) |  | 1030 ( 1.0) | 495 ( 0.8) | 138 ( 0.9) | 221 ( 1.2) | 176 ( 3.7) | <0.001 | 0.101 |
| Stroke, n (%) |  | 641 ( 0.6) | 313 ( 0.5) | 105 ( 0.7) | 159 ( 0.8) | 64 ( 1.3) | <0.001 | 0.045 |
| HF n (%) |  | 102 ( 0.1) | 51 ( 0.1) | 16 ( 0.1) | 24 ( 0.1) | 11 ( 0.2) | 0.017 | 0.019 |

*Supplementary Table 3 presents the data distribution of participants born after 1960. SD, Standard deviation; SMD, Standardized mean difference; PRS, Polygenic risk score; T2DM, Type 2 diabetes mellitus; IQR, Interquartile range; BMI, Body mass index; HbA1c, Hemoglobin A1c (Glycated hemoglobin); SBP, Systolic blood pressure; TC, Total cholesterol; HDL-C, High-density lipoprotein cholesterol; LDL-C, Low-density lipoprotein cholesterol; CVD, Cardiovascular disease; CAD, Coronary artery disease; HF, Heart failure.*

Supplementary Table 4. Distribution of Education Level by Birth Year and Sex.

| Period | College education | High school | Middle school | Primary school or less | College education | High school | Middle school | Primary school or less | College education | High school | Middle school | Primary school or less |
| --- | --- | --- | --- | --- | --- | --- | --- | --- | --- | --- | --- | --- |
|  | Total | | | | Female | | | | Male | | | |
| ~1938 | 1629 (31.4%) | 919 (17.7%) | 718 (13.8%) | 1927 (37.1%) | 657 (25.0%) | 541 (20.6%) | 453 (17.2%) | 978 (37.2%) | 972 (37.9%) | 378 (14.7%) | 265 (10.3%) | 949 (37.0%) |
| 1939 | 3348 (31.8%) | 1903 (18.1%) | 1530 (14.6%) | 3732 (35.5%) | 1319 (23.9%) | 1166 (21.1%) | 997 (18.0%) | 2048 (37.0%) | 2029 (40.7%) | 737 (14.8%) | 533 (10.7%) | 1684 (33.8%) |
| 1940 | 4702 (33.2%) | 2596 (18.3%) | 2121 (15.0%) | 4758 (33.6%) | 1906 (25.7%) | 1572 (21.2%) | 1364 (18.4%) | 2584 (34.8%) | 2796 (41.4%) | 1024 (15.2%) | 757 (11.2%) | 2174 (32.2%) |
| 1941 | 5020 (33.6%) | 2732 (18.3%) | 2333 (15.6%) | 4852 (32.5%) | 2108 (26.9%) | 1637 (20.9%) | 1489 (19.0%) | 2611 (33.3%) | 2912 (41.1%) | 1095 (15.4%) | 844 (11.9%) | 2241 (31.6%) |
| 1942 | 6125 (34.8%) | 3253 (18.5%) | 2764 (15.7%) | 5448 (31.0%) | 2581 (28.1%) | 1917 (20.8%) | 1776 (19.3%) | 2926 (31.8%) | 3544 (42.2%) | 1336 (15.9%) | 988 (11.8%) | 2522 (30.1%) |
| 1943 | 7138 (37.0%) | 3567 (18.5%) | 3063 (15.9%) | 5528 (28.6%) | 3092 (30.2%) | 2059 (20.1%) | 2049 (20.0%) | 3034 (29.6%) | 4046 (44.6%) | 1508 (16.6%) | 1014 (11.2%) | 2494 (27.5%) |
| 1944 | 8236 (38.2%) | 4058 (18.8%) | 3551 (16.5%) | 5723 (26.5%) | 3538 (30.8%) | 2372 (20.6%) | 2334 (20.3%) | 3256 (28.3%) | 4698 (46.7%) | 1686 (16.7%) | 1217 (12.1%) | 2467 (24.5%) |
| 1945 | 7465 (39.7%) | 3547 (18.9%) | 3104 (16.5%) | 4699 (25.0%) | 3393 (32.6%) | 2116 (20.4%) | 2097 (20.2%) | 2787 (26.8%) | 4072 (48.3%) | 1431 (17.0%) | 1007 (12.0%) | 1912 (22.7%) |
| 1946 | 9589 (41.5%) | 4247 (18.4%) | 3944 (17.1%) | 5310 (23.0%) | 4524 (35.2%) | 2560 (19.9%) | 2654 (20.6%) | 3120 (24.3%) | 5065 (49.5%) | 1687 (16.5%) | 1290 (12.6%) | 2190 (21.4%) |
| 1947 | 11158 (43.8%) | 4624 (18.2%) | 4164 (16.4%) | 5508 (21.6%) | 5326 (37.4%) | 2778 (19.5%) | 2838 (19.9%) | 3286 (23.1%) | 5832 (52.0%) | 1846 (16.4%) | 1326 (11.8%) | 2222 (19.8%) |
| 1948 | 9931 (45.5%) | 4052 (18.6%) | 3601 (16.5%) | 4226 (19.4%) | 4807 (39.3%) | 2474 (20.2%) | 2477 (20.3%) | 2468 (20.2%) | 5124 (53.5%) | 1578 (16.5%) | 1124 (11.7%) | 1758 (18.3%) |
| 1949 | 9401 (48.7%) | 3460 (17.9%) | 3097 (16.1%) | 3333 (17.3%) | 4709 (43.0%) | 2130 (19.4%) | 2180 (19.9%) | 1935 (17.7%) | 4692 (56.3%) | 1330 (16.0%) | 917 (11.0%) | 1398 (16.8%) |
| 1950 | 8691 (50.3%) | 3211 (18.6%) | 2699 (15.6%) | 2689 (15.6%) | 4421 (45.2%) | 1958 (20.0%) | 1839 (18.8%) | 1573 (16.1%) | 4270 (56.9%) | 1253 (16.7%) | 860 (11.5%) | 1116 (14.9%) |
| 1951 | 8493 (51.5%) | 3054 (18.5%) | 2671 (16.2%) | 2268 (13.8%) | 4399 (47.3%) | 1851 (19.9%) | 1758 (18.9%) | 1289 (13.9%) | 4094 (56.9%) | 1203 (16.7%) | 913 (12.7%) | 979 (13.6%) |
| 1952 | 8420 (52.6%) | 2951 (18.4%) | 2545 (15.9%) | 2105 (13.1%) | 4492 (48.5%) | 1869 (20.2%) | 1655 (17.9%) | 1248 (13.5%) | 3928 (58.1%) | 1082 (16.0%) | 890 (13.2%) | 857 (12.7%) |
| 1953 | 8351 (53.7%) | 2803 (18.0%) | 2520 (16.2%) | 1869 (12.0%) | 4436 (50.1%) | 1738 (19.6%) | 1655 (18.7%) | 1027 (11.6%) | 3915 (58.5%) | 1065 (15.9%) | 865 (12.9%) | 842 (12.6%) |
| 1954 | 8181 (54.1%) | 2718 (18.0%) | 2417 (16.0%) | 1799 (11.9%) | 4445 (51.1%) | 1702 (19.6%) | 1585 (18.2%) | 968 (11.1%) | 3736 (58.2%) | 1016 (15.8%) | 832 (13.0%) | 831 (13.0%) |
| 1955 | 8003 (54.9%) | 2590 (17.8%) | 2354 (16.2%) | 1618 (11.1%) | 4342 (52.2%) | 1604 (19.3%) | 1498 (18.0%) | 877 (10.5%) | 3661 (58.6%) | 986 (15.8%) | 856 (13.7%) | 741 (11.9%) |
| 1956 | 7942 (55.9%) | 2475 (17.4%) | 2272 (16.0%) | 1517 (10.7%) | 4464 (53.8%) | 1531 (18.5%) | 1471 (17.7%) | 827 (10.0%) | 3478 (58.8%) | 944 (16.0%) | 801 (13.5%) | 690 (11.7%) |
| 1957 | 7983 (56.9%) | 2406 (17.2%) | 2244 (16.0%) | 1386 (9.9%) | 4378 (54.8%) | 1480 (18.5%) | 1412 (17.7%) | 723 (9.0%) | 3605 (59.8%) | 926 (15.4%) | 832 (13.8%) | 663 (11.0%) |
| 1958 | 7663 (56.5%) | 2349 (17.3%) | 2714 (20.0%) | 834 (6.2%) | 4158 (53.6%) | 1458 (18.8%) | 1740 (22.4%) | 402 (5.2%) | 3505 (60.4%) | 891 (15.4%) | 974 (16.8%) | 432 (7.4%) |
| 1959 | 7419 (56.9%) | 2216 (17.0%) | 2632 (20.2%) | 777 (6.0%) | 4003 (54.3%) | 1386 (18.8%) | 1637 (22.2%) | 342 (4.6%) | 3416 (60.2%) | 830 (14.6%) | 995 (17.5%) | 435 (7.7%) |
| 1960 | 7452 (57.7%) | 2134 (16.5%) | 2568 (19.9%) | 756 (5.9%) | 3962 (54.8%) | 1329 (18.4%) | 1579 (21.8%) | 366 (5.1%) | 3490 (61.5%) | 805 (14.2%) | 989 (17.4%) | 390 (6.9%) |
| 1961 | 7177 (58.7%) | 1905 (15.6%) | 2475 (20.2%) | 673 (5.5%) | 3854 (56.2%) | 1174 (17.1%) | 1501 (21.9%) | 323 (4.7%) | 3323 (61.8%) | 731 (13.6%) | 974 (18.1%) | 350 (6.5%) |
| 1962 | 7195 (58.9%) | 1901 (15.6%) | 2430 (19.9%) | 680 (5.6%) | 3928 (57.0%) | 1194 (17.3%) | 1465 (21.3%) | 303 (4.4%) | 3267 (61.5%) | 707 (13.3%) | 965 (18.2%) | 377 (7.1%) |
| 1963 | 7013 (59.4%) | 1934 (16.4%) | 2237 (18.9%) | 622 (5.3%) | 3843 (57.5%) | 1191 (17.8%) | 1372 (20.5%) | 275 (4.1%) | 3170 (61.9%) | 743 (14.5%) | 865 (16.9%) | 347 (6.8%) |
| 1964 | 7120 (61.0%) | 1785 (15.3%) | 2229 (19.1%) | 536 (4.6%) | 3779 (59.2%) | 1090 (17.1%) | 1278 (20.0%) | 232 (3.6%) | 3341 (63.1%) | 695 (13.1%) | 951 (18.0%) | 304 (5.7%) |
| 1965 | 6952 (61.2%) | 1758 (15.5%) | 2177 (19.2%) | 472 (4.2%) | 3649 (59.4%) | 1063 (17.3%) | 1229 (20.0%) | 198 (3.2%) | 3303 (63.3%) | 695 (13.3%) | 948 (18.2%) | 274 (5.2%) |
| 1966 | 6847 (62.4%) | 1675 (15.3%) | 2018 (18.4%) | 438 (4.0%) | 3635 (61.4%) | 1009 (17.0%) | 1079 (18.2%) | 197 (3.3%) | 3212 (63.5%) | 666 (13.2%) | 939 (18.6%) | 241 (4.8%) |
| 1967 | 5503 (61.8%) | 1363 (15.3%) | 1689 (19.0%) | 346 (3.9%) | 2875 (61.2%) | 781 (16.6%) | 898 (19.1%) | 146 (3.1%) | 2628 (62.6%) | 582 (13.9%) | 791 (18.8%) | 200 (4.8%) |
| 1968~ | 4539 (63.4%) | 984 (13.8%) | 1344 (18.8%) | 287 (4.0%) | 2380 (61.8%) | 591 (15.3%) | 750 (19.5%) | 130 (3.4%) | 2159 (65.4%) | 393 (11.9%) | 594 (18.0%) | 157 (4.8%) |

Supplementary Table 5. 5-Year and 10-Year Risk of T2DM Stratified by Birth Year.

| **Group** | **Total Population** | **5-Year Risk (%)** | **10-Year Risk (%)** |
| --- | --- | --- | --- |
| Total Population | **Education Level** |  |  |
|  | College Education | 0.71% (0.68%, 0.75%) | 2.2% (2.1%, 2.2%) |
|  | High School | 0.77% (0.71%, 0.83%) | 2.5% (2.4%, 2.6%) |
|  | Middle School | 0.82% (0.76%, 0.88%) | 2.7% (2.6%, 2.8%) |
|  | Primary School or Less | 1.7% (1.6%, 1.7%) | 5.0% (4.9%, 5.2%) |
| pre-1949 | **Education Level** |  |  |
|  | College Education | 0.99% (0.92%, 1.1%) | 2.9% (2.8%, 3.0%) |
|  | High School | 0.97% (0.87%, 1.1%) | 3.1% (2.9%, 3.3%) |
|  | Middle School | 1.0% (0.90%, 1.1%) | 3.3% (3.1%, 3.5%) |
|  | Primary School or Less | 1.7% (1.6%, 1.9%) | 5.2% (5.0%, 5.4%) |
| 1950-1959 | **Education Level** |  |  |
|  | College Education | 0.68% (0.63%, 0.74%) | 2.0% (1.9%, 2.1%) |
|  | High School | 0.71% (0.62%, 0.82%) | 2.2% (2.1%, 2.4%) |
|  | Middle School | 0.75% (0.65%, 0.86%) | 2.5% (2.3%, 2.7%) |
|  | Primary School or Less | 1.5% (1.3%, 1.7%) | 4.9% (4.5%, 5.2%) |
| post-1960 | **Education Level** |  |  |
|  | College Education | 0.38% (0.33%, 0.43%) | 1.3% (1.2%, 1.4%) |
|  | High School | 0.37% (0.28%, 0.48%) | 1.5% (1.3%, 1.7%) |
|  | Middle School | 0.58% (0.48%, 0.69%) | 2.0% (1.8%, 2.2%) |
|  | Primary School or Less | 1.2% (0.89%, 1.5%) | 4.1% (3.6%, 4.7%) |

Supplementary Table 6. Numbers at risk for incident T2DM by educational attainment and birth cohort.

| Group | Education | At 0 years | At 5 years | At 10 years | At 15 years |
| --- | --- | --- | --- | --- | --- |
| Total | College education | 224686 | 220393 | 212377 | 204082 |
|  | High school | 81170 | 79387 | 75933 | 72415 |
|  | Middle school | 78225 | 76524 | 73132 | 69744 |
|  | Primary school or less | 76716 | 73408 | 67280 | 61118 |
| pre-1949 | College education | 83742 | 81229 | 76479 | 71225 |
|  | High school | 38958 | 37761 | 35371 | 32892 |
|  | Middle school | 33990 | 32936 | 30847 | 28628 |
|  | Primary school or less | 55044 | 52391 | 47477 | 42395 |
| 1950-1959 | College education | 81146 | 79843 | 77534 | 75366 |
|  | High school | 26773 | 26313 | 25504 | 24743 |
|  | Middle school | 25068 | 24640 | 23772 | 23003 |
|  | Primary school or less | 16862 | 16313 | 15302 | 14373 |
| post-1960 | College education | 59798 | 59321 | 58364 | 57491 |
|  | High school | 15439 | 15313 | 15058 | 14780 |
|  | Middle school | 19167 | 18948 | 18513 | 18113 |
|  | Primary school or less | 4810 | 4704 | 4501 | 4350 |

Supplementary Table 7. Association between education level and risk of T2DM stratified by birth year and sex across progressively adjusted models.

| **Period** | **Subgroup** | **Level** | **Model 0** | **Model 1** | **Model 2** | **Model 3** | **Model 4** |
| --- | --- | --- | --- | --- | --- | --- | --- |
| Total | Overall | College Education | Ref | Ref | Ref | Ref | Ref |
|  |  | High School | 1.17 (1.12-1.22) | 1.16 (1.12-1.21) | 1.11 (1.07-1.16) | 1.10 (1.05-1.15) | 1.05 (1.00-1.10) |
|  |  | Middle School | 1.26 (1.21-1.31) | 1.30 (1.25-1.36) | 1.15 (1.11-1.20) | 1.13 (1.08-1.18) | 1.07 (1.02-1.12) |
|  |  | Primary School or Less | 2.32 (2.24-2.40) | 1.92 (1.85-1.99) | 1.47 (1.42-1.53) | 1.39 (1.34-1.44) | 1.22 (1.17-1.27) |
|  | Male | College Education | Ref | Ref | Ref | Ref | Ref |
|  |  | High School | 1.20 (1.14-1.27) | 1.15 (1.08-1.21) | 1.10 (1.04-1.16) | 1.07 (1.02-1.14) | 1.00 (0.95-1.06) |
|  |  | Middle School | 1.33 (1.26-1.41) | 1.32 (1.25-1.40) | 1.17 (1.10-1.24) | 1.14 (1.07-1.20) | 1.05 (0.99-1.11) |
|  |  | Primary School or Less | 2.25 (2.15-2.35) | 1.87 (1.78-1.96) | 1.43 (1.36-1.50) | 1.37 (1.30-1.44) | 1.17 (1.11-1.23) |
|  | Female | College Education | Ref | Ref | Ref | Ref | Ref |
|  |  | High School | 1.28 (1.20-1.36) | 1.18 (1.11-1.26) | 1.14 (1.07-1.22) | 1.13 (1.06-1.20) | 1.12 (1.05-1.20) |
|  |  | Middle School | 1.38 (1.30-1.47) | 1.29 (1.21-1.37) | 1.15 (1.08-1.23) | 1.12 (1.05-1.19) | 1.11 (1.03-1.18) |
|  |  | Primary School or Less | 2.57 (2.43-2.71) | 1.98 (1.87-2.10) | 1.53 (1.44-1.62) | 1.39 (1.31-1.48) | 1.30 (1.22-1.39) |
| pre-1949 | Overall | College Education | Ref | Ref | Ref | Ref | Ref |
|  |  | High School | 1.08 (1.02-1.14) | 1.14 (1.08-1.20) | 1.11 (1.05-1.17) | 1.09 (1.03-1.15) | 1.05 (0.99-1.12) |
|  |  | Middle School | 1.13 (1.07-1.20) | 1.23 (1.16-1.30) | 1.14 (1.07-1.21) | 1.11 (1.04-1.17) | 1.08 (1.01-1.15) |
|  |  | Primary School or Less | 1.79 (1.71-1.87) | 1.78 (1.70-1.86) | 1.46 (1.40-1.54) | 1.40 (1.33-1.46) | 1.24 (1.17-1.31) |
|  | Male | College Education | Ref | Ref | Ref | Ref | Ref |
|  |  | High School | 1.15 (1.07-1.24) | 1.14 (1.06-1.23) | 1.10 (1.02-1.19) | 1.08 (1.00-1.16) | 1.02 (0.94-1.10) |
|  |  | Middle School | 1.32 (1.22-1.43) | 1.30 (1.20-1.41) | 1.18 (1.09-1.28) | 1.13 (1.05-1.23) | 1.08 (0.99-1.17) |
|  |  | Primary School or Less | 1.84 (1.74-1.95) | 1.76 (1.66-1.86) | 1.41 (1.32-1.50) | 1.36 (1.28-1.45) | 1.17 (1.09-1.25) |
|  | Female | College Education | Ref | Ref | Ref | Ref | Ref |
|  |  | High School | 1.16 (1.07-1.27) | 1.13 (1.04-1.23) | 1.11 (1.02-1.21) | 1.09 (1.00-1.19) | 1.11 (1.01-1.22) |
|  |  | Middle School | 1.20 (1.10-1.31) | 1.17 (1.08-1.28) | 1.12 (1.03-1.22) | 1.09 (1.00-1.18) | 1.10 (1.00-1.21) |
|  |  | Primary School or Less | 1.94 (1.81-2.08) | 1.79 (1.67-1.92) | 1.53 (1.42-1.65) | 1.41 (1.31-1.52) | 1.33 (1.22-1.44) |
| 1950-1959 | Overall | College Education | Ref | Ref | Ref | Ref | Ref |
|  |  | High School | 1.09 (1.01-1.18) | 1.14 (1.06-1.23) | 1.08 (1.01-1.17) | 1.07 (0.99-1.15) | 1.02 (0.94-1.10) |
|  |  | Middle School | 1.22 (1.14-1.32) | 1.29 (1.20-1.39) | 1.11 (1.03-1.19) | 1.09 (1.01-1.17) | 1.03 (0.95-1.11) |
|  |  | Primary School or Less | 2.40 (2.25-2.57) | 2.22 (2.07-2.37) | 1.55 (1.45-1.67) | 1.45 (1.35-1.56) | 1.26 (1.17-1.36) |
|  | Male | College Education | Ref | Ref | Ref | Ref | Ref |
|  |  | High School | 1.11 (1.00-1.23) | 1.10 (0.99-1.22) | 1.03 (0.93-1.14) | 1.01 (0.91-1.12) | 0.94 (0.85-1.04) |
|  |  | Middle School | 1.29 (1.16-1.43) | 1.27 (1.15-1.41) | 1.09 (0.98-1.21) | 1.07 (0.97-1.19) | 0.98 (0.88-1.10) |
|  |  | Primary School or Less | 2.33 (2.14-2.55) | 2.16 (1.98-2.36) | 1.57 (1.42-1.72) | 1.49 (1.35-1.64) | 1.26 (1.13-1.39) |
|  | Female | College Education | Ref | Ref | Ref | Ref | Ref |
|  |  | High School | 1.20 (1.07-1.34) | 1.20 (1.07-1.34) | 1.16 (1.04-1.30) | 1.15 (1.03-1.29) | 1.14 (1.02-1.27) |
|  |  | Middle School | 1.33 (1.20-1.48) | 1.33 (1.19-1.47) | 1.13 (1.01-1.26) | 1.11 (0.99-1.23) | 1.09 (0.98-1.23) |
|  |  | Primary School or Less | 2.56 (2.31-2.84) | 2.32 (2.09-2.58) | 1.55 (1.38-1.73) | 1.39 (1.24-1.56) | 1.28 (1.13-1.44) |
| post-1960 | Overall | College Education | Ref | Ref | Ref | Ref | Ref |
|  |  | High School | 1.21 (1.08-1.35) | 1.26 (1.12-1.41) | 1.20 (1.07-1.35) | 1.19 (1.06-1.33) | 1.12 (0.99-1.27) |
|  |  | Middle School | 1.53 (1.39-1.69) | 1.54 (1.40-1.70) | 1.26 (1.14-1.39) | 1.23 (1.11-1.36) | 1.09 (0.98-1.21) |
|  |  | Primary School or Less | 2.93 (2.57-3.32) | 2.26 (1.98-2.58) | 1.34 (1.17-1.54) | 1.22 (1.06-1.40) | 1.10 (0.95-1.26) |
|  | Male | College Education | Ref | Ref | Ref | Ref | Ref |
|  |  | High School | 1.27 (1.09-1.48) | 1.28 (1.09-1.49) | 1.20 (1.02-1.40) | 1.18 (1.01-1.38) | 1.07 (0.89-1.28) |
|  |  | Middle School | 1.53 (1.34-1.74) | 1.54 (1.35-1.75) | 1.27 (1.11-1.45) | 1.25 (1.09-1.43) | 1.07 (0.92-1.24) |
|  |  | Primary School or Less | 2.55 (2.16-3.01) | 2.11 (1.78-2.49) | 1.34 (1.12-1.60) | 1.24 (1.04-1.48) | 1.06 (0.88-1.27) |
|  | Female | College Education | Ref | Ref | Ref | Ref | Ref |
|  |  | High School | 1.24 (1.05-1.46) | 1.24 (1.05-1.47) | 1.22 (1.03-1.44) | 1.21 (1.02-1.43) | 1.17 (0.99-1.40) |
|  |  | Middle School | 1.61 (1.40-1.86) | 1.55 (1.34-1.79) | 1.25 (1.08-1.45) | 1.21 (1.04-1.41) | 1.11 (0.94-1.30) |
|  |  | Primary School or Less | 3.33 (2.72-4.08) | 2.57 (2.08-3.17) | 1.34 (1.07-1.67) | 1.17 (0.94-1.46) | 1.16 (0.93-1.46) |

*Model 0 was unadjusted. Model 1 was adjusted for age, sex, ethnicity, and polygenic risk score for T2DM. Model 2 was additionally adjusted for socioeconomic factors: Townsend deprivation index, household income, marital status, and employment status. Model 3 was additionally adjusted for lifestyle factors: alcohol consumption, diet, sleep pattern, physical activity, and smoking status. Model 4 was additionally adjusted for metabolic factors: body mass index, HbA1c, HDL-C, systolic blood pressure, and triglycerides.*

Supplementary Table 8. Association between education level and T2DM risk stratified by birth year and sex, adjusted for distinct categories of covariates.

| **Period** | **Subgroup** | **Level** | **Unadjusted** | **Basic Adjustment** | **Socioeconomic Factors** | **Behavioral Factors** | **Metabolic Factors** |
| --- | --- | --- | --- | --- | --- | --- | --- |
| Total | Overall | College Education | Ref | Ref | Ref | Ref | Ref |
|  |  | High School | 1.17 (1.12-1.22) | 1.16 (1.12-1.21) | 1.11 (1.07-1.16) | 1.14 (1.09-1.18) | 1.08 (1.03-1.13) |
|  |  | Middle School | 1.26 (1.21-1.31) | 1.30 (1.25-1.36) | 1.15 (1.11-1.20) | 1.23 (1.18-1.29) | 1.15 (1.10-1.20) |
|  |  | Primary School or Less | 2.32 (2.24-2.40) | 1.92 (1.85-1.99) | 1.47 (1.42-1.53) | 1.68 (1.62-1.74) | 1.44 (1.38-1.50) |
|  | Male | College Education | Ref | Ref | Ref | Ref | Ref |
|  |  | High School | 1.20 (1.14-1.27) | 1.15 (1.08-1.21) | 1.10 (1.04-1.16) | 1.11 (1.05-1.18) | 1.04 (0.98-1.10) |
|  |  | Middle School | 1.33 (1.26-1.41) | 1.32 (1.25-1.40) | 1.17 (1.10-1.24) | 1.25 (1.18-1.33) | 1.14 (1.07-1.21) |
|  |  | Primary School or Less | 2.25 (2.15-2.35) | 1.87 (1.78-1.96) | 1.43 (1.36-1.50) | 1.67 (1.59-1.75) | 1.38 (1.31-1.45) |
|  | Female | College Education | Ref | Ref | Ref | Ref | Ref |
|  |  | High School | 1.28 (1.20-1.36) | 1.18 (1.11-1.26) | 1.14 (1.07-1.22) | 1.16 (1.09-1.23) | 1.14 (1.07-1.22) |
|  |  | Middle School | 1.38 (1.30-1.47) | 1.29 (1.21-1.37) | 1.15 (1.08-1.23) | 1.21 (1.14-1.29) | 1.18 (1.11-1.27) |
|  |  | Primary School or Less | 2.57 (2.43-2.71) | 1.98 (1.87-2.10) | 1.53 (1.44-1.62) | 1.67 (1.58-1.77) | 1.53 (1.43-1.63) |
| pre-1949 | Overall | College Education | Ref | Ref | Ref | Ref | Ref |
|  |  | High School | 1.08 (1.02-1.14) | 1.14 (1.08-1.20) | 1.11 (1.05-1.17) | 1.11 (1.05-1.18) | 1.08 (1.01-1.14) |
|  |  | Middle School | 1.13 (1.07-1.20) | 1.23 (1.16-1.30) | 1.14 (1.07-1.21) | 1.17 (1.11-1.24) | 1.13 (1.06-1.21) |
|  |  | Primary School or Less | 1.79 (1.71-1.87) | 1.78 (1.70-1.86) | 1.46 (1.40-1.54) | 1.62 (1.54-1.69) | 1.39 (1.32-1.47) |
|  | Male | College Education | Ref | Ref | Ref | Ref | Ref |
|  |  | High School | 1.15 (1.07-1.24) | 1.14 (1.06-1.23) | 1.10 (1.02-1.19) | 1.10 (1.02-1.19) | 1.05 (0.97-1.13) |
|  |  | Middle School | 1.32 (1.22-1.43) | 1.30 (1.20-1.41) | 1.18 (1.09-1.28) | 1.23 (1.13-1.33) | 1.15 (1.06-1.25) |
|  |  | Primary School or Less | 1.84 (1.74-1.95) | 1.76 (1.66-1.86) | 1.41 (1.32-1.50) | 1.62 (1.53-1.72) | 1.34 (1.26-1.42) |
|  | Female | College Education | Ref | Ref | Ref | Ref | Ref |
|  |  | High School | 1.16 (1.07-1.27) | 1.13 (1.04-1.23) | 1.11 (1.02-1.21) | 1.11 (1.02-1.20) | 1.12 (1.02-1.23) |
|  |  | Middle School | 1.20 (1.10-1.31) | 1.17 (1.08-1.28) | 1.12 (1.03-1.22) | 1.12 (1.03-1.22) | 1.14 (1.03-1.25) |
|  |  | Primary School or Less | 1.94 (1.81-2.08) | 1.79 (1.67-1.92) | 1.53 (1.42-1.65) | 1.58 (1.47-1.70) | 1.47 (1.35-1.60) |
| 1950-1959 | Overall | College Education | Ref | Ref | Ref | Ref | Ref |
|  |  | High School | 1.09 (1.01-1.18) | 1.14 (1.06-1.23) | 1.08 (1.01-1.17) | 1.11 (1.03-1.20) | 1.04 (0.97-1.12) |
|  |  | Middle School | 1.22 (1.14-1.32) | 1.29 (1.20-1.39) | 1.11 (1.03-1.19) | 1.22 (1.13-1.31) | 1.13 (1.05-1.22) |
|  |  | Primary School or Less | 2.40 (2.25-2.57) | 2.22 (2.07-2.37) | 1.55 (1.45-1.67) | 1.85 (1.72-1.98) | 1.57 (1.46-1.69) |
|  | Male | College Education | Ref | Ref | Ref | Ref | Ref |
|  |  | High School | 1.11 (1.00-1.23) | 1.10 (0.99-1.22) | 1.03 (0.93-1.14) | 1.06 (0.96-1.18) | 0.97 (0.87-1.08) |
|  |  | Middle School | 1.29 (1.16-1.43) | 1.27 (1.15-1.41) | 1.09 (0.98-1.21) | 1.20 (1.08-1.33) | 1.08 (0.97-1.21) |
|  |  | Primary School or Less | 2.33 (2.14-2.55) | 2.16 (1.98-2.36) | 1.57 (1.42-1.72) | 1.85 (1.69-2.03) | 1.54 (1.40-1.69) |
|  | Female | College Education | Ref | Ref | Ref | Ref | Ref |
|  |  | High School | 1.20 (1.07-1.34) | 1.20 (1.07-1.34) | 1.16 (1.04-1.30) | 1.18 (1.06-1.32) | 1.15 (1.02-1.28) |
|  |  | Middle School | 1.33 (1.20-1.48) | 1.33 (1.19-1.47) | 1.13 (1.01-1.26) | 1.24 (1.12-1.38) | 1.19 (1.06-1.33) |
|  |  | Primary School or Less | 2.56 (2.31-2.84) | 2.32 (2.09-2.58) | 1.55 (1.38-1.73) | 1.83 (1.64-2.04) | 1.63 (1.45-1.82) |
| post-1960 | Overall | College Education | Ref | Ref | Ref | Ref | Ref |
|  |  | High School | 1.21 (1.08-1.35) | 1.26 (1.12-1.41) | 1.20 (1.07-1.35) | 1.22 (1.09-1.37) | 1.15 (1.02-1.30) |
|  |  | Middle School | 1.53 (1.39-1.69) | 1.54 (1.40-1.70) | 1.26 (1.14-1.39) | 1.43 (1.29-1.57) | 1.23 (1.11-1.36) |
|  |  | Primary School or Less | 2.93 (2.57-3.32) | 2.26 (1.98-2.58) | 1.34 (1.17-1.54) | 1.71 (1.50-1.96) | 1.52 (1.33-1.74) |
|  | Male | College Education | Ref | Ref | Ref | Ref | Ref |
|  |  | High School | 1.27 (1.09-1.48) | 1.28 (1.09-1.49) | 1.20 (1.02-1.40) | 1.23 (1.05-1.44) | 1.12 (0.94-1.33) |
|  |  | Middle School | 1.53 (1.34-1.74) | 1.54 (1.35-1.75) | 1.27 (1.11-1.45) | 1.44 (1.26-1.65) | 1.21 (1.05-1.39) |
|  |  | Primary School or Less | 2.55 (2.16-3.01) | 2.11 (1.78-2.49) | 1.34 (1.12-1.60) | 1.68 (1.41-2.00) | 1.44 (1.21-1.72) |
|  | Female | College Education | Ref | Ref | Ref | Ref | Ref |
|  |  | High School | 1.24 (1.05-1.46) | 1.24 (1.05-1.47) | 1.22 (1.03-1.44) | 1.22 (1.03-1.44) | 1.17 (0.99-1.39) |
|  |  | Middle School | 1.61 (1.40-1.86) | 1.55 (1.34-1.79) | 1.25 (1.08-1.45) | 1.40 (1.21-1.62) | 1.25 (1.07-1.45) |
|  |  | Primary School or Less | 3.33 (2.72-4.08) | 2.57 (2.08-3.17) | 1.34 (1.07-1.67) | 1.76 (1.42-2.18) | 1.63 (1.32-2.01) |

*Unadjusted model included no covariates. Basic Adjustment model included age, sex, ethnicity, and T2DM PRS. Subsequent models were adjusted for variables in the Basic Adjustment model plus specific categories of covariates: Socioeconomic Factors (Townsend deprivation index, household income, employment, marriage); Behavioral Factors (alcohol consumption, diet, sleep pattern, physical activity, and smoking status); and Metabolic Factors (body mass index, HbA1c, HDL-C, systolic blood pressure, and triglycerides). Models were not adjusted for sex in sex-stratified analyses.*

Supplementary Table 9. P values for education-by-birth-cohort interaction corresponding to Supplementary Table 6.

| Subgroup | N | T2DM_events | Competing_deaths | Model 0 | Model 1 | Model 2 | Model 3 | Model 4 |
| --- | --- | --- | --- | --- | --- | --- | --- | --- |
| Overall | 460,797 | 19,931 | 33,637 | <0.001 | <0.001 | 0.013 | 0.012 | 0.160 |
| Male | 205,485 | 11,175 | 18,942 | <0.001 | <0.001 | 0.074 | 0.082 | 0.147 |
| Female | 255,312 | 8,756 | 14,695 | <0.001 | <0.001 | 0.470 | 0.426 | 0.970 |

P values were obtained from likelihood-ratio tests comparing pooled Fine-Gray models with and without education-by-birth-cohort interaction terms.

Supplementary Table 10. P values for education-by-birth-cohort interaction corresponding to Supplementary Table 7.

| Subgroup | N | T2DM_events | Competing_deaths | Unadjusted | Basic Adjustment | Socioeconomic Factors | Behavioral Factors | Metabolic Factors |
| --- | --- | --- | --- | --- | --- | --- | --- | --- |
| Overall | 460,797 | 19,931 | 33,637 | <0.001 | <0.001 | 0.013 | <0.001 | 0.002 |
| Male | 205,485 | 11,175 | 18,942 | <0.001 | <0.001 | 0.074 | <0.001 | 0.012 |
| Female | 255,312 | 8,756 | 14,695 | <0.001 | <0.001 | 0.470 | 0.007 | 0.586 |

P values were obtained from likelihood-ratio tests comparing pooled Fine-Gray models with and without education-by-birth-cohort interaction terms.

Supplementary Table 11. Cause-specific Cox analysis of the association between educational attainment and incident T2DM.

| Group | Model | Level | Result | P_value |
| --- | --- | --- | --- | --- |
| Total | Model 0 | College education | Ref | NA |
|  |  | High school | 1.18 (1.13-1.23) | <0.001 |
|  |  | Middle school | 1.26 (1.21-1.31) | <0.001 |
|  |  | Primary school or less | 2.40 (2.32-2.49) | <0.001 |
|  | Model 1 | College education | Ref | NA |
|  |  | High school | 1.16 (1.12-1.21) | <0.001 |
|  |  | Middle school | 1.30 (1.25-1.36) | <0.001 |
|  |  | Primary school or less | 1.96 (1.89-2.03) | <0.001 |
|  | Model 2 | College education | Ref | NA |
|  |  | High school | 1.11 (1.07-1.16) | <0.001 |
|  |  | Middle school | 1.15 (1.11-1.20) | <0.001 |
|  |  | Primary school or less | 1.49 (1.43-1.54) | <0.001 |
|  | Model 3 | College education | Ref | NA |
|  |  | High school | 1.10 (1.05-1.14) | <0.001 |
|  |  | Middle school | 1.12 (1.08-1.17) | <0.001 |
|  |  | Primary school or less | 1.40 (1.35-1.45) | <0.001 |
|  | Model 4 | College education | Ref | NA |
|  |  | High school | 1.05 (1.00-1.09) | 0.03 |
|  |  | Middle school | 1.07 (1.03-1.12) | <0.001 |
|  |  | Primary school or less | 1.22 (1.18-1.27) | <0.001 |
| pre-1949 | Model 0 | College education | Ref | NA |
|  |  | High school | 1.08 (1.02-1.14) | <0.001 |
|  |  | Middle school | 1.14 (1.07-1.20) | <0.001 |
|  |  | Primary school or less | 1.84 (1.76-1.92) | <0.001 |
|  | Model 1 | College education | Ref | NA |
|  |  | High school | 1.14 (1.08-1.21) | <0.001 |
|  |  | Middle school | 1.24 (1.17-1.31) | <0.001 |
|  |  | Primary school or less | 1.82 (1.74-1.90) | <0.001 |
|  | Model 2 | College education | Ref | NA |
|  |  | High school | 1.11 (1.05-1.17) | <0.001 |
|  |  | Middle school | 1.14 (1.08-1.21) | <0.001 |
|  |  | Primary school or less | 1.48 (1.41-1.55) | <0.001 |
|  | Model 3 | College education | Ref | NA |
|  |  | High school | 1.09 (1.03-1.15) | <0.001 |
|  |  | Middle school | 1.11 (1.04-1.17) | <0.001 |
|  |  | Primary school or less | 1.41 (1.34-1.47) | <0.001 |
|  | Model 4 | College education | Ref | NA |
|  |  | High school | 1.05 (1.00-1.11) | 0.07 |
|  |  | Middle school | 1.08 (1.02-1.15) | 0.01 |
|  |  | Primary school or less | 1.24 (1.19-1.30) | <0.001 |
| 1950-1959 | Model 0 | College education | Ref | NA |
|  |  | High school | 1.09 (1.02-1.18) | 0.02 |
|  |  | Middle school | 1.23 (1.14-1.32) | <0.001 |
|  |  | Primary school or less | 2.45 (2.29-2.62) | <0.001 |
|  | Model 1 | College education | Ref | NA |
|  |  | High school | 1.14 (1.06-1.23) | <0.001 |
|  |  | Middle school | 1.29 (1.20-1.39) | <0.001 |
|  |  | Primary school or less | 2.26 (2.11-2.41) | <0.001 |
|  | Model 2 | College education | Ref | NA |
|  |  | High school | 1.08 (1.00-1.16) | 0.04 |
|  |  | Middle school | 1.10 (1.02-1.19) | 0.01 |
|  |  | Primary school or less | 1.55 (1.45-1.67) | <0.001 |
|  | Model 3 | College education | Ref | NA |
|  |  | High school | 1.07 (0.99-1.15) | 0.09 |
|  |  | Middle school | 1.08 (1.00-1.17) | 0.04 |
|  |  | Primary school or less | 1.45 (1.34-1.56) | <0.001 |
|  | Model 4 | College education | Ref | NA |
|  |  | High school | 1.01 (0.94-1.09) | 0.73 |
|  |  | Middle school | 1.03 (0.96-1.11) | 0.43 |
|  |  | Primary school or less | 1.25 (1.17-1.35) | <0.001 |
| post-1960 | Model 0 | College education | Ref | NA |
|  |  | High school | 1.21 (1.08-1.35) | <0.001 |
|  |  | Middle school | 1.54 (1.39-1.69) | <0.001 |
|  |  | Primary school or less | 2.96 (2.60-3.36) | <0.001 |
|  | Model 1 | College education | Ref | NA |
|  |  | High school | 1.25 (1.12-1.40) | <0.001 |
|  |  | Middle school | 1.53 (1.39-1.69) | <0.001 |
|  |  | Primary school or less | 2.33 (2.05-2.65) | <0.001 |
|  | Model 2 | College education | Ref | NA |
|  |  | High school | 1.20 (1.07-1.35) | <0.001 |
|  |  | Middle school | 1.26 (1.14-1.39) | <0.001 |
|  |  | Primary school or less | 1.35 (1.18-1.54) | <0.001 |
|  | Model 3 | College education | Ref | NA |
|  |  | High school | 1.19 (1.06-1.33) | <0.001 |
|  |  | Middle school | 1.23 (1.12-1.36) | <0.001 |
|  |  | Primary school or less | 1.22 (1.06-1.40) | <0.001 |
|  | Model 4 | College education | Ref | NA |
|  |  | High school | 1.12 (1.00-1.25) | 0.06 |
|  |  | Middle school | 1.10 (1.00-1.22) | 0.06 |
|  |  | Primary school or less | 1.09 (0.95-1.25) | 0.20 |
| Male | Model 0 | College education | Ref | NA |
|  |  | High school | 1.21 (1.15-1.28) | <0.001 |
|  |  | Middle school | 1.34 (1.27-1.42) | <0.001 |
|  |  | Primary school or less | 2.37 (2.26-2.48) | <0.001 |
|  | Model 1 | College education | Ref | NA |
|  |  | High school | 1.15 (1.09-1.22) | <0.001 |
|  |  | Middle school | 1.33 (1.26-1.41) | <0.001 |
|  |  | Primary school or less | 1.93 (1.84-2.02) | <0.001 |
|  | Model 2 | College education | Ref | NA |
|  |  | High school | 1.10 (1.04-1.16) | <0.001 |
|  |  | Middle school | 1.17 (1.10-1.24) | <0.001 |
|  |  | Primary school or less | 1.45 (1.38-1.52) | <0.001 |
|  | Model 3 | College education | Ref | NA |
|  |  | High school | 1.07 (1.01-1.14) | 0.01 |
|  |  | Middle school | 1.13 (1.07-1.20) | <0.001 |
|  |  | Primary school or less | 1.38 (1.32-1.46) | <0.001 |
|  | Model 4 | College education | Ref | NA |
|  |  | High school | 1.00 (0.95-1.06) | 0.96 |
|  |  | Middle school | 1.05 (1.00-1.12) | 0.07 |
|  |  | Primary school or less | 1.18 (1.12-1.24) | <0.001 |
| Female | Model 0 | College education | Ref | NA |
|  |  | High school | 1.29 (1.21-1.37) | <0.001 |
|  |  | Middle school | 1.39 (1.31-1.48) | <0.001 |
|  |  | Primary school or less | 2.63 (2.50-2.78) | <0.001 |
|  | Model 1 | College education | Ref | NA |
|  |  | High school | 1.18 (1.11-1.26) | <0.001 |
|  |  | Middle school | 1.28 (1.21-1.36) | <0.001 |
|  |  | Primary school or less | 2.00 (1.89-2.12) | <0.001 |
|  | Model 2 | College education | Ref | NA |
|  |  | High school | 1.14 (1.07-1.21) | <0.001 |
|  |  | Middle school | 1.15 (1.08-1.22) | <0.001 |
|  |  | Primary school or less | 1.53 (1.45-1.62) | <0.001 |
|  | Model 3 | College education | Ref | NA |
|  |  | High school | 1.12 (1.06-1.20) | <0.001 |
|  |  | Middle school | 1.11 (1.05-1.18) | <0.001 |
|  |  | Primary school or less | 1.39 (1.32-1.48) | <0.001 |
|  | Model 4 | College education | Ref | NA |
|  |  | High school | 1.12 (1.05-1.19) | <0.001 |
|  |  | Middle school | 1.10 (1.04-1.17) | <0.001 |
|  |  | Primary school or less | 1.30 (1.22-1.37) | <0.001 |

Supplementary Table 12. Proportional hazards assumption tests for the cause-specific Cox models.

| Group | Model | Term | PH_chisq | PH_df | PH_P_value |
| --- | --- | --- | --- | --- | --- |
| Total | Model 0 | education_level | 4.31 | 3.00 | 0.23 |
|  |  | GLOBAL | 4.31 | 3.00 | 0.23 |
|  | Model 1 | education_level | 3.97 | 3.00 | 0.26 |
|  |  | age | 7.85 | 1.00 | 0.01 |
|  |  | sex | 2.01 | 1.00 | 0.16 |
|  |  | race2 | 0.01 | 1.00 | 0.93 |
|  |  | PRS_T2DM_z | 5.02 | 1.00 | 0.03 |
|  |  | GLOBAL | 19.88 | 7.00 | 0.01 |
|  | Model 2 | education_level | 3.71 | 3.00 | 0.29 |
|  |  | age | 8.27 | 1.00 | 0.00 |
|  |  | sex | 1.31 | 1.00 | 0.25 |
|  |  | race2 | 0.02 | 1.00 | 0.90 |
|  |  | PRS_T2DM_z | 4.71 | 1.00 | 0.03 |
|  |  | TDI | 1.03 | 1.00 | 0.31 |
|  |  | income_i0 | 4.17 | 4.00 | 0.38 |
|  |  | marriage | 2.88 | 1.00 | 0.09 |
|  |  | employment | 21.34 | 2.00 | 0.00 |
|  |  | GLOBAL | 53.49 | 15.00 | 0.00 |
|  | Model 3 | education_level | 3.62 | 3.00 | 0.31 |
|  |  | age | 7.40 | 1.00 | 0.01 |
|  |  | sex | 1.09 | 1.00 | 0.30 |
|  |  | race2 | 0.05 | 1.00 | 0.83 |
|  |  | PRS_T2DM_z | 4.40 | 1.00 | 0.04 |
|  |  | TDI | 0.62 | 1.00 | 0.43 |
|  |  | income_i0 | 4.45 | 4.00 | 0.35 |
|  |  | marriage | 2.87 | 1.00 | 0.09 |
|  |  | employment | 18.44 | 2.00 | 0.00 |
|  |  | drinking_status | 3.96 | 3.00 | 0.27 |
|  |  | diet_2group | 1.71 | 1.00 | 0.19 |
|  |  | sleep_group | 9.86 | 2.00 | 0.01 |
|  |  | MET_level | 17.12 | 2.00 | 0.00 |
|  |  | smoke_i0 | 28.42 | 2.00 | 0.00 |
|  |  | GLOBAL | 110.32 | 25.00 | 0.00 |
|  | Model 4 | education_level | 5.30 | 3.00 | 0.15 |
|  |  | age | 4.60 | 1.00 | 0.03 |
|  |  | sex | 0.91 | 1.00 | 0.34 |
|  |  | race2 | 0.03 | 1.00 | 0.86 |
|  |  | PRS_T2DM_z | 0.27 | 1.00 | 0.60 |
|  |  | TDI | 0.12 | 1.00 | 0.73 |
|  |  | income_i0 | 11.70 | 4.00 | 0.02 |
|  |  | marriage | 3.25 | 1.00 | 0.07 |
|  |  | employment | 7.38 | 2.00 | 0.03 |
|  |  | drinking_status | 5.82 | 3.00 | 0.12 |
|  |  | diet_2group | 3.44 | 1.00 | 0.06 |
|  |  | sleep_group | 5.16 | 2.00 | 0.08 |
|  |  | MET_level | 14.66 | 2.00 | 0.00 |
|  |  | smoke_i0 | 24.07 | 2.00 | 0.00 |
|  |  | BMI_i0 | 10.76 | 1.00 | 0.00 |
|  |  | HBA1C | 55.45 | 1.00 | 0.00 |
|  |  | HDL_i0 | 0.01 | 1.00 | 0.93 |
|  |  | SBP_i0 | 4.98 | 1.00 | 0.03 |
|  |  | TG_i0 | 1.54 | 1.00 | 0.22 |
|  |  | GLOBAL | 157.30 | 30.00 | 0.00 |
| pre-1949 | Model 0 | education_level | 3.96 | 3.00 | 0.27 |
|  |  | GLOBAL | 3.96 | 3.00 | 0.27 |
|  | Model 1 | education_level | 3.76 | 3.00 | 0.29 |
|  |  | age | 1.79 | 1.00 | 0.18 |
|  |  | sex | 3.24 | 1.00 | 0.07 |
|  |  | race2 | 3.38 | 1.00 | 0.07 |
|  |  | PRS_T2DM_z | 10.71 | 1.00 | 0.00 |
|  |  | GLOBAL | 21.91 | 7.00 | 0.00 |
|  | Model 2 | education_level | 3.57 | 3.00 | 0.31 |
|  |  | age | 2.19 | 1.00 | 0.14 |
|  |  | sex | 2.54 | 1.00 | 0.11 |
|  |  | race2 | 3.29 | 1.00 | 0.07 |
|  |  | PRS_T2DM_z | 10.27 | 1.00 | 0.00 |
|  |  | TDI | 10.04 | 1.00 | 0.00 |
|  |  | income_i0 | 1.31 | 4.00 | 0.86 |
|  |  | marriage | 0.46 | 1.00 | 0.50 |
|  |  | employment | 2.96 | 2.00 | 0.23 |
|  |  | GLOBAL | 37.87 | 15.00 | 0.00 |
|  | Model 3 | education_level | 3.41 | 3.00 | 0.33 |
|  |  | age | 2.01 | 1.00 | 0.16 |
|  |  | sex | 2.26 | 1.00 | 0.13 |
|  |  | race2 | 3.48 | 1.00 | 0.06 |
|  |  | PRS_T2DM_z | 9.93 | 1.00 | 0.00 |
|  |  | TDI | 8.61 | 1.00 | 0.00 |
|  |  | income_i0 | 1.64 | 4.00 | 0.80 |
|  |  | marriage | 0.50 | 1.00 | 0.48 |
|  |  | employment | 2.15 | 2.00 | 0.34 |
|  |  | drinking_status | 9.22 | 3.00 | 0.03 |
|  |  | diet_2group | 0.03 | 1.00 | 0.86 |
|  |  | sleep_group | 16.14 | 2.00 | 0.00 |
|  |  | MET_level | 10.06 | 2.00 | 0.01 |
|  |  | smoke_i0 | 18.45 | 2.00 | 0.00 |
|  |  | GLOBAL | 88.29 | 25.00 | 0.00 |
|  | Model 4 | education_level | 3.18 | 3.00 | 0.36 |
|  |  | age | 1.29 | 1.00 | 0.26 |
|  |  | sex | 0.93 | 1.00 | 0.34 |
|  |  | race2 | 2.17 | 1.00 | 0.14 |
|  |  | PRS_T2DM_z | 4.57 | 1.00 | 0.03 |
|  |  | TDI | 4.61 | 1.00 | 0.03 |
|  |  | income_i0 | 6.05 | 4.00 | 0.20 |
|  |  | marriage | 0.84 | 1.00 | 0.36 |
|  |  | employment | 0.70 | 2.00 | 0.70 |
|  |  | drinking_status | 7.94 | 3.00 | 0.05 |
|  |  | diet_2group | 0.83 | 1.00 | 0.36 |
|  |  | sleep_group | 11.06 | 2.00 | 0.00 |
|  |  | MET_level | 8.59 | 2.00 | 0.01 |
|  |  | smoke_i0 | 12.32 | 2.00 | 0.00 |
|  |  | BMI_i0 | 10.56 | 1.00 | 0.00 |
|  |  | HBA1C | 6.79 | 1.00 | 0.01 |
|  |  | HDL_i0 | 1.09 | 1.00 | 0.30 |
|  |  | SBP_i0 | 2.33 | 1.00 | 0.13 |
|  |  | TG_i0 | 0.22 | 1.00 | 0.64 |
|  |  | GLOBAL | 83.79 | 30.00 | 0.00 |
| 1950-1959 | Model 0 | education_level | 0.62 | 3.00 | 0.89 |
|  |  | GLOBAL | 0.62 | 3.00 | 0.89 |
|  | Model 1 | education_level | 0.95 | 3.00 | 0.81 |
|  |  | age | 5.69 | 1.00 | 0.02 |
|  |  | sex | 0.27 | 1.00 | 0.60 |
|  |  | race2 | 3.21 | 1.00 | 0.07 |
|  |  | PRS_T2DM_z | 0.26 | 1.00 | 0.61 |
|  |  | GLOBAL | 10.11 | 7.00 | 0.18 |
|  | Model 2 | education_level | 1.26 | 3.00 | 0.74 |
|  |  | age | 5.62 | 1.00 | 0.02 |
|  |  | sex | 0.39 | 1.00 | 0.53 |
|  |  | race2 | 3.04 | 1.00 | 0.08 |
|  |  | PRS_T2DM_z | 0.29 | 1.00 | 0.59 |
|  |  | TDI | 1.36 | 1.00 | 0.24 |
|  |  | income_i0 | 12.80 | 4.00 | 0.01 |
|  |  | marriage | 0.42 | 1.00 | 0.52 |
|  |  | employment | 7.48 | 2.00 | 0.02 |
|  |  | GLOBAL | 33.18 | 15.00 | 0.00 |
|  | Model 3 | education_level | 1.58 | 3.00 | 0.66 |
|  |  | age | 5.68 | 1.00 | 0.02 |
|  |  | sex | 0.47 | 1.00 | 0.49 |
|  |  | race2 | 2.99 | 1.00 | 0.08 |
|  |  | PRS_T2DM_z | 0.35 | 1.00 | 0.55 |
|  |  | TDI | 1.64 | 1.00 | 0.20 |
|  |  | income_i0 | 12.94 | 4.00 | 0.01 |
|  |  | marriage | 0.44 | 1.00 | 0.51 |
|  |  | employment | 6.45 | 2.00 | 0.04 |
|  |  | drinking_status | 6.70 | 3.00 | 0.08 |
|  |  | diet_2group | 1.43 | 1.00 | 0.23 |
|  |  | sleep_group | 0.70 | 2.00 | 0.71 |
|  |  | MET_level | 4.70 | 2.00 | 0.10 |
|  |  | smoke_i0 | 8.47 | 2.00 | 0.01 |
|  |  | GLOBAL | 51.39 | 25.00 | 0.00 |
|  | Model 4 | education_level | 4.43 | 3.00 | 0.22 |
|  |  | age | 6.38 | 1.00 | 0.01 |
|  |  | sex | 0.32 | 1.00 | 0.57 |
|  |  | race2 | 4.63 | 1.00 | 0.03 |
|  |  | PRS_T2DM_z | 2.61 | 1.00 | 0.11 |
|  |  | TDI | 4.55 | 1.00 | 0.03 |
|  |  | income_i0 | 16.33 | 4.00 | 0.00 |
|  |  | marriage | 0.20 | 1.00 | 0.65 |
|  |  | employment | 2.01 | 2.00 | 0.37 |
|  |  | drinking_status | 10.46 | 3.00 | 0.02 |
|  |  | diet_2group | 1.57 | 1.00 | 0.21 |
|  |  | sleep_group | 1.68 | 2.00 | 0.43 |
|  |  | MET_level | 5.29 | 2.00 | 0.07 |
|  |  | smoke_i0 | 8.96 | 2.00 | 0.01 |
|  |  | BMI_i0 | 2.04 | 1.00 | 0.15 |
|  |  | HBA1C | 30.80 | 1.00 | 0.00 |
|  |  | HDL_i0 | 2.22 | 1.00 | 0.14 |
|  |  | SBP_i0 | 0.84 | 1.00 | 0.36 |
|  |  | TG_i0 | 4.33 | 1.00 | 0.04 |
|  |  | GLOBAL | 113.91 | 30.00 | 0.00 |
| post-1960 | Model 0 | education_level | 5.94 | 3.00 | 0.11 |
|  |  | GLOBAL | 5.94 | 3.00 | 0.11 |
|  | Model 1 | education_level | 5.61 | 3.00 | 0.13 |
|  |  | age | 12.20 | 1.00 | 0.00 |
|  |  | sex | 0.86 | 1.00 | 0.35 |
|  |  | race2 | 0.69 | 1.00 | 0.40 |
|  |  | PRS_T2DM_z | 0.37 | 1.00 | 0.54 |
|  |  | GLOBAL | 19.14 | 7.00 | 0.01 |
|  | Model 2 | education_level | 5.42 | 3.00 | 0.14 |
|  |  | age | 11.97 | 1.00 | 0.00 |
|  |  | sex | 0.75 | 1.00 | 0.39 |
|  |  | race2 | 0.72 | 1.00 | 0.40 |
|  |  | PRS_T2DM_z | 0.34 | 1.00 | 0.56 |
|  |  | TDI | 0.64 | 1.00 | 0.42 |
|  |  | income_i0 | 3.07 | 4.00 | 0.55 |
|  |  | marriage | 1.87 | 1.00 | 0.17 |
|  |  | employment | 14.29 | 2.00 | 0.00 |
|  |  | GLOBAL | 39.90 | 15.00 | 0.00 |
|  | Model 3 | education_level | 5.20 | 3.00 | 0.16 |
|  |  | age | 11.92 | 1.00 | 0.00 |
|  |  | sex | 0.73 | 1.00 | 0.39 |
|  |  | race2 | 0.71 | 1.00 | 0.40 |
|  |  | PRS_T2DM_z | 0.28 | 1.00 | 0.59 |
|  |  | TDI | 0.73 | 1.00 | 0.39 |
|  |  | income_i0 | 2.85 | 4.00 | 0.58 |
|  |  | marriage | 1.80 | 1.00 | 0.18 |
|  |  | employment | 13.56 | 2.00 | 0.00 |
|  |  | drinking_status | 0.98 | 3.00 | 0.81 |
|  |  | diet_2group | 1.06 | 1.00 | 0.30 |
|  |  | sleep_group | 2.17 | 2.00 | 0.34 |
|  |  | MET_level | 3.19 | 2.00 | 0.20 |
|  |  | smoke_i0 | 1.81 | 2.00 | 0.41 |
|  |  | GLOBAL | 47.61 | 25.00 | 0.00 |
|  | Model 4 | education_level | 3.95 | 3.00 | 0.27 |
|  |  | age | 10.39 | 1.00 | 0.00 |
|  |  | sex | 1.72 | 1.00 | 0.19 |
|  |  | race2 | 0.69 | 1.00 | 0.41 |
|  |  | PRS_T2DM_z | 0.10 | 1.00 | 0.76 |
|  |  | TDI | 2.11 | 1.00 | 0.15 |
|  |  | income_i0 | 1.20 | 4.00 | 0.88 |
|  |  | marriage | 2.82 | 1.00 | 0.09 |
|  |  | employment | 8.87 | 2.00 | 0.01 |
|  |  | drinking_status | 0.85 | 3.00 | 0.84 |
|  |  | diet_2group | 1.36 | 1.00 | 0.24 |
|  |  | sleep_group | 1.43 | 2.00 | 0.49 |
|  |  | MET_level | 1.94 | 2.00 | 0.38 |
|  |  | smoke_i0 | 3.20 | 2.00 | 0.20 |
|  |  | BMI_i0 | 0.90 | 1.00 | 0.34 |
|  |  | HBA1C | 37.81 | 1.00 | 0.00 |
|  |  | HDL_i0 | 0.82 | 1.00 | 0.37 |
|  |  | SBP_i0 | 2.13 | 1.00 | 0.14 |
|  |  | TG_i0 | 1.42 | 1.00 | 0.23 |
|  |  | GLOBAL | 83.26 | 30.00 | 0.00 |
| Male | Model 0 | education_level | 1.53 | 3.00 | 0.68 |
|  |  | GLOBAL | 1.53 | 3.00 | 0.68 |
|  | Model 1 | education_level | 1.55 | 3.00 | 0.67 |
|  |  | age | 5.58 | 1.00 | 0.02 |
|  |  | race2 | 1.55 | 1.00 | 0.21 |
|  |  | PRS_T2DM_z | 8.10 | 1.00 | 0.00 |
|  |  | GLOBAL | 17.68 | 6.00 | 0.01 |
|  | Model 2 | education_level | 1.55 | 3.00 | 0.67 |
|  |  | age | 6.07 | 1.00 | 0.01 |
|  |  | race2 | 1.45 | 1.00 | 0.23 |
|  |  | PRS_T2DM_z | 7.77 | 1.00 | 0.01 |
|  |  | TDI | 0.25 | 1.00 | 0.62 |
|  |  | income_i0 | 3.88 | 4.00 | 0.42 |
|  |  | marriage | 4.16 | 1.00 | 0.04 |
|  |  | employment | 6.81 | 2.00 | 0.03 |
|  |  | GLOBAL | 38.24 | 14.00 | 0.00 |
|  | Model 3 | education_level | 1.62 | 3.00 | 0.66 |
|  |  | age | 5.43 | 1.00 | 0.02 |
|  |  | race2 | 1.24 | 1.00 | 0.27 |
|  |  | PRS_T2DM_z | 7.51 | 1.00 | 0.01 |
|  |  | TDI | 0.42 | 1.00 | 0.52 |
|  |  | income_i0 | 4.46 | 4.00 | 0.35 |
|  |  | marriage | 4.24 | 1.00 | 0.04 |
|  |  | employment | 5.57 | 2.00 | 0.06 |
|  |  | drinking_status | 4.90 | 3.00 | 0.18 |
|  |  | diet_2group | 1.90 | 1.00 | 0.17 |
|  |  | sleep_group | 3.88 | 2.00 | 0.14 |
|  |  | MET_level | 12.95 | 2.00 | 0.00 |
|  |  | smoke_i0 | 18.36 | 2.00 | 0.00 |
|  |  | GLOBAL | 74.39 | 24.00 | 0.00 |
|  | Model 4 | education_level | 4.85 | 3.00 | 0.18 |
|  |  | age | 2.06 | 1.00 | 0.15 |
|  |  | race2 | 0.84 | 1.00 | 0.36 |
|  |  | PRS_T2DM_z | 2.22 | 1.00 | 0.14 |
|  |  | TDI | 1.78 | 1.00 | 0.18 |
|  |  | income_i0 | 12.19 | 4.00 | 0.02 |
|  |  | marriage | 4.39 | 1.00 | 0.04 |
|  |  | employment | 1.70 | 2.00 | 0.43 |
|  |  | drinking_status | 6.41 | 3.00 | 0.09 |
|  |  | diet_2group | 2.99 | 1.00 | 0.08 |
|  |  | sleep_group | 2.20 | 2.00 | 0.33 |
|  |  | MET_level | 14.64 | 2.00 | 0.00 |
|  |  | smoke_i0 | 14.94 | 2.00 | 0.00 |
|  |  | BMI_i0 | 2.87 | 1.00 | 0.09 |
|  |  | HBA1C | 28.12 | 1.00 | 0.00 |
|  |  | HDL_i0 | 3.62 | 1.00 | 0.06 |
|  |  | SBP_i0 | 1.55 | 1.00 | 0.21 |
|  |  | TG_i0 | 0.36 | 1.00 | 0.55 |
|  |  | GLOBAL | 108.95 | 29.00 | 0.00 |
| Female | Model 0 | education_level | 2.32 | 3.00 | 0.51 |
|  |  | GLOBAL | 2.32 | 3.00 | 0.51 |
|  | Model 1 | education_level | 2.23 | 3.00 | 0.53 |
|  |  | age | 2.43 | 1.00 | 0.12 |
|  |  | race2 | 2.45 | 1.00 | 0.12 |
|  |  | PRS_T2DM_z | 0.10 | 1.00 | 0.75 |
|  |  | GLOBAL | 8.11 | 6.00 | 0.23 |
|  | Model 2 | education_level | 2.06 | 3.00 | 0.56 |
|  |  | age | 2.59 | 1.00 | 0.11 |
|  |  | race2 | 2.42 | 1.00 | 0.12 |
|  |  | PRS_T2DM_z | 0.07 | 1.00 | 0.79 |
|  |  | TDI | 5.20 | 1.00 | 0.02 |
|  |  | income_i0 | 3.62 | 4.00 | 0.46 |
|  |  | marriage | 0.12 | 1.00 | 0.73 |
|  |  | employment | 18.05 | 2.00 | 0.00 |
|  |  | GLOBAL | 30.59 | 14.00 | 0.01 |
|  | Model 3 | education_level | 1.83 | 3.00 | 0.61 |
|  |  | age | 2.29 | 1.00 | 0.13 |
|  |  | race2 | 2.60 | 1.00 | 0.11 |
|  |  | PRS_T2DM_z | 0.04 | 1.00 | 0.83 |
|  |  | TDI | 4.44 | 1.00 | 0.04 |
|  |  | income_i0 | 3.33 | 4.00 | 0.50 |
|  |  | marriage | 0.11 | 1.00 | 0.74 |
|  |  | employment | 16.50 | 2.00 | 0.00 |
|  |  | drinking_status | 1.41 | 3.00 | 0.70 |
|  |  | diet_2group | 0.43 | 1.00 | 0.51 |
|  |  | sleep_group | 6.75 | 2.00 | 0.03 |
|  |  | MET_level | 5.67 | 2.00 | 0.06 |
|  |  | smoke_i0 | 8.92 | 2.00 | 0.01 |
|  |  | GLOBAL | 51.57 | 24.00 | 0.00 |
|  | Model 4 | education_level | 0.84 | 3.00 | 0.84 |
|  |  | age | 2.85 | 1.00 | 0.09 |
|  |  | race2 | 0.63 | 1.00 | 0.43 |
|  |  | PRS_T2DM_z | 0.64 | 1.00 | 0.43 |
|  |  | TDI | 0.90 | 1.00 | 0.34 |
|  |  | income_i0 | 2.99 | 4.00 | 0.56 |
|  |  | marriage | 0.22 | 1.00 | 0.64 |
|  |  | employment | 7.88 | 2.00 | 0.02 |
|  |  | drinking_status | 0.58 | 3.00 | 0.90 |
|  |  | diet_2group | 0.96 | 1.00 | 0.33 |
|  |  | sleep_group | 4.33 | 2.00 | 0.11 |
|  |  | MET_level | 2.58 | 2.00 | 0.28 |
|  |  | smoke_i0 | 9.47 | 2.00 | 0.01 |
|  |  | BMI_i0 | 7.94 | 1.00 | 0.00 |
|  |  | HBA1C | 32.66 | 1.00 | 0.00 |
|  |  | HDL_i0 | 2.53 | 1.00 | 0.11 |
|  |  | SBP_i0 | 3.23 | 1.00 | 0.07 |
|  |  | TG_i0 | 1.53 | 1.00 | 0.22 |
|  |  | GLOBAL | 72.25 | 29.00 | 0.00 |

Supplementary Table 13. 10-year, 20-year, and 30-year risk of T2DM starting at age 30.

| **Group** | **Total Population** | **10-Year Risk (Age 40)** | **20-Year Risk (Age 50)** | **30-Year Risk (Age 60)** |
| --- | --- | --- | --- | --- |
| Total Population | **Education Level** |  |  |  |
|  | College Education | 0.086% (0.074%, 0.098%) | 0.692% (0.658%, 0.726%) | 2.744% (2.676%, 2.811%) |
|  | High School | 0.068% (0.050%, 0.086%) | 0.616% (0.563%, 0.669%) | 2.734% (2.623%, 2.845%) |
|  | Middle School | 0.107% (0.084%, 0.129%) | 0.866% (0.802%, 0.930%) | 3.201% (3.079%, 3.323%) |
|  | Primary School or Less | 0.069% (0.051%, 0.087%) | 0.727% (0.669%, 0.785%) | 3.581% (3.453%, 3.709%) |
| pre-1949 | **Education Level** |  |  |  |
|  | College Education | 0.018% (0.009%, 0.027%) | 0.175% (0.147%, 0.203%) | 1.704% (1.618%, 1.789%) |
|  | High School | 0.024% (0.009%, 0.039%) | 0.160% (0.122%, 0.199%) | 1.717% (1.591%, 1.842%) |
|  | Middle School | 0.033% (0.015%, 0.052%) | 0.159% (0.118%, 0.200%) | 1.636% (1.505%, 1.768%) |
|  | Primary School or Less | 0.027% (0.014%, 0.040%) | 0.236% (0.197%, 0.275%) | 2.193% (2.075%, 2.310%) |
| 1950-1959 | **Education Level** |  |  |  |
|  | College Education | 0.064% (0.046%, 0.081%) | 0.682% (0.626%, 0.739%) | 3.275% (3.153%, 3.397%) |
|  | High School | 0.067% (0.036%, 0.098%) | 0.809% (0.702%, 0.916%) | 3.496% (3.277%, 3.715%) |
|  | Middle School | 0.067% (0.035%, 0.099%) | 0.906% (0.789%, 1.023%) | 3.999% (3.758%, 4.241%) |
|  | Primary School or Less | 0.088% (0.044%, 0.133%) | 1.413% (1.235%, 1.590%) | 6.528% (6.156%, 6.899%) |
| post-1960 | **Education Level** |  |  |  |
|  | College Education | 0.215% (0.178%, 0.252%) | 1.459% (1.363%, 1.555%) | 3.494% (3.346%, 3.642%) |
|  | High School | 0.186% (0.118%, 0.253%) | 1.485% (1.296%, 1.675%) | 4.066% (3.752%, 4.379%) |
|  | Middle School | 0.295% (0.218%, 0.371%) | 2.124% (1.921%, 2.327%) | 5.019% (4.707%, 5.329%) |
|  | Primary School or Less | 0.506% (0.308%, 0.703%) | 4.289% (3.722%, 4.852%) | 10.200% (9.345%, 11.048%) |

Supplementary Table 14. Association between education level and risk of cardiovascular disease starting at age 30.

| **Period** | **Subgroup** | **Level** | **Unadjusted** | **Basic Adjustment** |
| --- | --- | --- | --- | --- |
| Total | Overall | College Education | Ref | Ref |
|  |  | High School | 1.00 (0.95-1.05) | 1.07 (1.02-1.12) |
|  |  | Middle School | 1.17 (1.12-1.22) | 1.25 (1.19-1.30) |
|  |  | Primary School or Less | 1.31 (1.25-1.37) | 1.28 (1.23-1.34) |
|  | Male | College Education | Ref | Ref |
|  |  | High School | 1.12 (1.06-1.20) | 1.13 (1.06-1.20) |
|  |  | Middle School | 1.39 (1.31-1.48) | 1.35 (1.27-1.43) |
|  |  | Primary School or Less | 1.39 (1.31-1.47) | 1.32 (1.25-1.40) |
|  | Female | College Education | Ref | Ref |
|  |  | High School | 0.97 (0.90-1.04) | 0.99 (0.92-1.07) |
|  |  | Middle School | 1.13 (1.05-1.21) | 1.13 (1.05-1.21) |
|  |  | Primary School or Less | 1.27 (1.19-1.37) | 1.22 (1.14-1.31) |
| pre-1949 | Overall | College Education | Ref | Ref |
|  |  | High School | 1.01 (0.92-1.10) | 1.10 (1.01-1.21) |
|  |  | Middle School | 0.96 (0.87-1.05) | 1.08 (0.98-1.19) |
|  |  | Primary School or Less | 1.29 (1.20-1.39) | 1.30 (1.21-1.40) |
|  | Male | College Education | Ref | Ref |
|  |  | High School | 1.14 (1.02-1.28) | 1.13 (1.01-1.27) |
|  |  | Middle School | 1.21 (1.07-1.38) | 1.17 (1.03-1.32) |
|  |  | Primary School or Less | 1.38 (1.26-1.52) | 1.30 (1.18-1.42) |
|  | Female | College Education | Ref | Ref |
|  |  | High School | 1.07 (0.92-1.24) | 1.05 (0.90-1.21) |
|  |  | Middle School | 0.99 (0.85-1.15) | 0.97 (0.84-1.13) |
|  |  | Primary School or Less | 1.39 (1.22-1.57) | 1.29 (1.13-1.46) |
| 1950-1959 | Overall | College Education | Ref | Ref |
|  |  | High School | 1.07 (0.99-1.15) | 1.12 (1.04-1.21) |
|  |  | Middle School | 1.23 (1.14-1.32) | 1.28 (1.19-1.37) |
|  |  | Primary School or Less | 2.03 (1.89-2.17) | 1.83 (1.71-1.97) |
|  | Male | College Education | Ref | Ref |
|  |  | High School | 1.16 (1.05-1.29) | 1.15 (1.04-1.27) |
|  |  | Middle School | 1.41 (1.28-1.55) | 1.36 (1.23-1.50) |
|  |  | Primary School or Less | 2.03 (1.86-2.23) | 1.83 (1.67-2.01) |
|  | Female | College Education | Ref | Ref |
|  |  | High School | 1.08 (0.97-1.21) | 1.09 (0.97-1.22) |
|  |  | Middle School | 1.22 (1.10-1.36) | 1.19 (1.07-1.32) |
|  |  | Primary School or Less | 2.07 (1.86-2.31) | 1.83 (1.64-2.05) |
| post-1960 | Overall | College Education | Ref | Ref |
|  |  | High School | 1.17 (1.07-1.28) | 1.22 (1.12-1.34) |
|  |  | Middle School | 1.45 (1.34-1.57) | 1.46 (1.35-1.57) |
|  |  | Primary School or Less | 3.03 (2.74-3.34) | 2.32 (2.10-2.56) |
|  | Male | College Education | Ref | Ref |
|  |  | High School | 1.31 (1.16-1.48) | 1.32 (1.17-1.49) |
|  |  | Middle School | 1.49 (1.34-1.65) | 1.47 (1.33-1.64) |
|  |  | Primary School or Less | 2.66 (2.35-3.03) | 2.20 (1.93-2.50) |
|  | Female | College Education | Ref | Ref |
|  |  | High School | 1.11 (0.97-1.27) | 1.13 (0.99-1.29) |
|  |  | Middle School | 1.48 (1.32-1.66) | 1.44 (1.28-1.61) |
|  |  | Primary School or Less | 3.40 (2.91-3.97) | 2.55 (2.18-2.98) |

*The Basic Adjustment model was adjusted for age, sex, ethnicity, and polygenic risk score for type 2 diabetes.*

Supplementary Table 15. Association between educational attainment and incident T2DM using attained age as the time scale with delayed entry

| Group | Model | Level | Result | P_value | p for interaction of birth period * education level |
| --- | --- | --- | --- | --- | --- |
| Total | Model 0 | College education | Ref |  | <0.001 |
|  |  | High school | 1.12 (1.07-1.17) | <0.001 |  |
|  |  | Middle school | 1.23 (1.19-1.29) | <0.001 |  |
|  |  | Primary school or less | 2.08 (2.01-2.15) | <0.001 |  |
|  | Model 1 | College education | Ref |  | <0.001 |
|  |  | High school | 1.18 (1.13-1.23) | <0.001 |  |
|  |  | Middle school | 1.32 (1.26-1.37) | <0.001 |  |
|  |  | Primary school or less | 2.06 (1.99-2.13) | <0.001 |  |
|  | Model 2 | College education | Ref |  | 0.033 |
|  |  | High school | 1.12 (1.08-1.17) | <0.001 |  |
|  |  | Middle school | 1.15 (1.11-1.20) | <0.001 |  |
|  |  | Primary school or less | 1.52 (1.47-1.58) | <0.001 |  |
|  | Model 3 | College education | Ref |  | 0.015 |
|  |  | High school | 1.10 (1.06-1.15) | <0.001 |  |
|  |  | Middle school | 1.13 (1.08-1.17) | <0.001 |  |
|  |  | Primary school or less | 1.43 (1.38-1.49) | <0.001 |  |
|  | Model 4 | College education | Ref |  | 0.136 |
|  |  | High school | 1.04 (1.00-1.09) | 0.05 |  |
|  |  | Middle school | 1.07 (1.03-1.12) | <0.001 |  |
|  |  | Primary school or less | 1.20 (1.16-1.25) | <0.001 |  |
| pre-1949 | Model 0 | College education | Ref |  |  |
|  |  | High school | 1.08 (1.02-1.14) | 0.01 |  |
|  |  | Middle school | 1.13 (1.07-1.20) | <0.001 |  |
|  |  | Primary school or less | 1.81 (1.74-1.90) | <0.001 |  |
|  | Model 1 | College education | Ref |  |  |
|  |  | High school | 1.15 (1.09-1.22) | <0.001 |  |
|  |  | Middle school | 1.24 (1.17-1.32) | <0.001 |  |
|  |  | Primary school or less | 1.86 (1.78-1.94) | <0.001 |  |
|  | Model 2 | College education | Ref |  |  |
|  |  | High school | 1.11 (1.05-1.18) | <0.001 |  |
|  |  | Middle school | 1.14 (1.08-1.21) | <0.001 |  |
|  |  | Primary school or less | 1.50 (1.43-1.57) | <0.001 |  |
|  | Model 3 | College education | Ref |  |  |
|  |  | High school | 1.09 (1.03-1.16) | <0.001 |  |
|  |  | Middle school | 1.11 (1.04-1.17) | <0.001 |  |
|  |  | Primary school or less | 1.42 (1.36-1.49) | <0.001 |  |
|  | Model 4 | College education | Ref |  |  |
|  |  | High school | 1.05 (0.99-1.11) | 0.09 |  |
|  |  | Middle school | 1.07 (1.01-1.14) | 0.02 |  |
|  |  | Primary school or less | 1.23 (1.17-1.29) | <0.001 |  |
| 1950-1959 | Model 0 | College education | Ref |  |  |
|  |  | High school | 1.09 (1.02-1.18) | 0.02 |  |
|  |  | Middle school | 1.23 (1.14-1.33) | <0.001 |  |
|  |  | Primary school or less | 2.43 (2.27-2.59) | <0.001 |  |
|  | Model 1 | College education | Ref |  |  |
|  |  | High school | 1.15 (1.06-1.24) | <0.001 |  |
|  |  | Middle school | 1.29 (1.20-1.39) | <0.001 |  |
|  |  | Primary school or less | 2.28 (2.13-2.44) | <0.001 |  |
|  | Model 2 | College education | Ref |  |  |
|  |  | High school | 1.08 (1.00-1.17) | 0.04 |  |
|  |  | Middle school | 1.10 (1.02-1.18) | 0.02 |  |
|  |  | Primary school or less | 1.56 (1.45-1.68) | <0.001 |  |
|  | Model 3 | College education | Ref |  |  |
|  |  | High school | 1.07 (0.99-1.15) | 0.08 |  |
|  |  | Middle school | 1.08 (1.00-1.16) | 0.04 |  |
|  |  | Primary school or less | 1.45 (1.35-1.56) | <0.001 |  |
|  | Model 4 | College education | Ref |  |  |
|  |  | High school | 1.01 (0.94-1.09) | 0.75 |  |
|  |  | Middle school | 1.04 (0.96-1.12) | 0.36 |  |
|  |  | Primary school or less | 1.23 (1.15-1.33) | <0.001 |  |
| post-1960 | Model 0 | College education | Ref |  |  |
|  |  | High school | 1.20 (1.07-1.35) | <0.001 |  |
|  |  | Middle school | 1.53 (1.39-1.69) | <0.001 |  |
|  |  | Primary school or less | 2.94 (2.58-3.34) | <0.001 |  |
|  | Model 1 | College education | Ref |  |  |
|  |  | High school | 1.26 (1.12-1.41) | <0.001 |  |
|  |  | Middle school | 1.55 (1.40-1.70) | <0.001 |  |
|  |  | Primary school or less | 2.35 (2.07-2.68) | <0.001 |  |
|  | Model 2 | College education | Ref |  |  |
|  |  | High school | 1.20 (1.07-1.35) | <0.001 |  |
|  |  | Middle school | 1.26 (1.14-1.39) | <0.001 |  |
|  |  | Primary school or less | 1.36 (1.19-1.56) | <0.001 |  |
|  | Model 3 | College education | Ref |  |  |
|  |  | High school | 1.19 (1.06-1.34) | <0.001 |  |
|  |  | Middle school | 1.24 (1.12-1.37) | <0.001 |  |
|  |  | Primary school or less | 1.23 (1.08-1.41) | <0.001 |  |
|  | Model 4 | College education | Ref |  |  |
|  |  | High school | 1.11 (0.99-1.25) | 0.07 |  |
|  |  | Middle school | 1.10 (1.00-1.22) | 0.06 |  |
|  |  | Primary school or less | 1.08 (0.94-1.23) | 0.29 |  |

*Model 0 was unadjusted. Model 1 was adjusted for age, sex, ethnicity, and polygenic risk score for T2DM. Model 2 was additionally adjusted for socioeconomic factors: Townsend deprivation index, household income, marital status, and employment status. Model 3 was additionally adjusted for lifestyle factors: alcohol consumption, diet, sleep pattern, physical activity, and smoking status. Model 4 was additionally adjusted for metabolic factors: body mass index, HbA1c, HDL-C, systolic blood pressure, and triglycerides. P for interaction was derived from a likelihood-ratio test comparing pooled attained-age Cox models with and without the education-by-birth-cohort interaction term.*

Supplementary Table 16. Education-related Relative Index of Inequality (RII) in risk of T2DM stratified by birth year and sex across progressively adjusted models.

| **Period** | **Subgroup** | **Model 0** | **Model 1** | **Model 2** | **Model 3** | **Model 4** |
| --- | --- | --- | --- | --- | --- | --- |
| Total | Overall | 2.47 (2.34-2.60) | 2.37 (2.25-2.50) | 1.66 (1.57-1.75) | 1.54 (1.46-1.62) | 1.29 (1.22-1.37) |
|  | Male | 2.48 (2.31-2.65) | 2.31 (2.16-2.47) | 1.61 (1.50-1.72) | 1.51 (1.41-1.62) | 1.21 (1.12-1.30) |
|  | Female | 2.88 (2.65-3.13) | 2.46 (2.27-2.68) | 1.73 (1.59-1.88) | 1.54 (1.41-1.67) | 1.41 (1.29-1.55) |
| pre-1949 | Overall | 2.31 (2.16-2.47) | 2.31 (2.16-2.47) | 1.73 (1.61-1.86) | 1.61 (1.50-1.73) | 1.36 (1.26-1.47) |
|  | Male | 2.44 (2.24-2.66) | 2.28 (2.09-2.49) | 1.64 (1.49-1.80) | 1.55 (1.41-1.71) | 1.26 (1.14-1.39) |
|  | Female | 2.65 (2.37-2.95) | 2.36 (2.12-2.64) | 1.85 (1.66-2.07) | 1.65 (1.47-1.84) | 1.50 (1.33-1.69) |
| 1950-1959 | Overall | 2.59 (2.35-2.87) | 2.53 (2.29-2.79) | 1.62 (1.47-1.79) | 1.50 (1.35-1.66) | 1.27 (1.14-1.41) |
|  | Male | 2.67 (2.35-3.04) | 2.46 (2.16-2.80) | 1.62 (1.42-1.85) | 1.52 (1.33-1.74) | 1.22 (1.06-1.40) |
|  | Female | 2.89 (2.48-3.37) | 2.64 (2.27-3.07) | 1.64 (1.40-1.91) | 1.46 (1.25-1.70) | 1.35 (1.15-1.58) |
| post-1960 | Overall | 2.80 (2.41-3.26) | 2.53 (2.18-2.93) | 1.53 (1.32-1.78) | 1.42 (1.22-1.65) | 1.18 (1.01-1.38) |
|  | Male | 2.72 (2.24-3.31) | 2.46 (2.03-2.99) | 1.55 (1.27-1.89) | 1.45 (1.18-1.77) | 1.12 (0.91-1.38) |
|  | Female | 3.05 (2.42-3.85) | 2.63 (2.09-3.31) | 1.53 (1.22-1.92) | 1.38 (1.10-1.74) | 1.25 (0.98-1.59) |

*Model 0 was unadjusted. Model 1 was adjusted for age, sex, ethnicity, and polygenic risk score for T2DM. Model 2 was additionally adjusted for socioeconomic factors: Townsend deprivation index, household income, marital status, and employment status. Model 3 was additionally adjusted for lifestyle factors: alcohol consumption, diet, sleep pattern, physical activity, and smoking status. Model 4 was additionally adjusted for metabolic factors: body mass index, HbA1c, HDL-C, systolic blood pressure, and triglycerides.*

Supplementary Table 17. Education-related Relative Index of Inequality (RII) in risk of T2DM stratified by birth year and sex, adjusting separately for distinct categories of covariates.

| **Period** | **Subgroup** | **Unadjusted** | **Basic Adjustment** | **Socioeconomic Factors** | **Behavioral Factors** | **Metabolic Factors** |
| --- | --- | --- | --- | --- | --- | --- |
| Total | Overall | 2.47 (2.34-2.60) | 2.37 (2.25-2.50) | 1.66 (1.57-1.75) | 1.99 (1.89-2.10) | 1.62 (1.53-1.72) |
|  | Male | 2.48 (2.31-2.65) | 2.31 (2.16-2.47) | 1.61 (1.50-1.72) | 1.98 (1.85-2.12) | 1.53 (1.42-1.64) |
|  | Female | 2.88 (2.65-3.13) | 2.46 (2.27-2.68) | 1.73 (1.59-1.88) | 1.97 (1.81-2.14) | 1.76 (1.60-1.93) |
| pre-1949 | Overall | 2.31 (2.16-2.47) | 2.31 (2.16-2.47) | 1.73 (1.61-1.86) | 2.01 (1.87-2.15) | 1.62 (1.50-1.76) |
|  | Male | 2.44 (2.24-2.66) | 2.28 (2.09-2.49) | 1.64 (1.49-1.80) | 2.02 (1.85-2.21) | 1.53 (1.40-1.68) |
|  | Female | 2.65 (2.37-2.95) | 2.36 (2.12-2.64) | 1.85 (1.66-2.07) | 1.96 (1.75-2.19) | 1.75 (1.54-1.98) |
| 1950-1959 | Overall | 2.59 (2.35-2.87) | 2.53 (2.29-2.79) | 1.62 (1.47-1.79) | 2.04 (1.84-2.25) | 1.68 (1.52-1.85) |
|  | Male | 2.67 (2.35-3.04) | 2.46 (2.16-2.80) | 1.62 (1.42-1.85) | 2.03 (1.78-2.32) | 1.60 (1.40-1.82) |
|  | Female | 2.89 (2.48-3.37) | 2.64 (2.27-3.07) | 1.64 (1.40-1.91) | 2.03 (1.74-2.36) | 1.79 (1.53-2.09) |
| post-1960 | Overall | 2.80 (2.41-3.26) | 2.53 (2.18-2.93) | 1.53 (1.32-1.78) | 2.00 (1.72-2.31) | 1.60 (1.38-1.86) |
|  | Male | 2.72 (2.24-3.31) | 2.46 (2.03-2.99) | 1.55 (1.27-1.89) | 2.01 (1.65-2.44) | 1.53 (1.25-1.87) |
|  | Female | 3.05 (2.42-3.85) | 2.63 (2.09-3.31) | 1.53 (1.22-1.92) | 1.96 (1.56-2.46) | 1.67 (1.32-2.11) |

*Unadjusted model included no covariates. Basic Adjustment model included age, sex, ethnicity, and T2DM PRS. Subsequent models were adjusted for variables in the Basic Adjustment model plus specific categories of covariates: Socioeconomic Factors (Townsend deprivation index, household income, employment, marriage); Behavioral Factors (alcohol consumption, diet, sleep pattern, physical activity, and smoking status); and Metabolic Factors (body mass index, HbA1c, HDL-C, systolic blood pressure, and triglycerides). Models were not adjusted for sex in sex-stratified analyses.*

Supplementary Table 18. Absolute educational inequalities in 10-year risk of incident T2DM.

| **Birth cohort** | **N** | **College education 10-year risk, %** | **Primary school or less 10-year risk, %** | **Standardized risk difference, percentage points** | **Excess T2DM events, n** | **SII, percentage points** |
| --- | --- | --- | --- | --- | --- | --- |
| **Total** | **460,797** | **2.20** | **4.78** | **2.57** | **11,862** | **3.03** |
| **pre-1949** | **211,734** | **2.77** | **5.20** | **2.43** | **5,154** | **3.34** |
| **1950-1959** | **149,849** | **2.00** | **4.77** | **2.77** | **4,144** | **2.59** |
| **post-1960** | **99,214** | **1.31** | **3.80** | **2.49** | **2,468** | **1.83** |

*Risks were directly standardized by age group, sex, and ethnicity. The standardized risk difference compares participants with primary school education or less with those with college education. Excess T2DM events were calculated as the standardized risk difference multiplied by the number of participants in the corresponding analytical group. SII denotes the slope index of inequality.*

Supplementary Table 19. Education-related Relative Index of Inequality (RII) in risk of T2DM starting at age 30.

| **Period** | **Subgroup** | **Unadjusted** | **Basic Adjustment** |
| --- | --- | --- | --- |
| Total | Overall | 1.99 (1.87-2.11) | 1.93 (1.82-2.05) |
|  | Male | 2.24 (2.07-2.42) | 2.01 (1.86-2.17) |
|  | Female | 2.01 (1.82-2.22) | 1.81 (1.64-2.00) |
| pre-1949 | Overall | 1.40 (1.25-1.56) | 1.45 (1.30-1.61) |
|  | Male | 1.61 (1.41-1.84) | 1.47 (1.28-1.68) |
|  | Female | 1.56 (1.29-1.88) | 1.41 (1.16-1.70) |
| 1950-1959 | Overall | 2.16 (1.96-2.38) | 2.06 (1.87-2.26) |
|  | Male | 2.43 (2.15-2.75) | 2.16 (1.91-2.45) |
|  | Female | 2.16 (1.86-2.51) | 1.93 (1.66-2.24) |
| post-1960 | Overall | 2.71 (2.42-3.04) | 2.43 (2.17-2.72) |
|  | Male | 2.76 (2.38-3.21) | 2.46 (2.12-2.86) |
|  | Female | 2.77 (2.32-3.31) | 2.39 (2.01-2.85) |

*The Basic Adjustment model was adjusted for sex, ethnicity, and polygenic risk score for type 2 diabetes.*

Supplementary Table 20. Hazard ratios for transitions between health, T2DM, and death according to education level in a multi-state model.

| Transition | Period | Unadjusted | | | Basic Adjusted | | |
| --- | --- | --- | --- | --- | --- | --- | --- |
|  |  | Education | HR (95% CI) | P value | Education | HR (95% CI) | P value |
| Baseline to T2DM | Total | College Education | ref | – | College Education | ref | – |
|  |  | High school | 1.18 (1.13-1.23) | <0.001 | High school | 1.16 (1.12-1.21) | <0.001 |
|  |  | Middle school | 1.26 (1.21-1.31) | <0.001 | Middle school | 1.30 (1.25-1.36) | <0.001 |
|  |  | Primary school or less | 2.40 (2.32-2.49) | <0.001 | Primary school or less | 1.96 (1.89-2.03) | <0.001 |
|  | pre-1949 | College Education | ref | - | College Education | ref | - |
|  |  | High school | 1.08 (1.02-1.14) | 0.005 | High school | 1.14 (1.08-1.21) | <0.001 |
|  |  | Middle school | 1.14 (1.07-1.20) | <0.001 | Middle school | 1.24 (1.17-1.31) | <0.001 |
|  |  | Primary school or less | 1.84 (1.76-1.92) | <0.001 | Primary school or less | 1.82 (1.74-1.90) | <0.001 |
|  | 1950-1959 | College Education | ref | - | College Education | ref | - |
|  |  | High school | 1.09 (1.02-1.18) | 0.018 | High school | 1.14 (1.06-1.23) | <0.001 |
|  |  | Middle school | 1.23 (1.14-1.32) | <0.001 | Middle school | 1.29 (1.20-1.39) | <0.001 |
|  |  | Primary school or less | 2.45 (2.29-2.62) | <0.001 | Primary school or less | 2.26 (2.11-2.41) | <0.001 |
|  | post-1960 | College Education | ref | - | College Education | ref | - |
|  |  | High school | 1.21 (1.08-1.35) | 0.001 | High school | 1.25 (1.12-1.40) | <0.001 |
|  |  | Middle school | 1.54 (1.39-1.69) | <0.001 | Middle school | 1.53 (1.39-1.69) | <0.001 |
|  |  | Primary school or less | 2.96 (2.60-3.36) | <0.001 | Primary school or less | 2.33 (2.05-2.65) | <0.001 |
| T2DM to Death | Total | College Education | ref | – | College Education | ref | – |
|  |  | High school | 1.19 (1.16-1.23) | <0.001 | High school | 1.07 (1.04-1.11) | <0.001 |
|  |  | Middle school | 1.15 (1.11-1.19) | <0.001 | Middle school | 1.15 (1.11-1.19) | <0.001 |
|  |  | Primary school or less | 2.33 (2.27-2.39) | <0.001 | Primary school or less | 1.53 (1.49-1.57) | <0.001 |
|  | pre-1949 | College Education | ref | - | College Education | ref | - |
|  |  | High school | 1.03 (0.99-1.07) | 0.094 | High school | 1.07 (1.03-1.11) | <0.001 |
|  |  | Middle school | 1.03 (0.99-1.07) | 0.184 | Middle school | 1.12 (1.08-1.16) | <0.001 |
|  |  | Primary school or less | 1.51 (1.47-1.56) | <0.001 | Primary school or less | 1.45 (1.41-1.50) | <0.001 |
|  | 1950-1959 | College Education | ref | - | College Education | ref | - |
|  |  | High school | 1.04 (0.97-1.12) | 0.242 | High school | 1.07 (1.00-1.14) | 0.067 |
|  |  | Middle school | 1.11 (1.03-1.19) | 0.004 | Middle school | 1.17 (1.09-1.26) | <0.001 |
|  |  | Primary school or less | 1.92 (1.80-2.05) | <0.001 | Primary school or less | 1.86 (1.74-1.98) | <0.001 |
|  | post-1960 | College Education | ref | - | College Education | ref | - |
|  |  | High school | 0.99 (0.86-1.13) | 0.87 | High school | 1.00 (0.88-1.15) | 0.96 |
|  |  | Middle school | 1.31 (1.18-1.47) | <0.001 | Middle school | 1.32 (1.18-1.47) | <0.001 |
|  |  | Primary school or less | 2.08 (1.77-2.44) | <0.001 | Primary school or less | 1.99 (1.69-2.34) | <0.001 |
| Baseline to Death | Total | College Education | ref | – | College Education | ref | – |
|  |  | High school | 1.00 (0.89-1.12) | 0.992 | High school | 0.92 (0.82-1.03) | 0.159 |
|  |  | Middle school | 1.05 (0.94-1.17) | 0.392 | Middle school | 1.06 (0.95-1.19) | 0.286 |
|  |  | Primary school or less | 1.61 (1.49-1.75) | <0.001 | Primary school or less | 1.29 (1.19-1.41) | <0.001 |
|  | pre-1949 | College Education | ref | - | College Education | ref | - |
|  |  | High school | 0.87 (0.76-0.99) | 0.03 | High school | 0.87 (0.76-0.99) | 0.034 |
|  |  | Middle school | 1.00 (0.88-1.14) | 0.992 | Middle school | 1.01 (0.89-1.16) | 0.837 |
|  |  | Primary school or less | 1.24 (1.13-1.36) | <0.001 | Primary school or less | 1.20 (1.09-1.32) | <0.001 |
|  | 1950-1959 | College Education | ref | - | College Education | ref | - |
|  |  | High school | 1.03 (0.80-1.31) | 0.844 | High school | 1.05 (0.82-1.34) | 0.72 |
|  |  | Middle school | 1.11 (0.88-1.41) | 0.375 | Middle school | 1.16 (0.92-1.47) | 0.219 |
|  |  | Primary school or less | 1.55 (1.27-1.89) | <0.001 | Primary school or less | 1.55 (1.28-1.89) | <0.001 |
|  | post-1960 | College Education | ref | - | College Education | ref | - |
|  |  | High school | 1.22 (0.73-2.03) | 0.454 | High school | 1.20 (0.72-2.00) | 0.49 |
|  |  | Middle school | 1.26 (0.82-1.92) | 0.294 | Middle school | 1.26 (0.82-1.94) | 0.282 |
|  |  | Primary school or less | 2.37 (1.53-3.67) | <0.001 | Primary school or less | 2.42 (1.56-3.76) | <0.001 |

*The Basic Adjustment model was adjusted for age, sex, ethnicity, and polygenic risk score for type 2 diabetes. Abbreviations: T2DM, Type 2 diabetes mellitus; HR, hazard ratio; CI, confidence interval.*

Supplementary Table 21. Hazard ratios for transitions between health, T2DM, and CVD according to education level in a multi-state model starting at age 30.

| Transition | Period | Unadjusted | | | Basic Adjusted | | |
| --- | --- | --- | --- | --- | --- | --- | --- |
|  |  | Education | HR (95% CI) | P value |  | HR (95% CI) | P value |
| Baseline to T2DM | Total | College Education | ref | – | College Education | ref | – |
|  |  | High school | 0.98 (0.93-1.04) | 0.526 | High school | 1.05 (1.00-1.11) | 0.052 |
|  |  | Middle school | 1.14 (1.09-1.20) | <0.001 | Middle school | 1.21 (1.15-1.27) | <0.001 |
|  |  | Primary school or less | 1.23 (1.17-1.29) | <0.001 | Primary school or less | 1.21 (1.15-1.27) | <0.001 |
|  | pre-1949 | College Education | ref | - | College Education | ref | - |
|  |  | High school | 1.00 (0.91-1.10) | 0.981 | High school | 1.09 (0.98-1.20) | 0.1 |
|  |  | Middle school | 0.95 (0.85-1.05) | 0.292 | Middle school | 1.05 (0.95-1.17) | 0.333 |
|  |  | Primary school or less | 1.25 (1.15-1.36) | <0.001 | Primary school or less | 1.25 (1.16-1.36) | <0.001 |
|  | 1950-1959 | College Education | ref | - | College Education | ref | - |
|  |  | High school | 1.07 (0.99-1.16) | 0.104 | High school | 1.11 (1.03-1.21) | 0.008 |
|  |  | Middle school | 1.21 (1.12-1.30) | <0.001 | Middle school | 1.25 (1.16-1.36) | <0.001 |
|  |  | Primary school or less | 1.91 (1.77-2.06) | <0.001 | Primary school or less | 1.73 (1.60-1.87) | <0.001 |
|  | post-1960 | College Education | ref | - | College Education | ref | - |
|  |  | High school | 1.15 (1.05-1.26) | 0.004 | High school | 1.20 (1.10-1.32) | <0.001 |
|  |  | Middle school | 1.40 (1.29-1.52) | <0.001 | Middle school | 1.40 (1.29-1.52) | <0.001 |
|  |  | Primary school or less | 2.98 (2.69-3.31) | <0.001 | Primary school or less | 2.30 (2.07-2.55) | <0.001 |
| T2DM to CVD | Total | College Education | ref | – | College Education | ref | – |
|  |  | High school | 1.06 (1.02-1.10) | 0.001 | High school | 1.16 (1.12-1.20) | <0.001 |
|  |  | Middle school | 1.12 (1.08-1.16) | <0.001 | Middle school | 1.26 (1.22-1.31) | <0.001 |
|  |  | Primary school or less | 1.86 (1.81-1.92) | <0.001 | Primary school or less | 1.93 (1.87-1.98) | <0.001 |
|  | pre-1949 | College Education | ref | - | College Education | ref | - |
|  |  | High school | 1.01 (0.96-1.06) | 0.765 | High school | 1.15 (1.09-1.21) | <0.001 |
|  |  | Middle school | 0.99 (0.94-1.04) | 0.598 | Middle school | 1.20 (1.14-1.27) | <0.001 |
|  |  | Primary school or less | 1.58 (1.52-1.64) | <0.001 | Primary school or less | 1.72 (1.66-1.79) | <0.001 |
|  | 1950-1959 | College Education | ref | - | College Education | ref | - |
|  |  | High school | 1.05 (0.99-1.12) | 0.114 | High school | 1.13 (1.06-1.20) | <0.001 |
|  |  | Middle school | 1.16 (1.09-1.23) | <0.001 | Middle school | 1.27 (1.20-1.35) | <0.001 |
|  |  | Primary school or less | 2.08 (1.97-2.20) | <0.001 | Primary school or less | 2.08 (1.96-2.20) | <0.001 |
|  | post-1960 | College Education | ref | - | College Education | ref | - |
|  |  | High school | 1.09 (1.00-1.18) | 0.062 | High school | 1.16 (1.06-1.26) | 0.001 |
|  |  | Middle school | 1.34 (1.24-1.44) | <0.001 | Middle school | 1.38 (1.28-1.49) | <0.001 |
|  |  | Primary school or less | 2.75 (2.50-3.03) | <0.001 | Primary school or less | 2.52 (2.28-2.78) | <0.001 |
| Baseline to CVD | Total | College Education | ref | – | College Education | ref | – |
|  |  | High school | 1.02 (0.87-1.19) | 0.815 | High school | 1.05 (0.90-1.22) | 0.561 |
|  |  | Middle school | 1.19 (1.03-1.37) | 0.015 | Middle school | 1.23 (1.07-1.42) | 0.004 |
|  |  | Primary school or less | 1.47 (1.29-1.67) | <0.001 | Primary school or less | 1.47 (1.29-1.67) | <0.001 |
|  | pre-1949 | College Education | ref | - | College Education | ref | - |
|  |  | High school | 0.94 (0.71-1.26) | 0.687 | High school | 0.97 (0.73-1.30) | 0.859 |
|  |  | Middle school | 0.97 (0.71-1.32) | 0.85 | Middle school | 1.02 (0.75-1.39) | 0.907 |
|  |  | Primary school or less | 1.24 (0.99-1.55) | 0.063 | Primary school or less | 1.27 (1.01-1.59) | 0.039 |
|  | 1950-1959 | College Education | ref | - | College Education | ref | - |
|  |  | High school | 0.98 (0.76-1.27) | 0.9 | High school | 1.00 (0.78-1.30) | 0.97 |
|  |  | Middle school | 1.39 (1.11-1.74) | 0.004 | Middle school | 1.42 (1.13-1.78) | 0.002 |
|  |  | Primary school or less | 1.46 (1.18-1.81) | <0.001 | Primary school or less | 1.44 (1.16-1.78) | 0.001 |
|  | post-1960 | College Education | ref | - | College Education | ref | - |
|  |  | High school | 1.06 (0.81-1.38) | 0.69 | High school | 1.07 (0.82-1.39) | 0.635 |
|  |  | Middle school | 1.18 (0.94-1.46) | 0.147 | Middle school | 1.21 (0.97-1.50) | 0.091 |
|  |  | Primary school or less | 1.38 (1.06-1.78) | 0.016 | Primary school or less | 1.38 (1.06-1.79) | 0.015 |

*The Basic Adjustment model was adjusted for sex, ethnicity, and polygenic risk score for type 2 diabetes. Abbreviations: T2DM, Type 2 diabetes mellitus; CVD, Cardiovascular disease; HR, hazard ratio; CI, confidence interval.*

Supplementary Table 22. Transition-specific risk-set sizes and event counts in the multi-state models.

| Process | Transition | Time scale | Risk-set size, n | Events, n | Person-years | Median risk interval, years |
| --- | --- | --- | --- | --- | --- | --- |
| Mortality pathway | Baseline -> T2DM | Time since baseline, years | 460,797 | 19,931 | 7,264,116.2 | 16.57 |
|  | Baseline -> Death | Time since baseline, years | 460,797 | 33,637 | 7,264,116.2 | 16.57 |
|  | T2DM -> Death | Time since baseline, years | 19,931 | 3,163 | 150,376.0 | 7.32 |
| CVD pathway | Baseline -> T2DM | Time since baseline, years | 460,797 | 17,890 | 6,946,128.3 | 16.42 |
|  | Baseline -> CVD | Time since baseline, years | 460,797 | 43,827 | 6,946,128.3 | 16.42 |
|  | T2DM -> CVD | Time since baseline, years | 17,890 | 2,826 | 121,759.0 | 6.58 |

*The time scale was time since baseline. Risk-set sizes and event counts were calculated from the counting-process data for each transition. Same-day T2DM/CVD and same-day T2DM/death events were retained by assigning T2DM as occurring first and shifting the downstream event time to avoid zero-length intervals. CVD events occurring before T2DM contributed to the baseline-to-CVD transition and were not included in the T2DM-to-CVD transition risk set. T2DM, type 2 diabetes mellitus; CVD, cardiovascular disease.*

Supplementary Table 23. Proportion of the association between education level and T2DM risk mediated by socioeconomic, behavioral, and metabolic factors, individually and jointly, stratified by birth year and sex.

| Period Group | Sex Group | Model | HR and 95% CI | PTE and 95% CI | P |
| --- | --- | --- | --- | --- | --- |
| Overall | Overall | Base | 2.44 (2.32-2.57) | - | - |
|  |  | Socioeconomic factors | 1.67 (1.59-1.77) | 42.2% (39.2, 45.2) | <0.001 |
|  |  | Healthy lifestyles | 2.03 (1.93-2.14) | 20.5% (18.9, 22.2) | <0.001 |
|  |  | Metabolic factors | 1.67 (1.59-1.75) | 42.6% (38.2, 47.3) | <0.001 |
|  |  | Socioeconomic factors | 1.67 (1.59-1.77) | 42.2% (39.2, 45.2) | <0.001 |
|  |  | Socioeconomic factors + Healthy lifestyles | 1.55 (1.47-1.63) | 50.9% (47.7, 54.4) | <0.001 |
|  |  | Socioeconomic factors + Healthy lifestyles + Metabolic factors | 1.30 (1.23-1.37) | 70.5% (65.0, 76.0) | <0.001 |
|  | Female | Base | 2.41 (2.25-2.57) | - | - |
|  |  | Socioeconomic factors | 1.63 (1.52-1.75) | 44.2% (40.3, 48.7) | <0.001 |
|  |  | Healthy lifestyles | 2.05 (1.91-2.19) | 18.6% (16.3, 20.9) | <0.001 |
|  |  | Metabolic factors | 1.59 (1.49-1.70) | 47.1% (42.2, 52.6) | <0.001 |
|  |  | Socioeconomic factors | 1.63 (1.52-1.75) | 44.2% (40.3, 48.7) | <0.001 |
|  |  | Socioeconomic factors + Healthy lifestyles | 1.53 (1.43-1.64) | 51.5% (47.2, 56.9) | <0.001 |
|  |  | Socioeconomic factors + Healthy lifestyles + Metabolic factors | 1.23 (1.14-1.31) | 76.9% (70.2, 84.2) | <0.001 |
|  | Male | Base | 2.49 (2.30-2.70) | - | - |
|  |  | Socioeconomic factors | 1.73 (1.60-1.88) | 39.6% (35.7, 44.2) | <0.001 |
|  |  | Healthy lifestyles | 1.98 (1.82-2.14) | 25.2% (22.6, 28.2) | <0.001 |
|  |  | Metabolic factors | 1.79 (1.65-1.94) | 36.2% (30.6, 43.1) | <0.001 |
|  |  | Socioeconomic factors | 1.73 (1.60-1.88) | 39.6% (35.7, 44.2) | <0.001 |
|  |  | Socioeconomic factors + Healthy lifestyles | 1.54 (1.42-1.67) | 52.9% (48.0, 58.4) | <0.001 |
|  |  | Socioeconomic factors + Healthy lifestyles + Metabolic factors | 1.41 (1.30-1.53) | 62.3% (55.7, 70.7) | <0.001 |
| pre-1949 | Overall | Base | 2.39 (2.24-2.56) | - | - |
|  |  | Socioeconomic factors | 1.76 (1.64-1.88) | 35.6% (32.0, 39.8) | <0.001 |
|  |  | Healthy lifestyles | 2.06 (1.92-2.20) | 17.3% (15.3, 19.4) | <0.001 |
|  |  | Metabolic factors | 1.68 (1.57-1.80) | 40.4% (34.5, 48.0) | <0.001 |
|  |  | Socioeconomic factors | 1.76 (1.64-1.88) | 35.6% (32.0, 39.8) | <0.001 |
|  |  | Socioeconomic factors + Healthy lifestyles | 1.62 (1.51-1.74) | 44.4% (40.4, 49.3) | <0.001 |
|  |  | Socioeconomic factors + Healthy lifestyles + Metabolic factors | 1.37 (1.28-1.47) | 64.1% (57.5, 72.4) | <0.001 |
|  | Female | Base | 2.40 (2.20-2.62) | - | - |
|  |  | Socioeconomic factors | 1.68 (1.53-1.84) | 41.0% (35.4, 47.1) | <0.001 |
|  |  | Healthy lifestyles | 2.10 (1.92-2.29) | 15.4% (13.0, 18.0) | <0.001 |
|  |  | Metabolic factors | 1.61 (1.48-1.76) | 45.3% (39.3, 52.0) | <0.001 |
|  |  | Socioeconomic factors | 1.68 (1.53-1.84) | 41.0% (35.4, 47.1) | <0.001 |
|  |  | Socioeconomic factors + Healthy lifestyles | 1.58 (1.44-1.73) | 47.9% (41.6, 54.8) | <0.001 |
|  |  | Socioeconomic factors + Healthy lifestyles + Metabolic factors | 1.27 (1.16-1.39) | 72.8% (64.4, 82.6) | <0.001 |
|  | Male | Base | 2.39 (2.15-2.66) | - | - |
|  |  | Socioeconomic factors | 1.86 (1.67-2.08) | 28.7% (23.9, 34.2) | <0.001 |
|  |  | Healthy lifestyles | 1.97 (1.77-2.19) | 22.4% (18.9, 26.0) | <0.001 |
|  |  | Metabolic factors | 1.77 (1.59-1.97) | 34.4% (26.5, 45.9) | <0.001 |
|  |  | Socioeconomic factors | 1.86 (1.67-2.08) | 28.7% (23.9, 34.2) | <0.001 |
|  |  | Socioeconomic factors + Healthy lifestyles | 1.64 (1.47-1.84) | 42.9% (37.3, 50.1) | <0.001 |
|  |  | Socioeconomic factors + Healthy lifestyles + Metabolic factors | 1.49 (1.34-1.66) | 54.2% (46.3, 65.9) | <0.001 |
| 1950-1959 | Overall | Base | 2.56 (2.33-2.82) | - | - |
|  |  | Socioeconomic factors | 1.61 (1.46-1.78) | 49.1% (43.1, 55.6) | <0.001 |
|  |  | Healthy lifestyles | 2.05 (1.87-2.26) | 23.5% (20.2, 26.9) | <0.001 |
|  |  | Metabolic factors | 1.70 (1.55-1.87) | 43.3% (37.2, 49.7) | <0.001 |
|  |  | Socioeconomic factors | 1.61 (1.46-1.78) | 49.1% (43.1, 55.6) | <0.001 |
|  |  | Socioeconomic factors + Healthy lifestyles | 1.49 (1.35-1.64) | 57.6% (50.6, 65.0) | <0.001 |
|  |  | Socioeconomic factors + Healthy lifestyles + Metabolic factors | 1.26 (1.14-1.39) | 75.5% (66.6, 85.0) | <0.001 |
|  | Female | Base | 2.52 (2.22-2.85) | - | - |
|  |  | Socioeconomic factors | 1.62 (1.42-1.85) | 47.6% (40.5, 55.9) | <0.001 |
|  |  | Healthy lifestyles | 2.06 (1.82-2.34) | 21.6% (17.5, 27.0) | <0.001 |
|  |  | Metabolic factors | 1.62 (1.43-1.83) | 47.7% (40.0, 57.7) | <0.001 |
|  |  | Socioeconomic factors | 1.62 (1.42-1.85) | 47.6% (40.5, 55.9) | <0.001 |
|  |  | Socioeconomic factors + Healthy lifestyles | 1.51 (1.33-1.72) | 55.2% (47.2, 65.0) | <0.001 |
|  |  | Socioeconomic factors + Healthy lifestyles + Metabolic factors | 1.21 (1.06-1.37) | 79.6% (68.4, 95.0) | <0.001 |
|  | Male | Base | 2.64 (2.28-3.06) | - | - |
|  |  | Socioeconomic factors | 1.62 (1.39-1.88) | 50.4% (42.7, 60.3) | <0.001 |
|  |  | Healthy lifestyles | 2.02 (1.74-2.34) | 27.6% (23.1, 33.6) | <0.001 |
|  |  | Metabolic factors | 1.81 (1.57-2.09) | 39.0% (30.4, 48.8) | <0.001 |
|  |  | Socioeconomic factors | 1.62 (1.39-1.88) | 50.4% (42.7, 60.3) | <0.001 |
|  |  | Socioeconomic factors + Healthy lifestyles | 1.45 (1.24-1.68) | 62.0% (53.0, 73.8) | <0.001 |
|  |  | Socioeconomic factors + Healthy lifestyles + Metabolic factors | 1.33 (1.15-1.55) | 70.4% (58.7, 86.3) | <0.001 |
| post-1960 | Overall | Base | 2.55 (2.21-2.95) | - | - |
|  |  | Socioeconomic factors | 1.54 (1.33-1.78) | 54.1% (45.5, 64.5) | <0.001 |
|  |  | Healthy lifestyles | 2.00 (1.73-2.31) | 26.0% (21.2, 32.0) | <0.001 |
|  |  | Metabolic factors | 1.62 (1.40-1.87) | 48.5% (39.1, 59.2) | <0.001 |
|  |  | Socioeconomic factors | 1.54 (1.33-1.78) | 54.1% (45.5, 64.5) | <0.001 |
|  |  | Socioeconomic factors + Healthy lifestyles | 1.42 (1.23-1.65) | 62.3% (52.5, 74.7) | <0.001 |
|  |  | Socioeconomic factors + Healthy lifestyles + Metabolic factors | 1.19 (1.02-1.38) | 81.8% (68.2, 98.7) | <0.001 |
|  | Female | Base | 2.49 (2.06-3.01) | - | - |
|  |  | Socioeconomic factors | 1.54 (1.27-1.88) | 52.5% (41.2, 68.9) | <0.001 |
|  |  | Healthy lifestyles | 2.01 (1.66-2.44) | 23.4% (17.2, 31.5) | <0.001 |
|  |  | Metabolic factors | 1.54 (1.27-1.86) | 52.6% (40.3, 68.9) | <0.001 |
|  |  | Socioeconomic factors | 1.54 (1.27-1.88) | 52.5% (41.2, 68.9) | <0.001 |
|  |  | Socioeconomic factors + Healthy lifestyles | 1.44 (1.18-1.75) | 60.0% (47.0, 78.4) | <0.001 |
|  |  | Socioeconomic factors + Healthy lifestyles + Metabolic factors | 1.12 (0.92-1.37) | 87.4% (69.8, 113.3) | <0.001 |
|  | Male | Base | 2.64 (2.12-3.30) | - | - |
|  |  | Socioeconomic factors | 1.53 (1.22-1.91) | 56.3% (44.7, 74.1) | <0.001 |
|  |  | Healthy lifestyles | 1.96 (1.57-2.45) | 30.8% (23.3, 41.6) | <0.001 |
|  |  | Metabolic factors | 1.69 (1.36-2.10) | 46.2% (32.9, 62.6) | <0.001 |
|  |  | Socioeconomic factors | 1.53 (1.22-1.91) | 56.3% (44.7, 74.1) | <0.001 |
|  |  | Socioeconomic factors + Healthy lifestyles | 1.38 (1.10-1.73) | 66.9% (53.3, 88.0) | <0.001 |
|  |  | Socioeconomic factors + Healthy lifestyles + Metabolic factors | 1.26 (1.00-1.58) | 76.5% (59.0, 102.4) | <0.001 |

*Abbreviations: HR, hazard ratio; CI, confidence interval; PTE, proportion of total effect.*

Supplementary Table 24. Proportion of the association between education level and T2DM risk mediated by specific socioeconomic, behavioral, and metabolic risk factors, stratified by birth year and sex.

| Period Group | Sex Group | Model | HR and 95% CI | PTE and 95% CI | P |
| --- | --- | --- | --- | --- | --- |
| Overall | Overall | Base | 2.44 (2.32-2.57) |  |  |
|  |  | Townsend Deprivation Index | 2.15 (2.04-2.26) | 14.3% (13.2, 15.5) | <0.001 |
|  |  | Household Income | 1.82 (1.72-1.91) | 33.1% (30.7, 35.8) | <0.001 |
|  |  | Marriage | 2.42 (2.30-2.54) | 0.9% (0.7, 1.2) | <0.001 |
|  |  | Employment Status | 2.21 (2.10-2.33) | 11.0% (10.0, 12.1) | <0.001 |
|  |  | Alcohol Intake | 2.37 (2.25-2.49) | 3.4% (2.9, 3.9) | <0.001 |
|  |  | Dietary Habits | 2.36 (2.24-2.48) | 3.8% (3.2, 4.3) | <0.001 |
|  |  | Sleep Habits | 2.28 (2.17-2.40) | 7.4% (6.6, 8.1) | <0.001 |
|  |  | Physical Activity | 2.46 (2.33-2.58) | -0.8% (-1.2, -0.5) | <0.001 |
|  |  | Smoking Status | 2.27 (2.16-2.39) | 8.1% (7.3, 8.9) | <0.001 |
|  |  | BMI | 1.97 (1.87-2.07) | 23.8% (21.7, 26.0) | <0.001 |
|  |  | HbA1c | 1.98 (1.88-2.08) | 23.3% (19.1, 28.0) | <0.001 |
|  |  | Systolic Blood Pressure | 2.36 (2.25-2.49) | 3.4% (3.0, 3.9) | <0.001 |
|  |  | HDL-C | 2.12 (2.02-2.23) | 15.6% (14.4, 16.9) | <0.001 |
|  |  | Triglycerides | 2.26 (2.15-2.38) | 8.5% (7.4, 9.7) | <0.001 |
|  | Female | Base | 2.41 (2.25-2.57) |  |  |
|  |  | Townsend Deprivation Index | 2.26 (2.08-2.44) | 10.8% (9.6, 12.2) | <0.001 |
|  |  | Household Income | 1.90 (1.75-2.06) | 29.8% (26.7, 33.7) | <0.001 |
|  |  | Marriage | 2.48 (2.29-2.69) | 0.3% (0.1, 0.5) | <0.001 |
|  |  | Employment Status | 2.28 (2.10-2.47) | 9.7% (8.3, 11.3) | <0.001 |
|  |  | Alcohol Intake | 2.32 (2.14-2.51) | 7.7% (6.6, 8.9) | <0.001 |
|  |  | Dietary Habits | 2.41 (2.22-2.61) | 3.7% (2.9, 4.6) | <0.001 |
|  |  | Sleep Habits | 2.34 (2.16-2.54) | 6.7% (5.7, 7.9) | <0.001 |
|  |  | Physical Activity | 2.46 (2.27-2.67) | 1.1% (0.7, 1.5) | <0.001 |
|  |  | Smoking Status | 2.34 (2.16-2.53) | 6.9% (5.9, 8.1) | <0.001 |
|  |  | BMI | 2.06 (1.90-2.23) | 21.0% (17.9, 24.3) | <0.001 |
|  |  | HbA1c | 2.26 (2.09-2.45) | 10.5% (3.8, 20.1) | <0.001 |
|  |  | Systolic Blood Pressure | 2.38 (2.20-2.58) | 4.8% (4.0, 5.5) | <0.001 |
|  |  | HDL-C | 2.00 (1.84-2.16) | 24.2% (21.6, 26.8) | <0.001 |
|  |  | Triglycerides | 2.20 (2.03-2.38) | 13.5% (11.4, 15.8) | <0.001 |
|  | Male | Base | 2.49 (2.30-2.70) |  |  |
|  |  | Townsend Deprivation Index | 2.09 (1.96-2.23) | 16.1% (14.3, 18.1) | <0.001 |
|  |  | Household Income | 1.77 (1.65-1.90) | 35.1% (31.6, 39.3) | <0.001 |
|  |  | Marriage | 2.37 (2.22-2.54) | 1.6% (1.1, 2.1) | <0.001 |
|  |  | Employment Status | 2.15 (2.01-2.30) | 12.9% (11.3, 14.7) | <0.001 |
|  |  | Alcohol Intake | 2.37 (2.22-2.54) | 1.6% (1.0, 2.2) | <0.001 |
|  |  | Dietary Habits | 2.33 (2.18-2.49) | 3.9% (3.2, 4.7) | <0.001 |
|  |  | Sleep Habits | 2.25 (2.10-2.40) | 7.8% (6.7, 9.0) | <0.001 |
|  |  | Physical Activity | 2.46 (2.30-2.63) | -2.4% (-3.0, -1.8) | <0.001 |
|  |  | Smoking Status | 2.21 (2.07-2.37) | 9.5% (8.4, 10.9) | <0.001 |
|  |  | BMI | 1.88 (1.76-2.01) | 28.1% (25.0, 31.6) | <0.001 |
|  |  | HbA1c | 1.84 (1.73-1.97) | 30.3% (26.9, 33.9) | <0.001 |
|  |  | Systolic Blood Pressure | 2.36 (2.21-2.52) | 2.3% (1.8, 2.8) | <0.001 |
|  |  | HDL-C | 2.21 (2.07-2.36) | 9.9% (8.7, 11.3) | <0.001 |
|  |  | Triglycerides | 2.30 (2.15-2.45) | 5.4% (4.3, 6.6) | <0.001 |
| pre-1949 | Overall | Base | 2.39 (2.24-2.56) |  |  |
|  |  | Townsend Deprivation Index | 2.14 (2.00-2.29) | 12.7% (11.2, 14.5) | <0.001 |
|  |  | Household Income | 1.89 (1.77-2.03) | 26.9% (23.4, 30.8) | <0.001 |
|  |  | Marriage | 2.38 (2.23-2.55) | 0.6% (0.4, 0.9) | <0.001 |
|  |  | Employment Status | 2.28 (2.13-2.43) | 5.8% (4.7, 7.0) | <0.001 |
|  |  | Alcohol Intake | 2.33 (2.18-2.49) | 3.1% (2.4, 3.8) | <0.001 |
|  |  | Dietary Habits | 2.32 (2.17-2.48) | 3.7% (3.0, 4.5) | <0.001 |
|  |  | Sleep Habits | 2.28 (2.13-2.44) | 5.6% (4.8, 6.6) | <0.001 |
|  |  | Physical Activity | 2.41 (2.25-2.57) | -0.6% (-1.0, -0.2) | 0.01 |
|  |  | Smoking Status | 2.26 (2.12-2.42) | 6.4% (5.5, 7.5) | <0.001 |
|  |  | BMI | 1.93 (1.80-2.06) | 24.8% (22.4, 27.5) | <0.001 |
|  |  | HbA1c | 2.00 (1.88-2.14) | 20.3% (14.5, 26.7) | <0.001 |
|  |  | Systolic Blood Pressure | 2.35 (2.20-2.52) | 1.9% (1.5, 2.4) | <0.001 |
|  |  | HDL-C | 2.08 (1.95-2.23) | 16.0% (14.4, 17.8) | <0.001 |
|  |  | Triglycerides | 2.24 (2.10-2.40) | 7.6% (6.3, 9.0) | <0.001 |
|  | Female | Base | 2.40 (2.20-2.62) |  |  |
|  |  | Townsend Deprivation Index | 2.21 (1.99-2.46) | 9.0% (7.4, 10.8) | <0.001 |
|  |  | Household Income | 1.96 (1.76-2.19) | 22.7% (18.4, 27.2) | <0.001 |
|  |  | Marriage | 2.39 (2.15-2.66) | -0.1% (-0.4, 0.1) | 0.19 |
|  |  | Employment Status | 2.33 (2.09-2.59) | 3.1% (1.9, 4.5) | <0.001 |
|  |  | Alcohol Intake | 2.22 (2.00-2.48) | 8.3% (6.7, 10.1) | <0.001 |
|  |  | Dietary Habits | 2.32 (2.08-2.58) | 3.6% (2.5, 4.8) | <0.001 |
|  |  | Sleep Habits | 2.29 (2.06-2.55) | 4.7% (3.5, 6.1) | <0.001 |
|  |  | Physical Activity | 2.37 (2.13-2.64) | 1.1% (0.5, 1.6) | <0.001 |
|  |  | Smoking Status | 2.28 (2.05-2.54) | 5.2% (4.0, 6.5) | <0.001 |
|  |  | BMI | 1.97 (1.77-2.19) | 22.4% (18.9, 26.2) | <0.001 |
|  |  | HbA1c | 2.24 (2.01-2.49) | 7.6% (-0.6, 20.6) | 0.072 |
|  |  | Systolic Blood Pressure | 2.33 (2.10-2.59) | 2.9% (2.1, 3.7) | <0.001 |
|  |  | HDL-C | 1.91 (1.72-2.13) | 25.5% (22.2, 29.6) | <0.001 |
|  |  | Triglycerides | 2.14 (1.92-2.38) | 12.6% (10.1, 15.4) | <0.001 |
|  | Male | Base | 2.39 (2.15-2.66) |  |  |
|  |  | Townsend Deprivation Index | 2.11 (1.93-2.30) | 14.9% (12.5, 17.5) | <0.001 |
|  |  | Household Income | 1.85 (1.69-2.03) | 29.8% (25.3, 35.5) | <0.001 |
|  |  | Marriage | 2.37 (2.18-2.59) | 1.3% (0.8, 2.0) | <0.001 |
|  |  | Employment Status | 2.22 (2.04-2.42) | 8.8% (7.0, 10.6) | <0.001 |
|  |  | Alcohol Intake | 2.38 (2.19-2.60) | 0.8% (0.1, 1.6) | 0.024 |
|  |  | Dietary Habits | 2.32 (2.13-2.53) | 3.8% (2.8, 4.8) | <0.001 |
|  |  | Sleep Habits | 2.27 (2.08-2.48) | 6.3% (5.0, 7.8) | <0.001 |
|  |  | Physical Activity | 2.45 (2.25-2.67) | -2.1% (-2.9, -1.4) | <0.001 |
|  |  | Smoking Status | 2.24 (2.06-2.44) | 7.9% (6.5, 9.5) | <0.001 |
|  |  | BMI | 1.88 (1.73-2.05) | 27.9% (24.1, 31.9) | <0.001 |
|  |  | HbA1c | 1.87 (1.72-2.04) | 28.5% (24.2, 33.7) | <0.001 |
|  |  | Systolic Blood Pressure | 2.38 (2.18-2.59) | 1.2% (0.7, 1.9) | <0.001 |
|  |  | HDL-C | 2.20 (2.02-2.39) | 10.1% (8.4, 11.8) | <0.001 |
|  |  | Triglycerides | 2.30 (2.11-2.50) | 5.0% (3.5, 6.5) | <0.001 |
| 1950-1959 | Overall | Base | 2.56 (2.33-2.82) |  |  |
|  |  | Townsend Deprivation Index | 2.23 (2.03-2.46) | 14.5% (12.7, 16.8) | <0.001 |
|  |  | Household Income | 1.80 (1.63-1.98) | 37.8% (33.0, 43.3) | <0.001 |
|  |  | Marriage | 2.55 (2.32-2.80) | 0.5% (0.2, 0.9) | <0.001 |
|  |  | Employment Status | 2.15 (1.96-2.37) | 18.5% (16.0, 21.9) | <0.001 |
|  |  | Alcohol Intake | 2.49 (2.27-2.74) | 3.0% (2.2, 4.0) | <0.001 |
|  |  | Dietary Habits | 2.47 (2.24-2.71) | 4.0% (3.1, 5.2) | <0.001 |
|  |  | Sleep Habits | 2.36 (2.15-2.60) | 8.6% (7.3, 10.3) | <0.001 |
|  |  | Physical Activity | 2.58 (2.35-2.84) | -0.9% (-1.6, -0.3) | 0.006 |
|  |  | Smoking Status | 2.32 (2.11-2.55) | 10.7% (9.1, 12.6) | <0.001 |
|  |  | BMI | 2.08 (1.90-2.29) | 22.0% (17.5, 27.2) | <0.001 |
|  |  | HbA1c | 1.99 (1.82-2.19) | 26.6% (22.5, 31.1) | <0.001 |
|  |  | Systolic Blood Pressure | 2.44 (2.22-2.68) | 5.2% (4.3, 6.3) | <0.001 |
|  |  | HDL-C | 2.27 (2.06-2.49) | 13.0% (11.1, 15.1) | <0.001 |
|  |  | Triglycerides | 2.37 (2.16-2.61) | 8.3% (6.4, 10.5) | <0.001 |
|  | Female | Base | 2.52 (2.22-2.85) |  |  |
|  |  | Townsend Deprivation Index | 2.36 (2.04-2.73) | 11.6% (9.5, 14.2) | <0.001 |
|  |  | Household Income | 1.89 (1.62-2.19) | 34.7% (29.0, 42.2) | <0.001 |
|  |  | Marriage | 2.64 (2.28-3.06) | 0.0% (-0.1, 0.2) | 0.668 |
|  |  | Employment Status | 2.21 (1.90-2.56) | 18.4% (14.8, 22.8) | <0.001 |
|  |  | Alcohol Intake | 2.47 (2.14-2.86) | 6.7% (5.1, 8.6) | <0.001 |
|  |  | Dietary Habits | 2.53 (2.19-2.93) | 4.4% (3.1, 6.1) | <0.001 |
|  |  | Sleep Habits | 2.45 (2.12-2.84) | 7.7% (6.0, 9.9) | <0.001 |
|  |  | Physical Activity | 2.62 (2.26-3.03) | 0.9% (0.2, 1.8) | 0.022 |
|  |  | Smoking Status | 2.42 (2.09-2.81) | 8.8% (6.8, 11.3) | <0.001 |
|  |  | BMI | 2.21 (1.91-2.55) | 18.4% (12.7, 25.7) | <0.001 |
|  |  | HbA1c | 2.20 (1.90-2.54) | 18.9% (13.0, 25.2) | <0.001 |
|  |  | Systolic Blood Pressure | 2.47 (2.13-2.86) | 7.0% (5.4, 9.0) | <0.001 |
|  |  | HDL-C | 2.18 (1.88-2.52) | 19.8% (16.4, 24.3) | <0.001 |
|  |  | Triglycerides | 2.26 (1.96-2.62) | 15.9% (11.5, 21.2) | <0.001 |
|  | Male | Base | 2.64 (2.28-3.06) |  |  |
|  |  | Townsend Deprivation Index | 2.18 (1.92-2.47) | 15.7% (12.7, 19.4) | <0.001 |
|  |  | Household Income | 1.74 (1.53-1.98) | 39.8% (33.6, 47.9) | <0.001 |
|  |  | Marriage | 2.50 (2.20-2.83) | 0.9% (-0.0, 1.9) | 0.054 |
|  |  | Employment Status | 2.13 (1.87-2.41) | 18.3% (14.9, 22.9) | <0.001 |
|  |  | Alcohol Intake | 2.48 (2.19-2.81) | 1.5% (0.3, 2.7) | 0.012 |
|  |  | Dietary Habits | 2.44 (2.15-2.76) | 3.5% (2.2, 5.1) | <0.001 |
|  |  | Sleep Habits | 2.31 (2.04-2.62) | 9.3% (7.2, 11.8) | <0.001 |
|  |  | Physical Activity | 2.57 (2.27-2.91) | -2.3% (-3.4, -1.3) | <0.001 |
|  |  | Smoking Status | 2.24 (1.98-2.54) | 12.5% (10.1, 15.6) | <0.001 |
|  |  | BMI | 1.95 (1.72-2.20) | 27.8% (22.5, 34.5) | <0.001 |
|  |  | HbA1c | 1.88 (1.67-2.13) | 31.4% (25.5, 38.1) | <0.001 |
|  |  | Systolic Blood Pressure | 2.43 (2.15-2.75) | 3.8% (2.7, 5.1) | <0.001 |
|  |  | HDL-C | 2.34 (2.06-2.64) | 8.2% (5.9, 10.6) | <0.001 |
|  |  | Triglycerides | 2.41 (2.13-2.73) | 4.8% (2.8, 7.0) | <0.001 |
| post-1960 | Overall | Base | 2.55 (2.21-2.95) |  |  |
|  |  | Townsend Deprivation Index | 2.20 (1.90-2.53) | 16.0% (13.2, 19.3) | <0.001 |
|  |  | Household Income | 1.69 (1.46-1.96) | 43.8% (36.9, 52.5) | <0.001 |
|  |  | Marriage | 2.46 (2.13-2.84) | 3.8% (2.5, 5.3) | <0.001 |
|  |  | Employment Status | 2.13 (1.84-2.47) | 19.1% (15.7, 23.2) | <0.001 |
|  |  | Alcohol Intake | 2.43 (2.11-2.81) | 5.0% (3.3, 6.9) | <0.001 |
|  |  | Dietary Habits | 2.48 (2.14-2.86) | 3.2% (1.8, 4.6) | <0.001 |
|  |  | Sleep Habits | 2.30 (1.99-2.65) | 11.3% (9.0, 14.1) | <0.001 |
|  |  | Physical Activity | 2.57 (2.23-2.97) | -1.0% (-2.0, 0.0) | 0.052 |
|  |  | Smoking Status | 2.32 (2.00-2.68) | 10.3% (7.8, 13.2) | <0.001 |
|  |  | BMI | 2.04 (1.76-2.35) | 24.1% (16.0, 33.1) | <0.001 |
|  |  | HbA1c | 1.90 (1.65-2.20) | 31.3% (24.8, 38.5) | <0.001 |
|  |  | Systolic Blood Pressure | 2.40 (2.08-2.77) | 6.6% (4.8, 8.8) | <0.001 |
|  |  | HDL-C | 2.19 (1.89-2.52) | 16.5% (13.3, 20.4) | <0.001 |
|  |  | Triglycerides | 2.30 (1.99-2.65) | 11.3% (8.0, 15.2) | <0.001 |
|  | Female | Base | 2.49 (2.06-3.01) |  |  |
|  |  | Townsend Deprivation Index | 2.33 (1.88-2.90) | 12.7% (9.7, 16.8) | <0.001 |
|  |  | Household Income | 1.74 (1.39-2.18) | 42.8% (33.6, 55.8) | <0.001 |
|  |  | Marriage | 2.56 (2.05-3.19) | 3.3% (1.7, 5.3) | <0.001 |
|  |  | Employment Status | 2.24 (1.79-2.80) | 17.0% (12.2, 22.5) | <0.001 |
|  |  | Alcohol Intake | 2.49 (1.99-3.10) | 6.2% (3.8, 9.4) | <0.001 |
|  |  | Dietary Habits | 2.59 (2.08-3.24) | 1.8% (0.1, 3.7) | 0.036 |
|  |  | Sleep Habits | 2.32 (1.86-2.90) | 13.3% (9.5, 18.5) | <0.001 |
|  |  | Physical Activity | 2.59 (2.07-3.23) | 2.0% (0.8, 3.5) | 0.002 |
|  |  | Smoking Status | 2.38 (1.91-2.97) | 10.6% (7.2, 14.5) | <0.001 |
|  |  | BMI | 2.12 (1.70-2.63) | 22.8% (9.9, 37.1) | <0.001 |
|  |  | HbA1c | 2.07 (1.66-2.58) | 25.1% (16.4, 34.7) | <0.001 |
|  |  | Systolic Blood Pressure | 2.40 (1.92-3.00) | 9.8% (6.6, 13.9) | <0.001 |
|  |  | HDL-C | 2.05 (1.64-2.56) | 26.1% (19.6, 35.4) | <0.001 |
|  |  | Triglycerides | 2.19 (1.76-2.73) | 19.2% (11.4, 29.1) | <0.001 |
|  | Male | Base | 2.64 (2.12-3.30) |  |  |
|  |  | Townsend Deprivation Index | 2.12 (1.75-2.57) | 17.5% (13.6, 23.1) | <0.001 |
|  |  | Household Income | 1.66 (1.37-2.03) | 44.1% (34.8, 58.8) | <0.001 |
|  |  | Marriage | 2.39 (1.98-2.89) | 4.3% (2.3, 7.0) | <0.001 |
|  |  | Employment Status | 2.04 (1.69-2.47) | 21.7% (16.4, 29.5) | <0.001 |
|  |  | Alcohol Intake | 2.37 (1.96-2.87) | 5.1% (2.7, 8.3) | <0.001 |
|  |  | Dietary Habits | 2.39 (1.98-2.89) | 4.4% (2.7, 6.8) | <0.001 |
|  |  | Sleep Habits | 2.28 (1.88-2.76) | 9.7% (6.8, 13.6) | <0.001 |
|  |  | Physical Activity | 2.56 (2.12-3.10) | -3.2% (-5.2, -1.6) | <0.001 |
|  |  | Smoking Status | 2.27 (1.87-2.74) | 10.3% (7.0, 14.7) | <0.001 |
|  |  | BMI | 1.94 (1.60-2.34) | 27.6% (20.1, 37.6) | <0.001 |
|  |  | HbA1c | 1.80 (1.49-2.17) | 35.5% (26.5, 46.1) | <0.001 |
|  |  | Systolic Blood Pressure | 2.39 (1.98-2.89) | 4.2% (2.5, 6.6) | <0.001 |
|  |  | HDL-C | 2.28 (1.89-2.76) | 9.6% (6.7, 13.6) | <0.001 |
|  |  | Triglycerides | 2.29 (1.89-2.77) | 9.1% (5.5, 13.5) | <0.001 |

*Abbreviations: HR, hazard ratio; CI, confidence interval; PTE, proportion of total effect, BMI, Body mass index; HbA1c, Hemoglobin A1c (Glycated hemoglobin).*
